# The Batten disease protein CLN8 is a stereospecific acyltransferase in bis(monoacylglycero)phosphate biosynthesis

**DOI:** 10.1038/s41556-026-02061-0

**Published:** 2026-09-15

**Authors:** Pradeep K. Sheokand, Denis Lacabanne, Andrew M. James, Stefania Della Vecchia, Jonathan J. Ruprecht, Joris van der Kleij, Keira Turner, Jessica Müller-Niva, Miia H. Salo, Benjamin Jenkins, Susannah K. Leese, Nidhi Juneja, Chak Shun Yu, Clarissa D. Booth, Martin S. King, Johanna Uusimaa, Jill M. Weimer, Albert Koulman, Reetta Hinttala, Filippo M. Santorelli, Maria Marchese, Michael P. Murphy, Edmund R. S. Kunji, Kasparas Petkevicius

**Affiliations:** 1 MRC Mitochondrial Biology Unit, University of Cambridge, Cambridge Biomedical Campus, Cambridge, UK; 2 Neurobiology and Molecular Medicine Unit, IRCCS Fondazione Stella Maris, Calambrone, Pisa, Italy; 3 Department of Neurosciences, Psychology, Drug Research and Child Health, University of Florence, Florence, Italy; 4 Research Unit of Clinical Medicine, Medical Research Center, University of Oulu and Oulu University Hospital, Oulu, Finland; 5 Biocenter Oulu, University of Oulu, Oulu, Finland; 6 Metabolic Research Laboratories, Institute of Metabolic Science, University of Cambridge, Cambridge Biomedical Campus, Cambridge, UK; 7 Center for Genetics and Rare Diseases, Sanford Research, Sioux Falls, SD USA; 8 Department of Children and Adolescents, Division of Paediatric Neurology, Oulu University Hospital, Oulu, Finland; 9 Department of Pediatrics, Sanford School of Medicine, University of South Dakota, Sioux Falls, SD USA; 10 Department of Medicine, University of Cambridge, Cambridge Biomedical Campus, Cambridge, UK

**Keywords:** Phospholipids, Lipidomics, Cryoelectron microscopy, Mechanisms of disease, Enzymes

## Abstract

Loss-of-function mutations in the endoplasmic reticulum membrane protein CLN8 cause Batten disease, a neurodegenerative lysosomal storage disorder. CLN8 acts with the lysosomal enzyme CLN5 to produce bis(monoacylglycero)phosphate (BMP), a signature lysosomal phospholipid with unique *S*,*S* stereochemistry. However, the role of CLN8 in this pathway has remained unclear. Here we establish that CLN8 is a glycerophosphoglycerol acyltransferase that catalyses the stereospecific acylation of *S*,*S*-glycerophosphoglycerol to generate *S*,*S*-lysophosphatidylglycerol, the CLN5 substrate in BMP synthesis. Cryo-electron microscopy structures define the CLN8 active site and support a ping-pong acyl transfer mechanism. Batten disease mutations impair CLN8 enzymatic activity and abolish BMP production in mice. Exogenous *S*,*S*-lysophosphatidylglycerol, but not the *R*,*S* stereoisomer, restores BMP synthesis in CLN8-deficient cells and mice and improves neurological phenotypes in *cln8* mutant zebrafish. These findings define the function of CLN8, explain the biochemical basis of CLN8 Batten disease and establish BMP precursor supplementation as a proof-of-concept therapeutic strategy.

## Abstract (web summary)

Two studies show that the endoplasmic reticulum-associated protein CLN8 catalyses the acyl CoA-dependent acylation of glycerophosphoglycerol, producing lysophosphatidylglycerol, which is then converted into bis(monoacylglycero)phosphate. These results provide insight into CLN8 Batten disease.

## Main

Batten disease, or neuronal ceroid lipofuscinosis, encompasses a group of inherited lysosomal storage disorders caused by mutations in 13 distinct *CLN* genes^1^. Despite this diversity of genetic causes, most forms share a hallmark pathology: impaired lysosomal function leading to the accumulation of undegraded lipids and proteins as lipofuscin^2^. This build-up drives neuroinflammation and neurodegeneration, with symptoms typically emerging in late infancy and progressing to premature death early in life^2^. The precise molecular roles of many CLN proteins in lysosomal homeostasis, however, are poorly defined, although recent studies have implicated several CLN proteins in the synthesis of bis(monoacylglycero)phosphate (BMP), a signature lysosomal phospholipid^3–6^.

BMP is found exclusively in late endosomes and lysosomes, where it plays a key role in maintaining lysosomal membrane dynamics and catabolic capacity^7^. Unlike other animal phospholipids, which contain a glycerol backbone with an *R*-configured chiral centre, BMP consists of two glycerol molecules linked to a phosphate group, each containing a chiral carbon in the *S* configuration (*S*,*S*-BMP)^8–10^. Cellular BMP is derived from phosphatidylglycerol (PG), which features an *R*-configured backbone and an *S*-configured glycerol headgroup (*R*,*S*-PG)^11–15^. The pathway converting *R*,*S*-PG into *S*,*S*-BMP has yet to be defined.

Two Batten disease proteins, CLN5 and CLN8, have recently been implicated in distinct acyl transfer steps within BMP biosynthesis^3,4^. CLN5 functions as a lysosomal transacylase, transferring an acyl group from one lysophosphatidylglycerol (LPG) molecule to the headgroup of another to complete the final step of BMP synthesis^4^. We previously identified CLN8, a member of the TRAM-LAG1-CLN8 (TLCD) acyltransferase family, as essential for cellular BMP production^3^. However, the precise acyl transfer reaction catalysed by CLN8, its molecular mechanism and its position within the BMP biosynthetic pathway have remained unknown.

## Results

### CLN8 acylates *S*,*S*-GPG

**Fig. 1:**
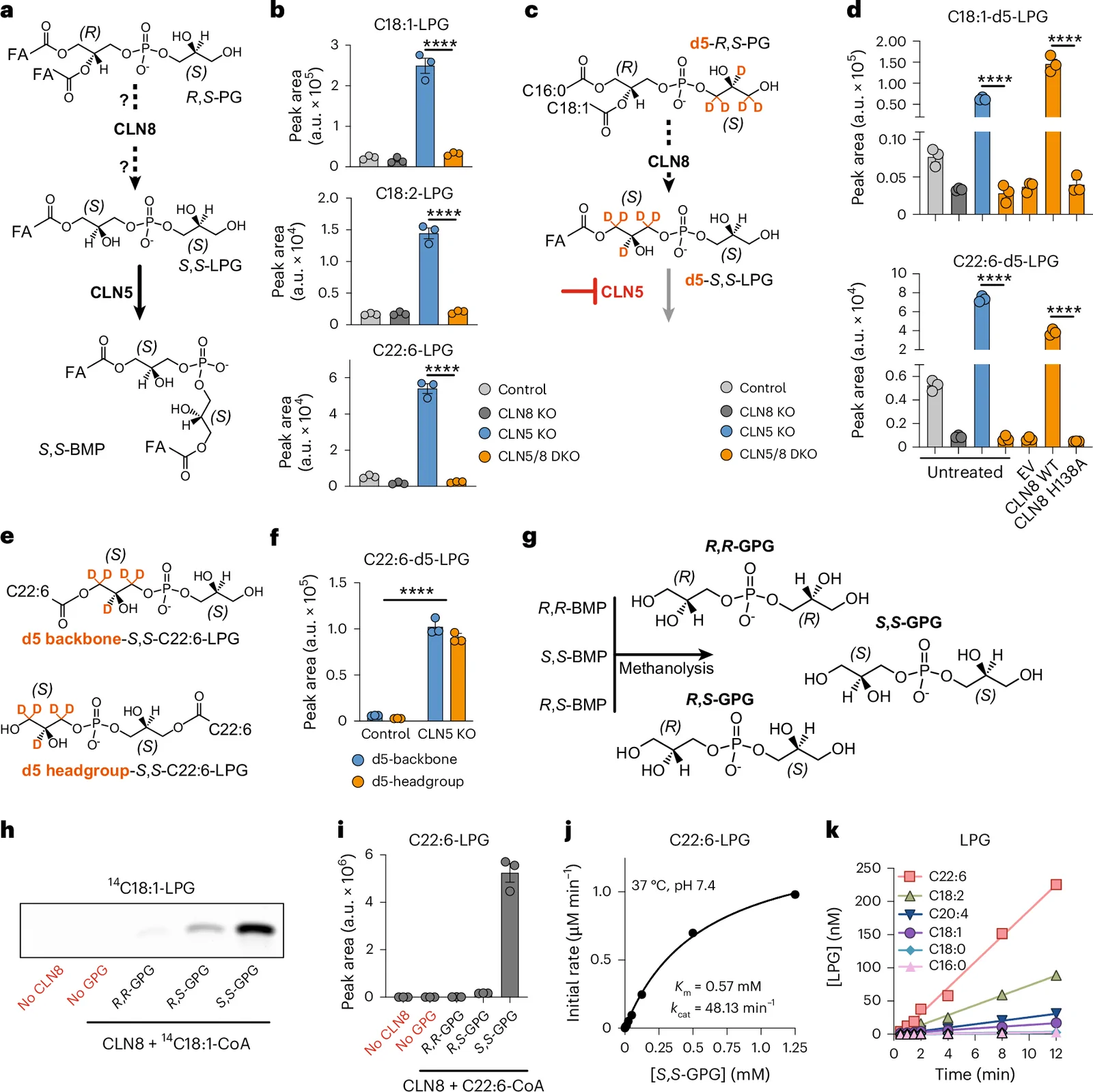
CLN8 is a stereospecific *S*,*S*-GPG acyltransferase in the BMP synthesis pathway. **a**, BMP biosynthesis pathway schematic. *R*,*S*-PG is converted to *S*,*S*-LPG through several steps, one of which is catalysed by CLN8. *S*,*S*-LPG then serves as both acyl donor and acceptor for CLN5 to generate *S*,*S*-BMP. **b**, The levels of the indicated LPG species in control, CLN8-KO, CLN5-KO and CLN5/8-DKO HeLa cells under standard culture conditions (*n* = 3 biological replicates per genotype). **c**, Schematic of the labelling experiment to trace the synthesis of *S*,*S*-LPG. **d**, Levels of d5-labelled LPG species in the indicated cell lines, either untreated or transfected for 48 h with CLN8 WT or the catalytically inactive H138A mutant, then treated with 25 μM d5-PG for 6 h (*n* = 3 biological replicates). **e**, Structures of *S*,*S*-C22:6-LPG species containing the d5 label either in the glycerol headgroup or backbone. **f**, Abundance of C22:6-LPG species containing the d5 label in the headgroup or backbone in control and CLN5-KO cells following 6 h labelling with 25 μM d5-PG (*n* = 3 biological replicates per genotype). **g**, Schematic of the generation of *R*,*R*-, *R*,*S*- and *S*,*S*-GPG substrates. **h**, Phosphorimaging of TLC-separated LPG band from in vitro assays with purified CLN8, ^14^C18:1-CoA and different GPG stereoisomers. **i**, Abundance of C22:6-LPG product from in vitro assays using purified CLN8, C22:6-CoA and different GPG stereoisomers (*n* = 3 assay replicates). **j**, Michaelis–Menten kinetics of purified CLN8 activity using saturating C22:6-CoA and increasing concentrations of *S*,*S*-GPG. **k**, Time-dependent production of indicated LPG species in reactions with purified CLN8, equimolar acyl-CoA mix and *S*,*S*-GPG. All data shown in graphs are presented as mean ± s.e.m. In vitro assay results are representative of at least two independent protein preparations. ****P < 0.0001 by one-way ANOVA with Dunnett’s post hoc test (**b** and **d**) or by two-way ANOVA (**f**). Source data

To define the enzymatic role of CLN8 in BMP biosynthesis, we investigated its function in the context of CLN5. CLN5 acts as a lysosomal transacylase, and CLN5-deficient cells accumulate lysosomal LPG while lacking *S*,*S*-BMP^4,16^. We reasoned that because transacylation does not alter the glycerol backbone, the CLN5 substrate LPG must already have the *S*,*S* stereoconfiguration, placing CLN8 upstream in the conversion of *R*,*S*-PG to *S*,*S*-LPG (Fig. 1a).

**Extended Data Fig. 1:**
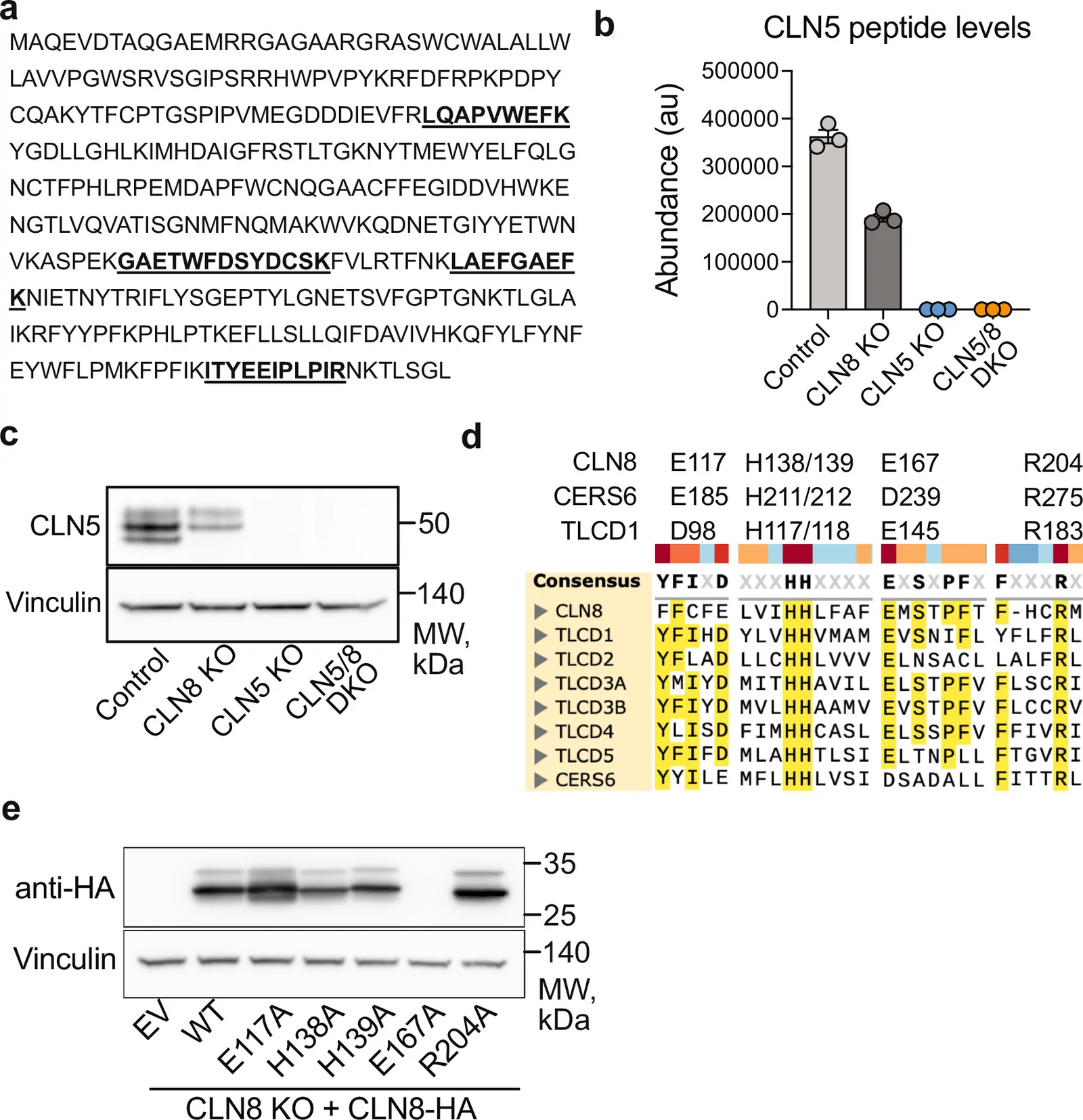
Validation of CLN5 and CLN8 HeLa cell models and overexpression constructs. **a**, CLN5 tryptic peptides used for protein quantification, highlighted within the CLN5 amino acid sequence. **b**, The abundance of CLN5 peptides in HeLa control, CLN8 KO, CLN5 KO, and CLN5/8 double KO (DKO) cell lines (n = 3 biological replicates). **c**, Immunoblot showing CLN5 protein levels and vinculin loading control in the same cell lines as in (**b**). **d**, Protein sequence alignment of selected TLCD family members, showing regions surrounding conserved residues only; residue numbers for CLN8, CERS6, and TLCD1 are indicated above. **e**, Expression of the indicated CLN8-HA mutant constructs in CLN8 KO cells. The blot is representative of two independent cell culture experiments. All data shown in graphs are presented as mean ± s.e.m. Source numerical data and unprocessed blots are available in source data. Source data

To test this hypothesis, we utilized our previously published CLN8-knockout (KO) HeLa cells, which lack BMP species^3^, and generated CLN5/8-double-KO (DKO) cells by introducing a CLN5 deletion; in parallel, we engineered CLN5 single-KO cells (Extended Data Fig. 1a–c). Comparison of LPG levels across these lines showed that CLN5-KO cells accumulated multiple LPG species, consistent with a block in the final pathway step converting LPG to BMP (Fig. 1b). This accumulation was lost in CLN5/8-DKO cells, demonstrating that CLN8 is required for LPG synthesis upstream of CLN5 (Fig. 1b).

To trace the conversion of *R*,*S*-PG to *S*,*S*-LPG directly, we supplemented cells with *R*,*rac-*PG containing a deuterium-labelled racemic glycerol headgroup and an *R*-configured diacylglycerol (C16:0/C18:1) backbone (d5-PG) (Fig. 1c). In CLN5-KO cells, d5-LPG species containing oleate (C18:1) or docosahexaenoate (DHA, C22:6) accumulated substantially, as was observed for unlabelled LPG species (Fig. 1b,d). This accumulation was lost in CLN5/8-DKO cells and restored by hemagglutinin (HA)-tagged wild-type (WT) CLN8 (CLN8-HA) but not by the catalytically dead CLN8 H138A mutant lacking the conserved TLCD histidine required for acyl transfer^3^ (Fig. 1d and Extended Data Fig. 1d). Thus, CLN8 acyltransferase activity is essential for *R*,*S*-PG-to-LPG conversion in cells.

To define the CLN8 reaction further, we analysed C22:6-d5-LPG, which accumulates in CLN5-KO cells but is depleted in CLN8-KO and CLN5/8-DKO cells (Fig. 1d). This lipid provides a specific readout of cellular CLN8 activity, as it can only arise through complete removal of the original 16:0 and 18:1 acyl chains from d5-PG, followed by CLN8-mediated C22:6-CoA acylation (where CoA is coenzyme A). Supplementation with d5-PG could, in principle, yield two forms of C22:6-d5-LPG: backbone labelled and headgroup labelled (Fig. 1e), which are distinguishable by their daughter ions in tandem mass spectrometry (MS; Methods). Surprisingly, tandem MS analysis revealed equal accumulation of both backbone- and headgroup-labelled C22:6-d5-LPG in CLN5-KO cells (Fig. 1f). This finding supports only a model in which CLN8 acylates a symmetrical substrate with two equivalent glycerol moieties—namely, glycerophosphoglycerol (GPG).

**Extended Data Fig. 2:**
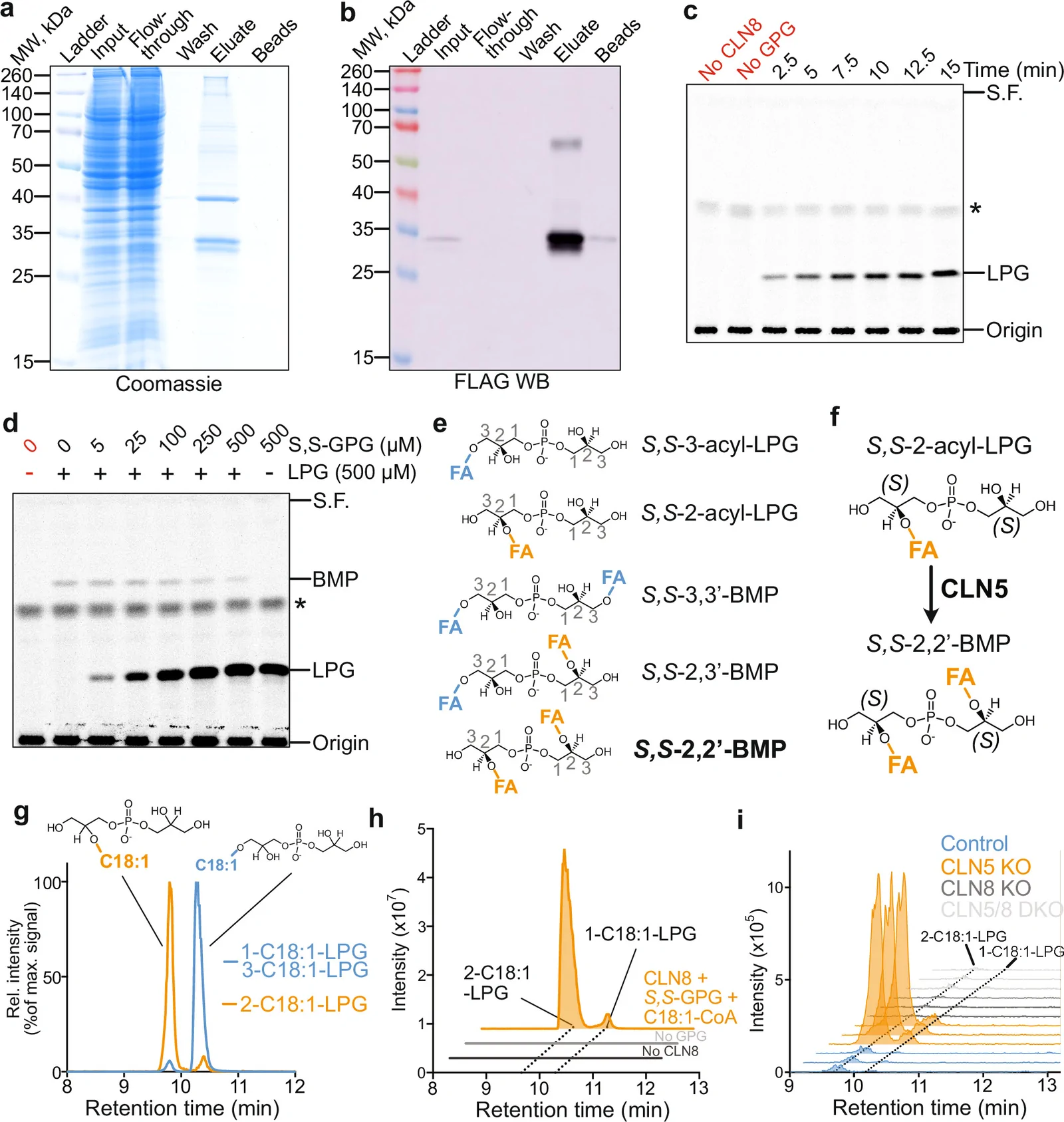
Purification and enzymatic characterization of CLN8 expressed in human cells. **a**, Coomassie-stained SDS–PAGE showing total protein in the indicated fractions collected during purification of FLAG–STREP-tagged CLN8. **b**, Western blot detecting the FLAG tag in the same fractions as in (**a**). The eluate, passed through size-exclusion spin columns to remove biotin, was used directly for in vitro assays. The blot is displayed as a chemiluminescence–colourimetric overlay. **c**, Phosphorimage of a TLC plate showing a time-course assay with purified CLN8 incubated with oleoyl-CoA containing a [1-^14^C]-oleoyl-CoA tracer and *S,S*-GPG substrate. **d**, Phosphorimage of a TLC plate from a substrate-competition assay using purified CLN8 and [1-^14^C]-oleoyl-CoA, with *R,rac*-LPG fixed at 500 µM and *S,S*-GPG titrated at the indicated concentrations. In (**c,d**), migration positions of LPG and BMP were validated using chemical standards; the asterisk denotes a non-specific band from the [1-^14^C]-oleoyl-CoA preparation. **e**, Structures of the possible acyl positional isomers of *S,S*-LPG and *S,S*-BMP, with acyl positions indicated by numbers adjacent to each carbon. Because of the *S* stereochemistry of the glycerol backbone, the phosphate-linked glycerol carbon is assigned as *sn*-1, and the distal glycerol carbon as *sn*-3. The physiological 2,2’-BMP isoform is highlighted in bold. **f**, Schematic illustrating the *sn-2* acyl positions of the LPG precursor and BMP product. This assignment is based on previous reports of BMP acyl chain positions, as BMP positional isomers are not resolved by our LC-MS/MS method. **g**, LC-MS/MS chromatograms showing the migration of 1-C18:1-LPG, 3-C18:1-LPG and 2-C18:1-LPG standards. Note that 1-C18:1-LPG and 3-C18:1-LPG are stereoisomers rather than acyl positional isomers, because glycerol *sn* nomenclature is determined by the chirality of the central carbon. **h**, LC-MS/MS chromatogram of C18:1-LPG generated in vitro from purified CLN8, *S,S*-GPG, and C18:1-CoA, compared against positional isomer standards. **i**, LC–MS/MS chromatograms of C18:1-LPG detected in control, CLN8 KO, CLN5 KO, and CLN5/8 DKO HeLa cells (n = 3 per genotype), resolved against positional isomer standards. In vitro assay results (**c, d**) are representative of two independent CLN8 purifications. Source numerical data and unprocessed gels and blots are available in source data. Source data

Although acylation of glycerophosphodiesters (GPDs) has previously been described only in yeast^17,18^, and GPDs in vertebrates are thought to be destined for degradation^19^, our data suggested GPG is a bona fide CLN8 substrate. To test this, we generated all three potential GPG stereoisomers (*S*,*S*, *R*,*S* and *R*,*R*) by deacylating synthetic BMP standards and validated them by liquid chromatography–tandem MS (LC–MS/MS) against a chemically synthesized *R*,*S*-GPG standard (Fig. 1g). In in vitro assays with purified CLN8 (Extended Data Fig. 2a,b) and ^14^C18:1-CoA, CLN8 acylated *S*,*S*-GPG most efficiently, with substantially lower activity for *R*,*S*-GPG and only trace activity with *R*,*R*-GPG (Fig. 1h). Similar stereospecificity was observed using unlabelled C22:6-CoA, with robust C22:6-*S*,*S*-LPG production only from *S*,*S*-GPG (Fig. 1i). CLN8 acylated *S*,*S*-GPG with C18:1-CoA and C22:6-CoA in a time- and concentration-dependent manner, with substantially higher capacity than what we had previously observed for *R*,*rac*-LPG acyl acceptor substrate^3^ (Fig. 1j and Extended Data Fig. 2c,d). Kinetic analysis showed CLN8’s affinity and catalytic efficiency for *S*,*S*-GPG were comparable to those of other acyltransferases acting on physiological substrates^3,17^ (Fig. 1j). CLN8 also displayed a marked preference for polyunsaturated acyl-CoA substrates, particularly C22:6-CoA and C18:2-CoA (Fig. 1k).

Prior studies report that lysosomal BMPs contain fatty acids esterified to the *sn*-2 and *sn*′-2 positions of the glycerol moieties^20^ (Extended Data Fig. 2e). This configuration suggests that CLN8 should generate *S*,*S*-2-acyl-LPG intermediates, positioning the acyl chain correctly for CLN5 to condense them into 2,2′-BMP (Extended Data Fig. 2f). To determine whether CLN8 acylates the *sn*-2 or *sn*-3 position of the glycerol moiety in *S*,*S*-GPG, we analysed 1-C18:1- and 2-C18:1-LPG standards, which are readily resolved by LC–MS/MS (Extended Data Fig. 2g). When *S*,*S*-GPG was incubated with purified CLN8 and C18:1-CoA, the reaction product co-eluted with 2-C18:1-LPG standard (Extended Data Fig. 2h). Consistently, the C18:1-LPG species that accumulates in CLN5-deficient cells was also identified as 2-C18:1-LPG (Extended Data Fig. 2i). Together, our stable-isotope tracing and enzymatic assays identify CLN8 as a stereospecific GPG acyltransferase that converts *S*,*S*-GPG and acyl-CoAs into *S*,*S*-2-acyl-LPG. This product is then utilized by CLN5 in the lysosome to form BMP.

### Structure and reaction mechanism of CLN8

**Fig. 2:**
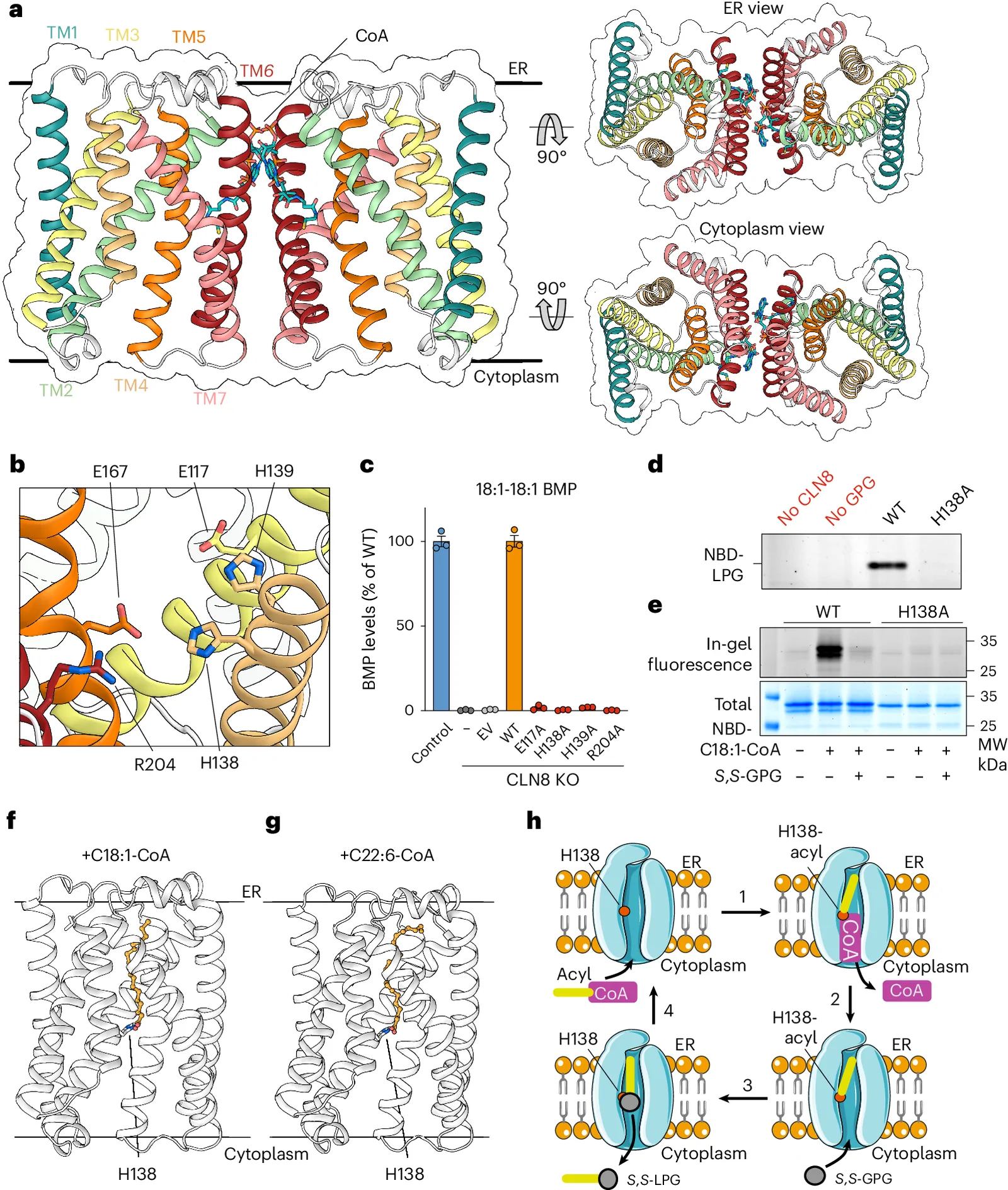
Structure and reaction mechanism of CLN8 homodimer. **a**, Cartoon representation of the CLN8 homodimer, shown in side view and in views from the ER lumen and cytoplasm, with bound CoA molecules in cyan. **b**, Active site of CLN8 highlighting catalytic residues. **c**, Cellular levels of 18:1–18:1 BMP in control and CLN8-KO HeLa cells transfected with CLN8 WT or alanine mutants of active-site residues (*n* = 3 biological replicates). **d**, Fluorescence scan of thin-layer chromatography showing LPG formation in in vitro assays with NBD-18:1-CoA, purified CLN8 WT or H138A and *S*,*S*-GPG. **e**, In-gel NBD fluorescence (top) and Coomassie staining (bottom) of SDS–PAGE of purified CLN8 WT and H138A incubated with NBD-18:1-CoA alone or together with *S*,*S*-GPG, as indicated. **f**,**g**, CLN8 cartoon structures solved with C18:1-CoA and C22:6-CoA, showing bound fatty acyl groups and H138 residues. Only one monomer of the dimer is shown. **h**, Catalytic cycle of CLN8, shown for clarity using only one monomer of the dimer. All data shown in graphs are presented as mean ± s.e.m. In vitro assay results (**d** and **e**) are representative of two independent protein purifications. Icons in **h** adapted from Servier Medical Art via Bioicons under a Creative Commons license CC by 3.0. Source data

**Extended Data Fig. 3:**
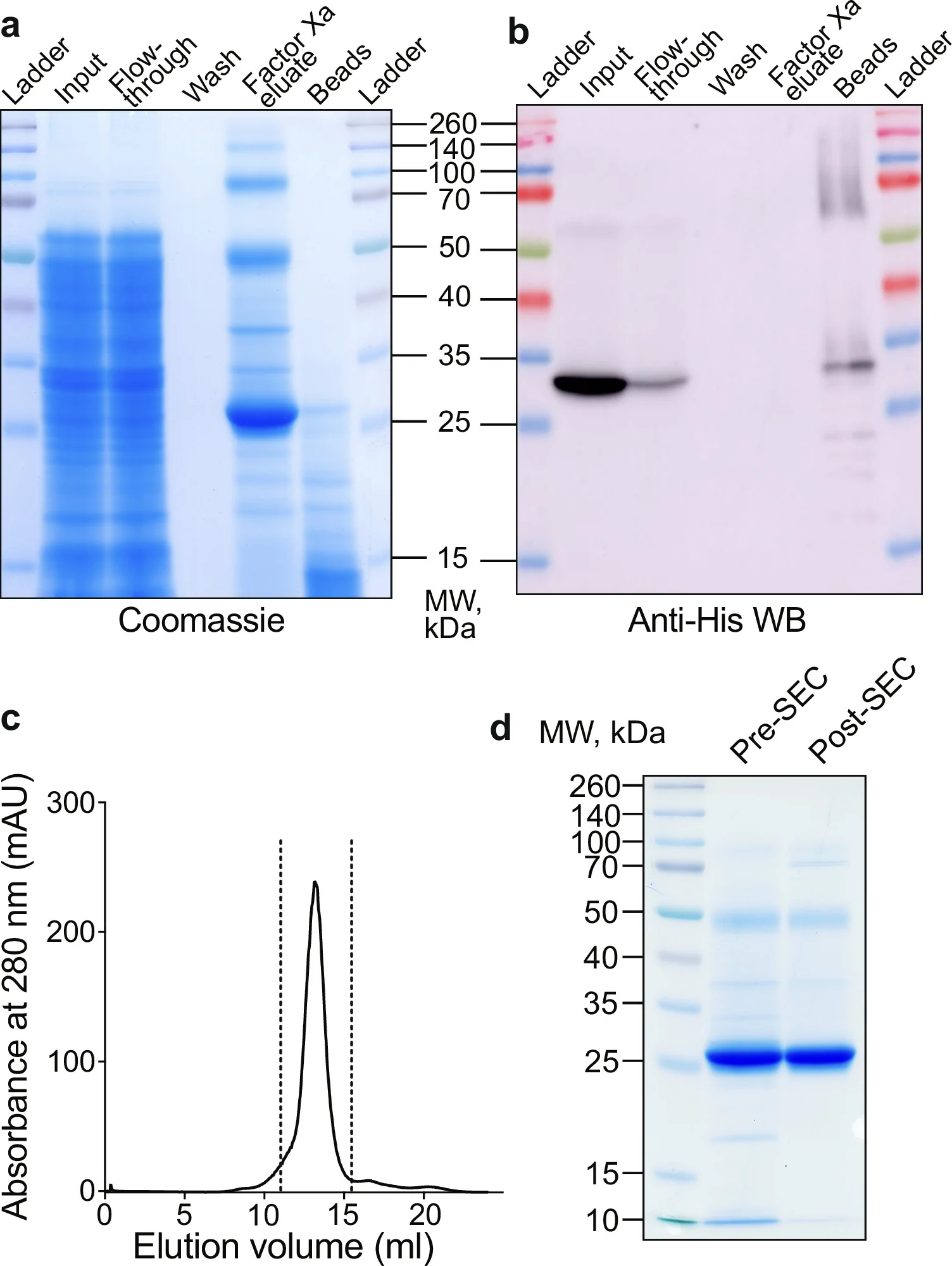
Purification of CLN8 from yeast for structural studies. **a**, Coomassie-stained SDS–PAGE showing total protein in the indicated fractions during purification of 6×His-tagged, codon-optimised CLN8, containing a Factor Xa protease cleavage site between the tag and coding sequence. **b**, Western blot detecting the 6×His tag in the same fractions as in (**a**). **c**, Size-exclusion chromatography (SEC) of the eluate from (**a**), showing a single homogeneous protein population. Dashed lines indicate the fractions collected for cryo-EM. **d**, Coomassie-stained SDS–PAGE comparing eluate before and after size-exclusion chromatography, with the post-SEC sample used for cryo-EM grid preparation. Protein purification gels and blots in **a**, **b** and **d** are representative of five independent CLN8 purifications. Source numerical data and unprocessed gels and blots are available in source data. Source data

**Extended Data Fig. 4:**
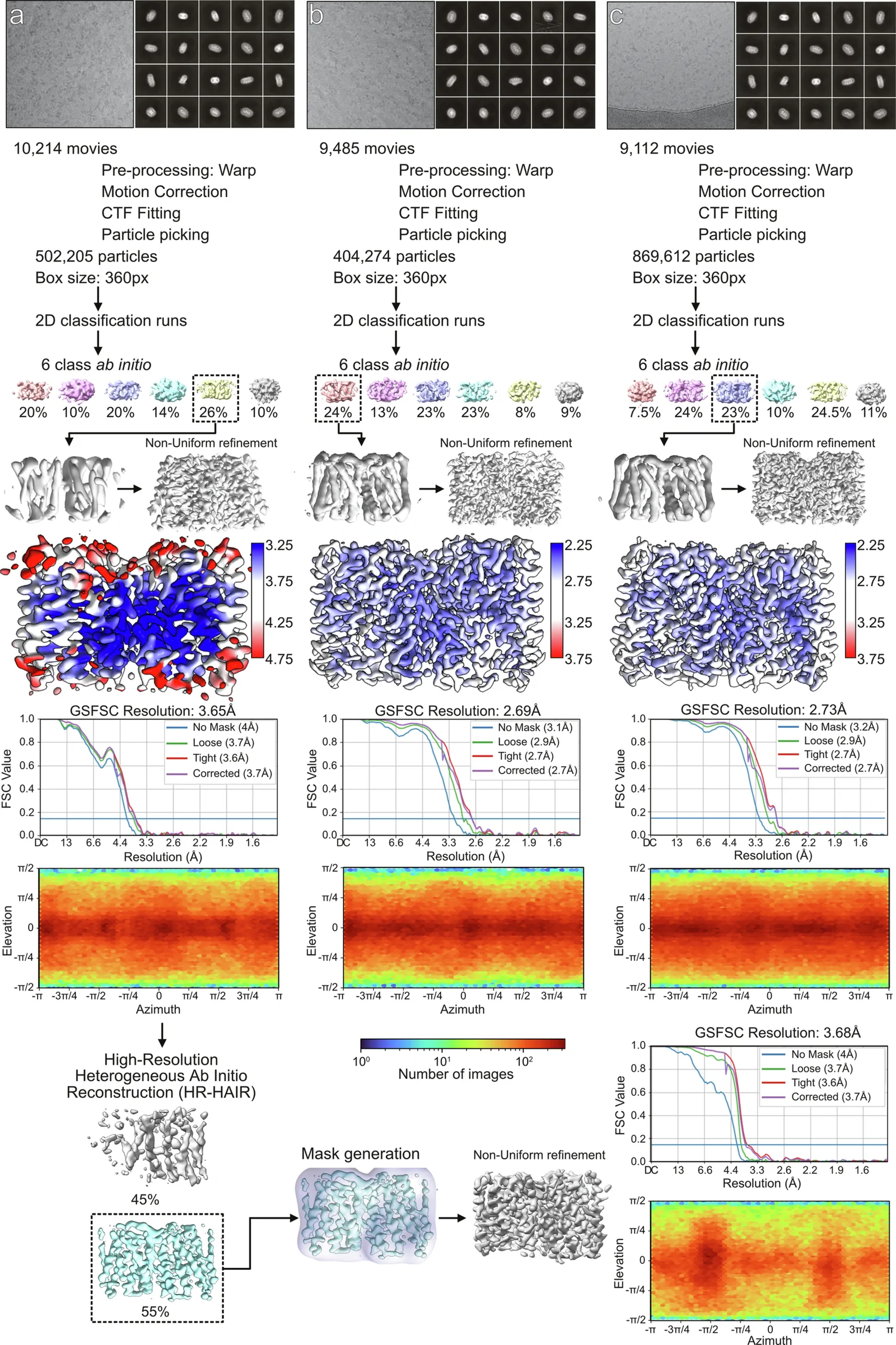
Cryo-EM data processing for CLN8 apo and substrate-bound structures. Representative micrographs, 2D class averages generated before 3D classification, data-processing workflows, final 3D reconstructions coloured by local resolution, Fourier shell correlation (FSC) curves indicating overall map resolution, and angular-distribution plots of the particles contributing to the final reconstructions are shown for: **a**, apo CLN8; **b**, CLN8 incubated with 100 µM C18:1-CoA for 1 h at room temperature; and **c**, CLN8 incubated with 100 µM C22:6-CoA for 1 h at room temperature.

**Extended Data Fig. 5:**
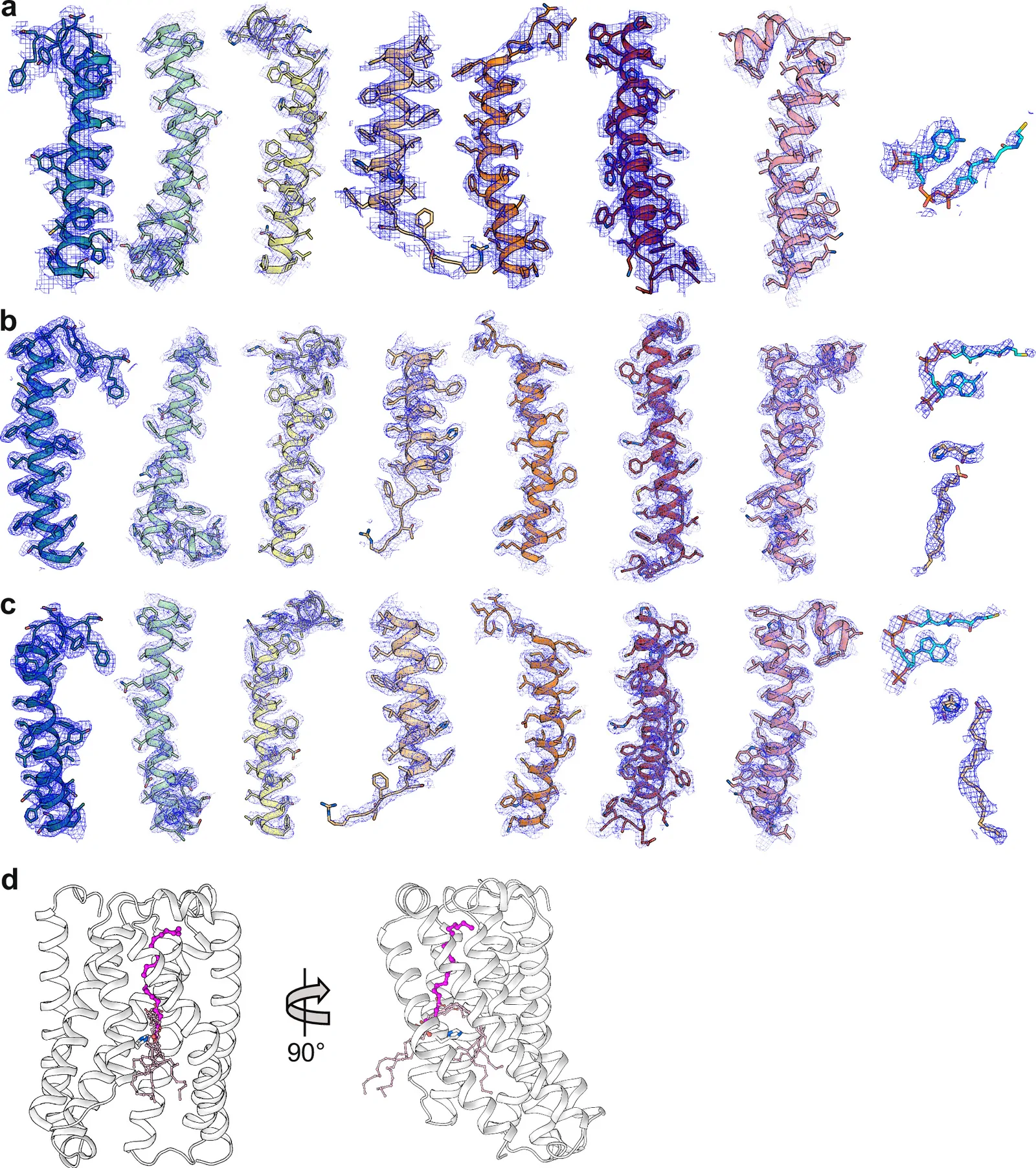
Cryo-EM map quality validation for CLN8 apo and substrate-bound structures. Representative cryo-EM densities (black mesh) for transmembrane helices and bound CoA, with side chains shown as sticks: **a**, apo CLN8; **b**, CLN8 solved with C18:1–CoA; **c**, CLN8 solved with C22:6–CoA. Maps are contoured in Chimera at 0.11 (0.56σ), 0.246 (1.68σ) and 0.22 (1.73σ), respectively, and are displayed within 2 Å of the depicted features. For (**b**,**c**), the positions of the fatty acyl chains adjacent to H138 are indicated. Note the absence of continuous density between the fatty acyl carboxyl groups and H138. **d**, Cartoon representation of the CLN8 structure solved with C22:6-CoA, showing the main observed density for C22:6 (red) and minor densities corresponding to alternative binding poses (yellow).

**Extended Data Fig. 6:**
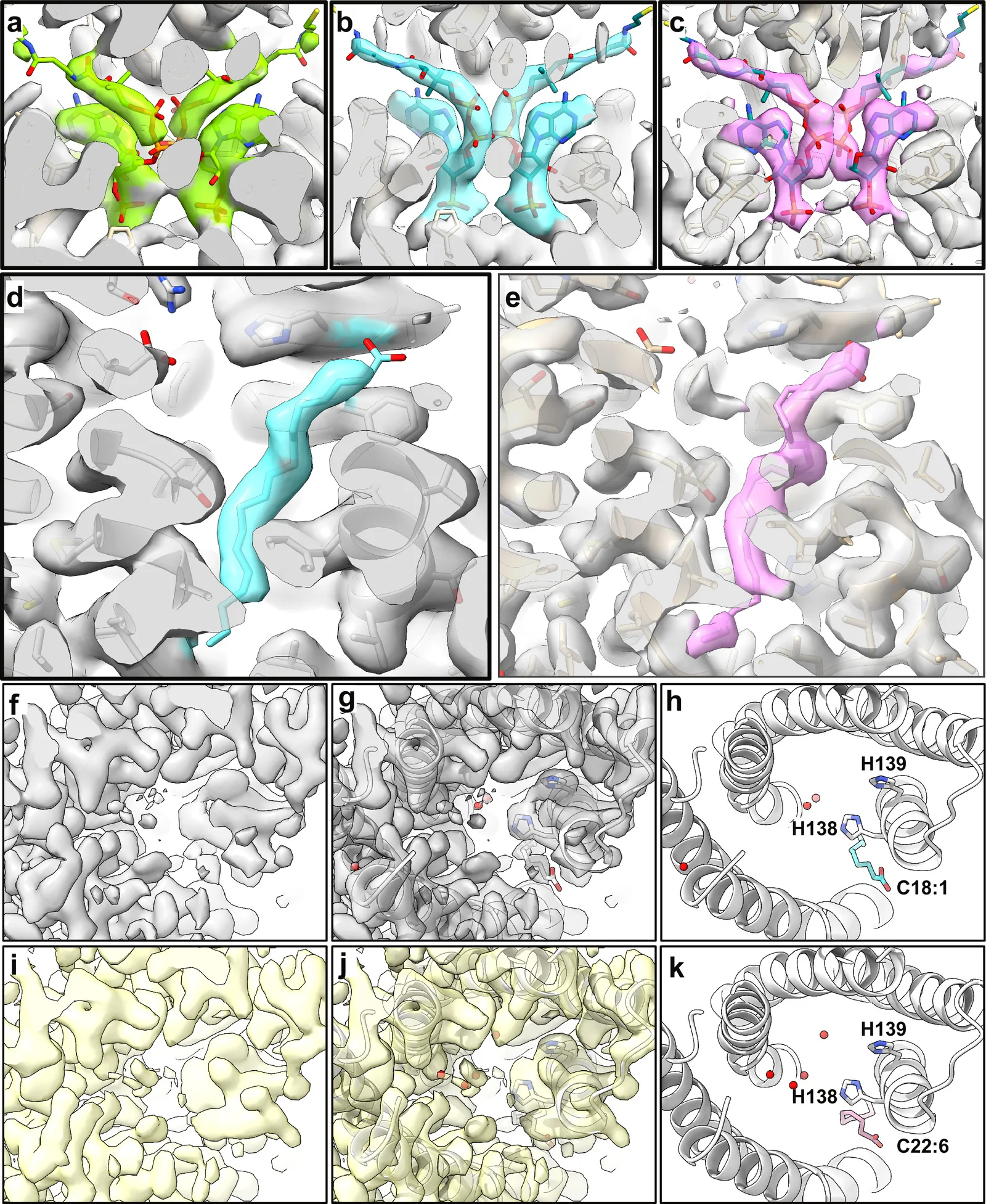
Cryo-EM density of CoA, bound acyl chains and ordered water molecules in the CLN8 active site. **a**-**c**, Cryo-EM density corresponding to CoA in the apo (**a**), C18:1-CoA-bound (**b**) and C22:6-CoA-bound (**c**) structures, shown at comparable contour levels of 8.0 σ, 11.4 σ and 9.0 σ, respectively, illustrating map quality and agreement with the fitted model. **d**,**e**, Cryo-EM density of the bound acyl chains in the C18:1-bound (**d**) and C22:6-bound (**e**) structures. In **a**-**e**, protein density is shown in grey, ligands are coloured cyan for the C18:1-bound structure and light pink for the C22:6-bound structure, and cryo-EM maps are displayed as semi-transparent surfaces. **f**-**k**, Close-up views of the catalytic site centred on H138 and H139, showing ordered water molecules modelled within the active-site cavity. Cryo-EM density alone is shown for the C18:1-bound (**f**; contoured at 10.7 σ) and C22:6-bound (**i**; contoured at 10.9 σ) structures. Overlay of density and model is shown for the C18:1-bound (**g**) and C22:6-bound (**j**) structures. The model alone, with labelled active-site residues and bound acyl chain, is shown for the C18:1-bound (**h**) and C22:6-bound (**k**) structures. Water molecules are shown as red spheres.

To dissect the biochemical mechanism of CLN8, we purified tag-cleaved, codon-optimized human CLN8 from yeast for structural studies (Extended Data Fig. 3a,b). Similar to other TLCD family members, purified CLN8 formed SDS-resistant multimers on SDS–polyacrylamide gel electrophoresis (PAGE), but size-exclusion chromatography (SEC) revealed a single homogeneous population^3^ (Extended Data Fig. 3c,d). The SEC eluate was concentrated, confirmed to be enzymatically active and used for cryo-electron microscopy (cryo-EM). Two-dimensional class averages revealed a dimeric architecture resembling its paralogue ceramide synthase 6 (CERS6)^21^ (Extended Data Fig. 4a). The initial structure, resolved at 3.65 Å (Supplementary Table 1), showed well-defined transmembrane domains, whereas the endoplasmic reticulum (ER)-luminal N terminus (residues 1–15) and cytosolic C terminus (residues 261–286) were disordered and not resolved^22^ (Extended Data Figs. 4a and 5a). High-resolution heterogeneous ab initio reconstruction (HR-HAIR)^23^ of the apo dataset revealed conformational heterogeneity, and the final apo structure was determined from the dominant particle class, in which TM4 is well defined; the remaining particles could not be refined into a coherent map, consistent with conformational flexibility in this region in the absence of substrate (Extended Data Fig. 4a). CLN8 forms a homodimer, with each monomer containing seven transmembrane helices and an interface mediated by TM6 (Fig. 2a). Unexpectedly, we observed two CoA molecules bound in pockets located within the transmembrane region of each monomer, positioned at the dimer interface (Fig. 2a and Extended Data Figs. 5a and 6a). Given its polarity, CoA incorporation within the membrane probably occurs during dimer assembly. Although only the CoA moiety is resolved in the cryo-EM density, this species may represent acyl-CoA, with the acyl chain remaining unresolved because of conformational flexibility outside the ordered protein core.

The TLC domain (TM2–7) forms a barrel enclosing a central cavity open to the cytosol and sealed towards the ER lumen by the TM2–TM3 and TM4-TM5 loops (Fig. 2a). At the centre of this cavity, we identified a putative active site comprising E117, H138, H139, E167 and R204, which is conserved across the TLCD acyltransferases^3^ (Fig. 2b and Extended Data Fig. 1d). Alanine substitutions of these residues (except E167A, which was not expressed) failed to restore BMP levels in CLN8-KO HeLa cells despite normal protein expression levels (Fig. 2c and Extended Data Fig. 1e), confirming their essentiality for CLN8 catalysis.

Given that the conserved histidines H211 in CERS6 and H117 in TLCD1 form covalent acyl-enzyme intermediates during ceramide and phosphatidylethanolamine synthesis^3,21^, respectively, we tested whether the analogous residue H138 in CLN8 serves the same role (Extended Data Fig. 1d). WT CLN8, but not H138A, purified from human cells could utilize fluorescent NBD-C18:1-CoA as an acyl donor to catalyse NBD-C18:1-*S*,*S*-LPG synthesis from *S*,*S*-GPG (Fig. 2d). Consistent with formation of a catalytic intermediate, SDS–PAGE revealed a covalent fluorescent NBD-C18:1-CLN8 adduct that was lost on the addition of *S*,*S*-GPG, whereas no such intermediate was detected for H138A (Fig. 2e).

To further visualize this intermediate state, we determined cryo-EM structures of CLN8 prepared in the presence of either C18:1-CoA or C22:6-CoA at 2.7 Å resolution (Fig. 2f,g, Extended Data Fig. 4b,c and Supplementary Table 1). The improved resolution relative to the apo structure probably reflects stabilization by the bound acyl chain, enabling refinement of the model (Fig. 2a and Extended Data Figs. 4 and 5b,c). In both complexes, the acyl chain was positioned with a carboxy group adjacent to H138 within the central cavity and the tail oriented towards the ER lumen (Fig. 2f,g and Extended Data Fig. 6d,e). CoA molecules were also observed between the two monomers in the same configuration as in the apo structure (Extended Data Figs. 5b,c and 6a–c). In the C18:1-bound complex, we observed a single, well-defined acyl chain density, consistent with the more restricted conformational flexibility of the monounsaturated chain. By contrast, the C22:6-bound complex showed weaker, less continuous density with additional minor densities, probably reflecting the greater conformational flexibility of the polyunsaturated chain and averaging of multiple binding poses during reconstruction (Extended Data Fig. 5d). The absence of CoA-SH from the active site, together with the positioning of the acyl chain adjacent to H138, is consistent with a substrate-bound state in which the acyl group remains poised for transfer to *S*,*S*-GPG entering from the cytosolic side (Fig. 2f,g).

Although the structures capture an acyl chain positioned next to H138, continuous covalent density between H138 and the acyl chain was not resolved (Extended Data Figs. 5b,c and 6d,e). This may reflect the hydrolytic lability of an *N*-acyl-histidine intermediate under aqueous cryo-EM sample preparation conditions. Consistent with this possibility, cavity analysis revealed that the solvent-accessible volume surrounding H138 in CLN8 is one to two orders of magnitude larger than the equivalent cavities in other acyltransferase enzymes with closely related structures, including CERS6 (Supplementary Table 2). This solvent-exposed architecture of the CLN8 active site is consistent with the highly hydrophilic nature of the acyl acceptor *S*,*S*-GPG and contrasts with related acyltransferases in which the acyl intermediate is stabilized within a more protected hydrophobic environment (Supplementary Table 2). In agreement with this analysis, ordered water molecules could be successfully modelled within the active site cavity in both the C18:1-CoA and C22:6-CoA cryo-EM maps (Extended Data Fig. 6f–k). This solvent exposure could render an acyl-imidazole intermediate kinetically labile in solution, accounting for the absence of continuous covalent density in the cryo-EM maps while remaining consistent with the H138-dependent NBD-C18:1-CLN8 covalent adduct observed by in-gel fluorescence (Fig. 2d,e).

Further supporting this catalytic model, the addition of *S*,*S*-GPG to CLN8 preincubated with C22:6-CoA yielded only the apo state in cryo-EM, consistent with acyl transfer and *S*,*S*-LPG product release. Together, these observations indicate that CLN8 uses sequential substrate binding and supports a ping-pong acyl transfer mechanism, with H138 playing a key catalytic role analogous to the active-site histidines of related TLCD acyltransferases^3,21^ (Fig. 2h).

### Functional impact of Batten disease-causing CLN8 mutations

**Fig. 3:**
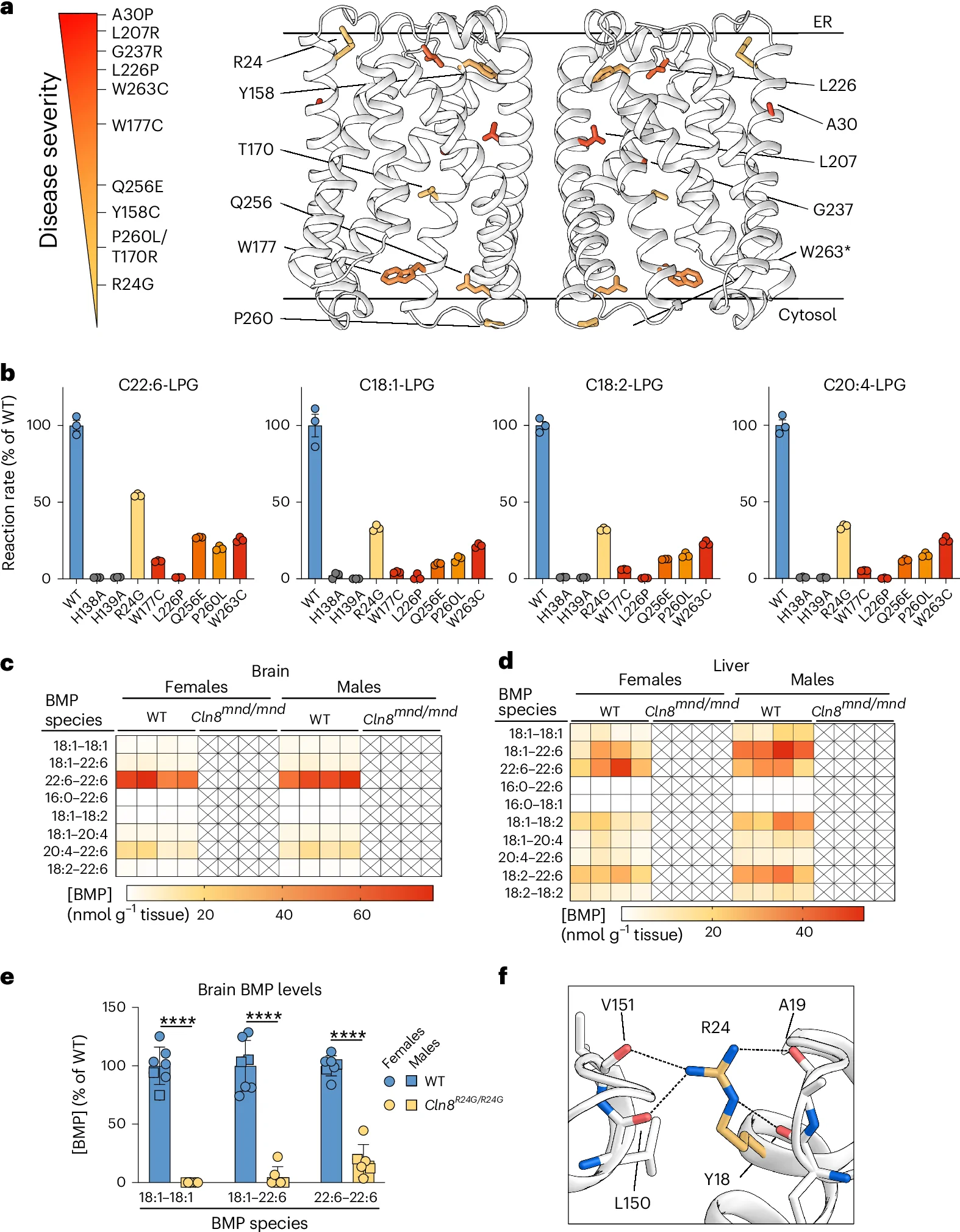
Impact of CLN8 mutations on enzymatic activity. **a**, Cartoon representation of the CLN8 homodimer with selected Batten disease missense mutations highlighted. Mutations are coloured by relative disease severity, classified on the basis of reported age of onset and clinical symptoms. P260L and T170R were reported as compound heterozygous variants. W263 lies within the disordered C-terminal region not resolved in the structure. **b**, Residual activity of purified mutant CLN8 variants with the indicated acyl-CoA substrates, expressed relative to purified CLN8 WT, and coloured according to relative disease severity (*n* = 3 assay replicates). **c**,**d**, Levels of the indicated BMP species in brains (**c**) and livers (**d**) of WT or *Cln8*^*mnd/mnd*^ (*Cln8* frameshift mutation) 6-month-old mice (*n* = 4 WT and 4 homozygous males and females). No BMP species were detected in *Cln8*^*mnd/mnd*^ tissues. **e**, Levels of the indicated BMP species in brains from 8-week-old WT and *Cln8*^*R24G/R24G*^ homozygous knock-in mice, expressed as a percentage of the WT mean (*n* = 2 WT males and 2 homozygous males; 5 WT females and 5 homozygous females). **f**, Cartoon model showing electrostatic interactions between residue R24 and backbone carbonyl groups of neighbouring residues. All data shown in graphs are presented as mean ± s.e.m. In vitro assays are representative of at least two independent protein preparations. In **e**, ****P < 0.0001 by unpaired two-tailed Student’s *t*-test. Source data

**Extended Data Fig. 7:**
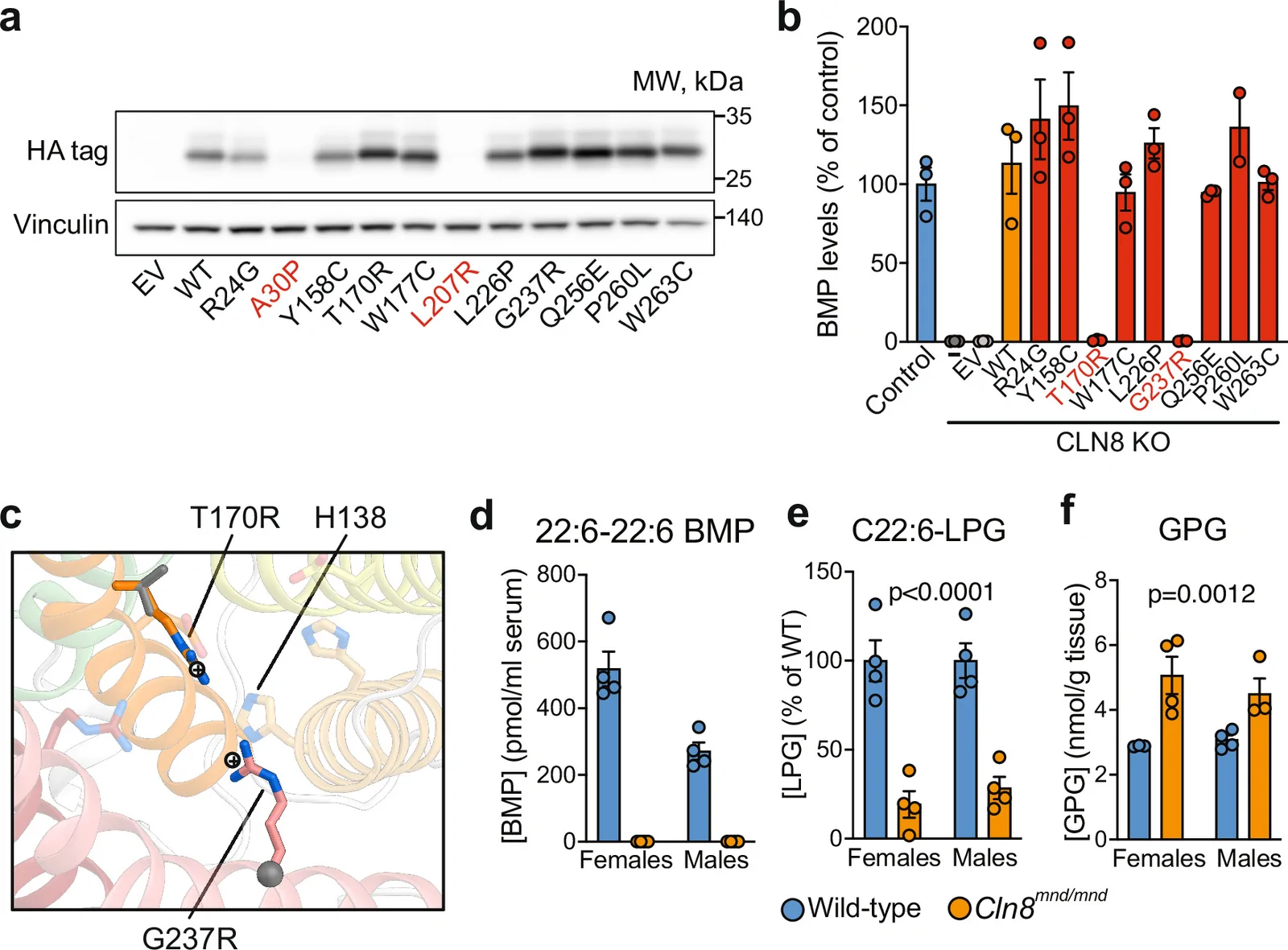
Expression and functional impact of CLN8 mutations. **a**, Western blot analysis of HA-tagged CLN8 constructs expressed in CLN8 KO HeLa cells, detected with anti-HA antibody, with vinculin as a loading control (48 h post-transfection). **b**, Total BMP levels measured in control or CLN8 KO HeLa cells transfected with the indicated CLN8-HA constructs (n = 3 biological replicates, 48 h). **c**, Structural overlay showing the positions of T170R and G237R mutations within the CLN8 active site. The side chains of the mutant arginines are positioned 2.7 Å and 4.3 Å from the catalytic residue H138, respectively. **d**, Levels of 22:6–22:6-BMP in the serum of 6-month-old *Cln8*^*mnd/mnd*^ mice (n = 4 per sex). **e**, Levels of C22:6-LPG, and (**f**) GPG in the brains of 6-month-old wild-type and *Cln8*^*mnd/mnd*^ mice (n = 3 wild-type female, 4 wild-type male, 4 *Cln8*^*mnd/mnd*^ female and 3 *Cln8*^*mnd/mnd*^ male). All data shown in graphs are presented as mean ± s.e.m. In **(**e,f), indicated *P* values reflect genotype effects in two-way ANOVA; the sex factor was not significant. Source numerical data and unprocessed blots are available in source data. Source data

Next, we examined whether Batten disease-causing CLN8 missense mutations impair its enzymatic activity. We selected 11 mutations spanning a range of disease onset and severity, diverse amino acid chemistries and distributed across different regions of the protein^24–32^ (Fig. 3a). Mutations affecting conserved active-site residues that we showed above are essential for catalysis (H139Y, R204C and R204L; Fig. 2b,c) were not analysed^28,33,34^. Using plasmid-based overexpression, we first assessed whether these mutant variants altered CLN8 expression or the ability to restore BMP levels in CLN8-KO HeLa cells. Given the strong plasmid-driven overexpression, which produces protein levels far exceeding endogenous CLN8, the assay functioned as a binary test of residual activity: even substantially less active variants would be expected to restore BMP to WT levels. Two variants, A30P and L207R, were not expressed, probably owing to protein destabilization (Extended Data Fig. 7a). Of the remaining mutants, T170R and G237R failed to restore BMP in CLN8-KO cells, indicating a complete loss of activity (Extended Data Fig. 7b). Both uncharged residues are positioned adjacent to the active site, where substitution with a bulky, positively charged arginine probably perturbs the local electrostatic environment and thereby abolishes catalysis (Fig. 3a and Extended Data Fig. 7c). The other variants restored BMP in CLN8-KO cells, suggesting partial activity, and were therefore selected for further in vitro characterization.

**Extended Data Fig. 8:**
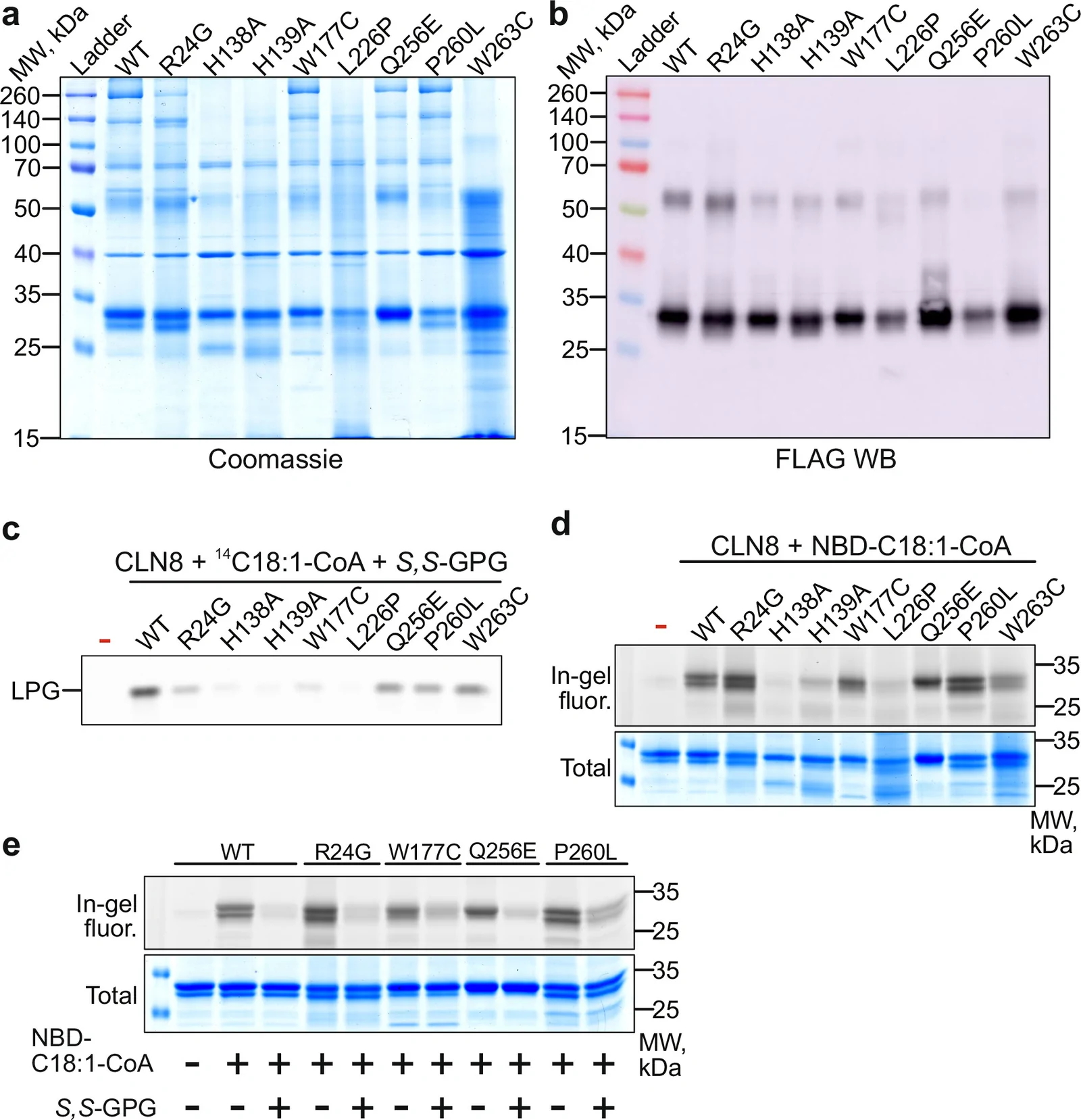
Purification and in vitro characterisation of CLN8 Batten disease mutant variants. **a**, Coomassie-stained SDS–PAGE showing total protein in eluate fractions from purification of FLAG–STREP–tagged CLN8 mutant variants. Eluates were passed through size-exclusion spin columns to remove biotin and used directly in in vitro assays. **b**, Western blot detection of FLAG in the same fractions as in (**a**). **c**, Phosphorimaging of a TLC plate showing enzymatic activity of purified CLN8 variants with oleoyl-CoA (with [1-^14^C]-oleoyl-CoA tracer) and *S,S*-GPG as substrates. **d**, In-gel NBD fluorescence (top) and Coomassie staining (bottom) of SDS–PAGE of purified CLN8 variants incubated with NBD–18:1–CoA alone. **e**, As in (**d**), but incubated with both NBD-18:1-CoA and *S,S*-GPG as indicated. Migration of LPG was validated using a chemical standard. Protein purification gels and blots in **a** and **b**, and in vitro assay data in **c**–**e**, are representative of two independent protein purifications. Unprocessed gels, blots and scans are available in source data.

We purified six Batten disease-associated CLN8 mutants capable of restoring BMP levels, alongside WT CLN8 and catalytically inactive H138A and H139A active-site mutants (Extended Data Fig. 8a,b). In initial rate assays using mixed acyl-CoA, *S*,*S*-GPG and purified proteins, all six disease mutants displayed markedly reduced activity relative to WT CLN8, whereas H138A and H139A were completely inactive (Fig. 3b). Similar results were obtained using ^14^C18:1-CoA as an acyl donor (Extended Data Fig. 8c).

The impact of each mutation on enzymatic activity broadly correlated with reported disease severity, with milder mutations tending to retain more activity than severe ones (Fig. 3a,b). However, exceptions emerged—namely, W263C causes late-infantile onset disease^28^, phenotypically analogous to a complete CLN8 loss of function, yet retained ~25% of WT activity (Fig. 3b). This suggests that additional factors not investigated here, such as CLN8 protein stability, post-translational regulation in cells or patient genetic background, may influence disease onset and progression.

To define the catalytic steps disrupted by each mutation, we monitored formation of the fluorescent NBD-C18:1-CLN8 intermediate. The L226P and W263C variants showed reduced fluorescence relative to WT on incubation with NBD-C18:1-CoA, consistent with a defect in the initial acylation (‘ping’) step (Extended Data Fig. 8d). By contrast, the W177C and P260L mutants formed the intermediate similarly to WT CLN8 but showed only partial consumption in the presence of *S*,*S*-GPG, indicating impaired *S*,*S*-GPG binding or positioning in the active site (Extended Data Fig. 8d,e). The R24G and Q256E variants formed the intermediate with WT efficiency and its levels similarly decreased in the presence of *S*,*S*-GPG, suggesting a slower rate of *S*,*S*-LPG product release in these mutants (Extended Data Fig. 8d,e).

To assess whether the enzymatic defects caused by CLN8 mutations translate into BMP deficiency in vivo, we analysed two complementary mouse models: *Cln8*^*mnd*^, a spontaneous *Cln8* frameshift allele widely used as a CLN8 Batten disease model^35–37^, and *Cln8*^*R24G*^, a knock-in allele leading to the R24G missense substitution that was engineered to model the later-onset CLN8 disease variant called Northern epilepsy, which is enriched in the Finnish population^32,38^. Consistent with complete loss of CLN8 function, homozygous *Cln8*^*mnd/mnd*^ mice displayed a complete loss of detectable BMP across brain, liver and circulation (Fig. 3c,d and Extended Data Fig. 7d). Moreover, C22:6-LPG levels were profoundly reduced and GPG levels markedly elevated, supporting the expected substrate–product relationship (Extended Data Fig. 7e,f).

In homozygous *Cln8*^*R24G/R24G*^ brains, BMP abundance was also markedly reduced in both sexes compared with WT controls (Fig. 3e). Notably, although the dominant C22:6-containing BMP species were still strongly reduced, they were relatively better preserved than other BMP species, consistent with the preferential retention of C22:6-CoA activity (~50% of WT) compared with other acyl-CoA substrates (~25%) for purified R24G mutant in vitro (Fig. 3b,e). Located at the TM1–TM4 interface and distant from the active site, R24 probably stabilizes the CLN8 fold by mediating electrostatic interactions that tether TM1 to TM4 (Fig. 3f). We propose that, in R24G homozygous patients, these residual C22:6-containing brain BMP species sustain enough lysosomal function to delay disease onset and that strategies aimed at stabilizing BMP levels, such as inhibiting its degradation via PLA2G15^20^, may offer therapeutic benefit in patients where some CLN8 activity is retained.

Taken together, our biochemical and in vivo data show that CLN8 mutations cause a graded loss of acyltransferase activity that tracks with both tissue BMP levels and disease severity. Thus, impaired BMP synthesis emerges as the crucial biochemical defect underlying CLN8 Batten disease.

### *S*,*S*-LPG restores BMP synthesis in CLN8 deficiency

**Fig. 4:**
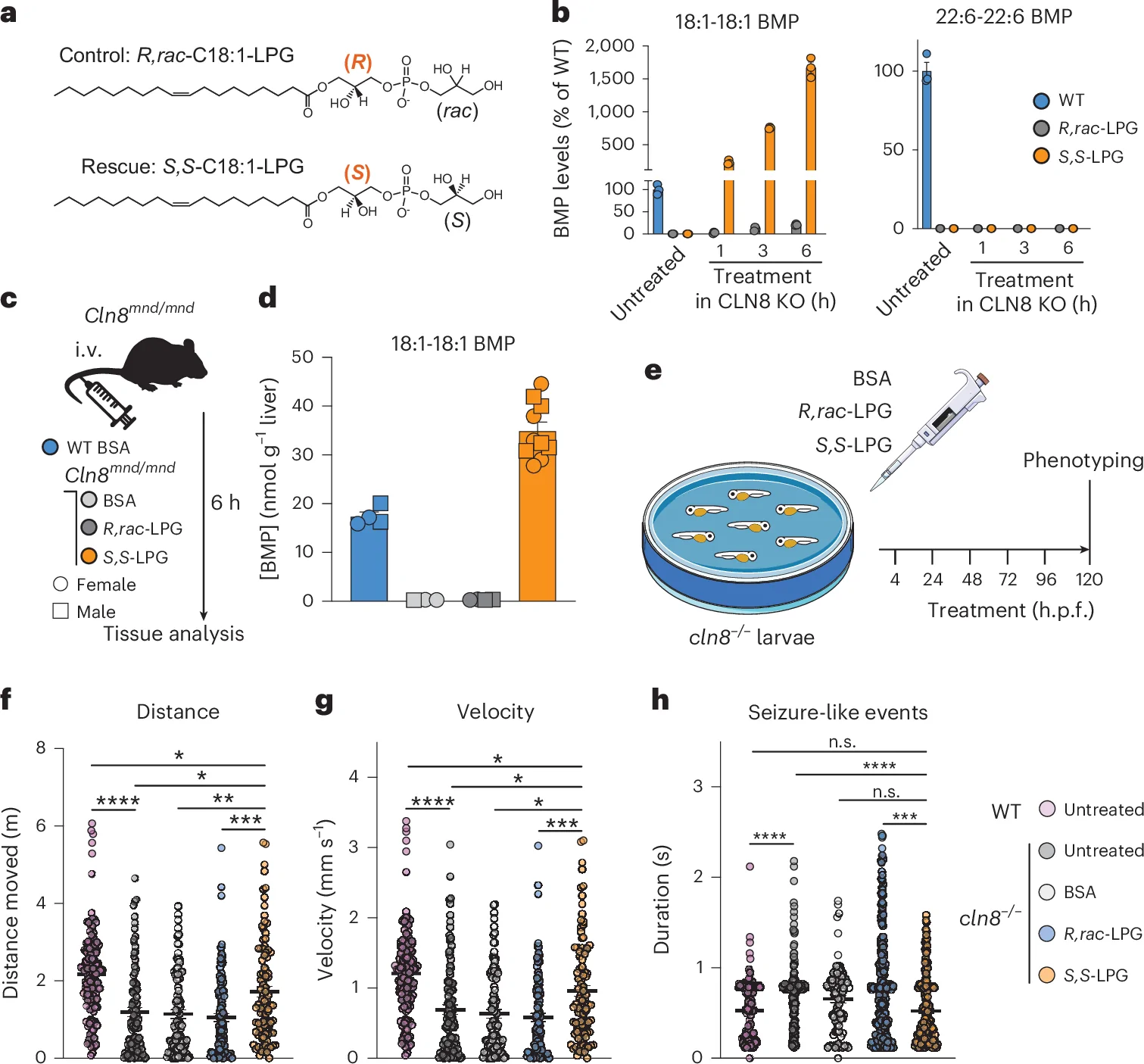
*S*,*S*-LPG restores BMP synthesis in CLN8-deficient models. **a**, Chemically synthesized LPG stereoisomers used in rescue experiments. The rescue and control compounds differ only in the stereochemistry of the glycerol backbone. **b**, Levels of 18:1–18:1 and 22:6–22:6 BMP species in WT or CLN8-KO HeLa cells treated with 10 µM *R*,*rac*-LPG or *S*,*S*-LPG for the indicated times (*n* = 3 biological replicates). WT cells were untreated. **c**, Schematic of the intravenous LPG injection protocol in homozygous *Cln8*^*mnd/mnd*^ and littermate WT mice (single bolus of 2 μmol BSA-conjugated lipid or BSA-only control). i.v., intravenous. **d**, Hepatic 18:1–18:1 BMP levels in 10-week-old *Cln8*^*mnd/mnd*^ mice 6 h after injection with *S*,*S*-LPG (*n* = 5 females, 5 males), *R*,*rac*-LPG (*n* = 2 females, 2 males) or BSA vehicle (*n* = 2 females, 1 male) and in BSA-injected WT littermate controls (*n* = 2 females, 2 males). **e**, Schematic of the *cln8*^−/−^ zebrafish larvae treatment protocol (compound exposure at a final lipid concentration of 5 µM from 4 to 120 h.p.f. with daily refresh; locomotor assays and local field potential recordings at 120 h.p.f.). **f**,**g**, Locomotor activity (distance travelled (**f**) and swimming velocity (**g**)) recorded over 30 min in WT and *cln8*^−/−^ larvae at 120 h.p.f. (WT untreated *n* = 126; *cln8*^−/−^ untreated *n* = 97, BSA *n* = 97, *R*,*rac*-LPG *n* = 103, *S*,*S*-LPG *n* = 95; three independent experiments). **h**, Duration of seizure-like events detected by local field potential recordings at 120 h.p.f. (total seizure-like events analysed with eight larvae per group: WT, *n* = 133; *cln8*^−/−^, *n* = 124; *cln8*^−/−^ + BSA, *n* = 85; *cln8*^−/−^ + *R*,*rac*-LPG, *n* = 266; *cln8*^−/−^ + *S*,*S*-LPG, *n* = 184). All data shown in graphs are presented as mean ± s.e.m. Statistical analysis in **f**–**h** was performed using the Kruskal–Wallis test followed by Dunn’s post hoc test (*P < 0.05; **P < 0.01; ***P < 0.001; ****P < 0.0001). Icons in **e** adapted from Servier Medical Art via Bioicons under a Creative Commons license CC by 3.0. Source data

Finally, we tested whether supplementing *S*,*S*-LPG could restore BMP levels in CLN8-deficient models. As *S*,*S*-LPG serves as both the acyl donor and acceptor for CLN5 during *S*,*S*-BMP synthesis, its supplementation is expected to yield a single symmetric BMP species in which both acyl chains match the supplemented *S*,*S*-LPG. We synthesized *S*,*S*-LPG containing a C18:1 acyl chain owing to its relative chemical stability and because the resulting 18:1–18:1 BMP is abundant in both cultured cells and animal tissues (Fig. 4a). As a control, we used chemically synthesized *R*,*rac*-C18:1-LPG (an equal mixture of *R*,*R*-C18:1-LPG and *R*,*S*-C18:1-LPG) (Fig. 4a). This material is commercially available and chemically analogous to *S*,*S*-C18:1-LPG but carries the wrong stereochemistry to enter the CLN5-dependent step of the BMP synthesis pathway when CLN8 is absent.

**Extended Data Fig. 9:**
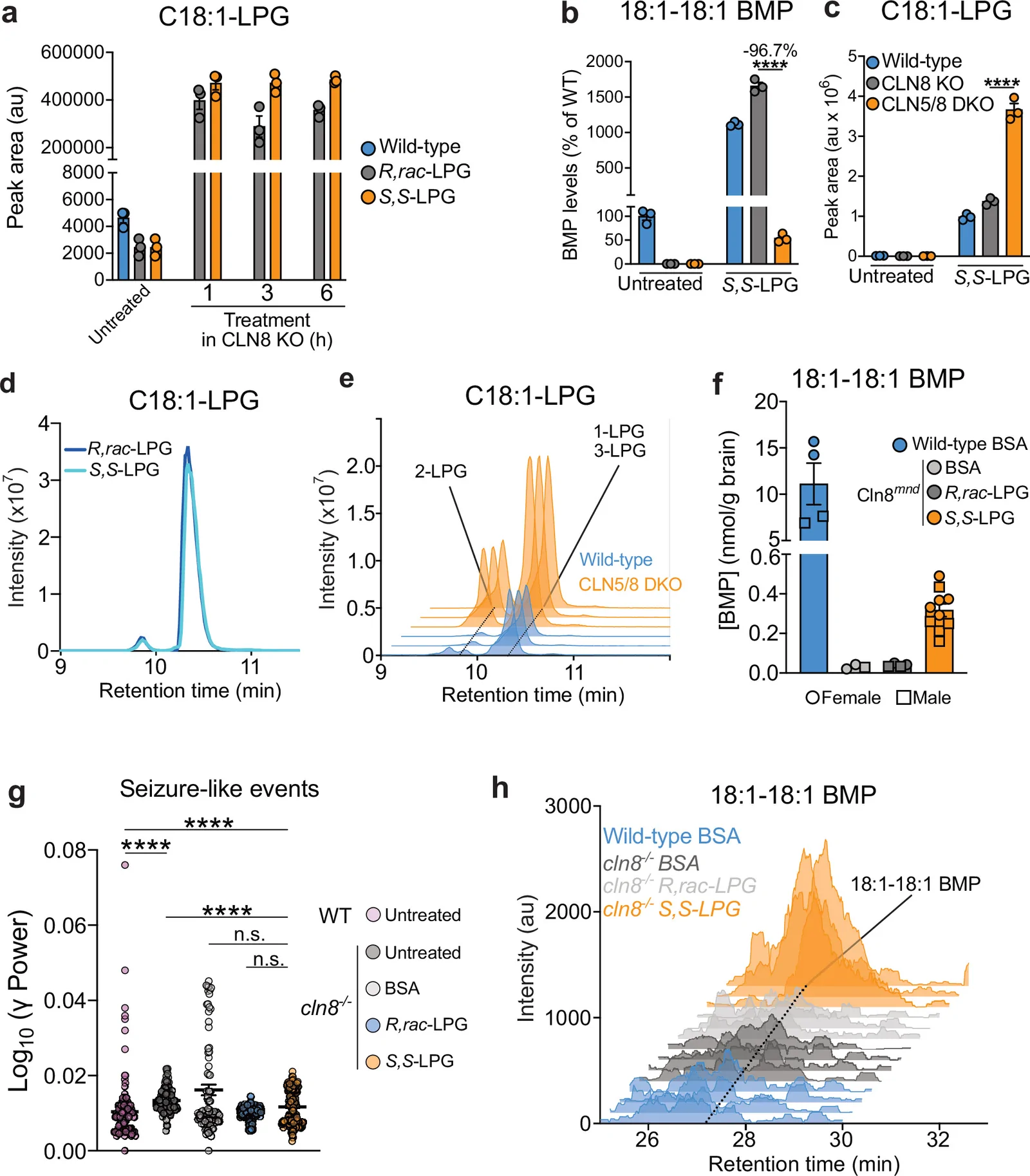
Properties of *S,S*-LPG-mediated rescue in CLN8-deficient models. **a**, Levels of C18:1-LPG species in wild-type and CLN8-knockout HeLa cells treated with 10 µM *R,rac*-LPG or *S,S*-LPG for the indicated times (n = 3 biological replicates). Wild-type cells were untreated. **b**, Levels of 18:1-18:1 BMP and **(c)** C18:1-LPG species in wild-type, CLN8 KO, and CLN5/8 DKO HeLa cells untreated or treated with 10 µM *S,S*-LPG for 6 h (n = 3 biological replicates). **d**, Synthetic *R,rac*- and *S,S*-LPG standards used in rescue experiments, analysed at equal amounts using our LC-MS workflow. **e**, LC-MS/MS chromatograms of C18:1-LPG in control and CLN5/8 DKO HeLa cells treated with 10 µM S,S-LPG for 6 h, resolved against positional isomer standards. **f**, Brain 18:1-18:1 BMP levels in 10-week-old *Cln8*^*mnd/mnd*^ mice 6 h after injection with *S,S*-LPG (n = 5 females, 5 males), *R,rac*-LPG (n = 2 females, 2 males) or BSA vehicle (n = 2 females, 1 male), and in BSA-injected wild-type littermate controls (n = 2 females, 2 males). **g**, Power of seizure-like events detected by local field potential recordings at 120 hpf (total seizure-like events analysed with 8 larvae per group: WT, n = 133; *cln8*^−/−^, n = 124; *cln8*^−/−^ + BSA, n = 85; *cln8*^−/−^ + *R,rac*-LPG, n = 266; *cln8*^−/−^ + *S,S*-LPG, n = 184). h, LC-MS/MS chromatograms of 18:1-18:1 BMP in larvae at 120 hpf: BSA-treated wild-type larvae (*n* = 4) and *cln8*^−/−^ larvae treated with BSA (*n* = 4), *R,rac*-LPG (*n* = 3) or *S,S*-LPG (*n* = 3). All data shown in graphs are presented as mean ± s.e.m. In **(b,c)**, ****P < 0.0001 by two-way ANOVA with Sidak’s post-hoc test. In (g), statistical analysis was performed using the Kruskal-Wallis test followed by Dunn’s post-hoc test (*p < 0.05; **p < 0.01; ***p < 0.001; ****p < 0.0001). Source numerical data are available in source data. Source data

In CLN8-KO HeLa cells, supplementation with BSA-conjugated *S*,*S*-C18:1-LPG, but not *R*,*rac*-C18:1-LPG, triggered a rapid and selective rise in 18:1–18:1 BMP, driving its levels well beyond those of untreated WT cells (Fig. 4b). As expected for the direct condensation of two *S*,*S*-LPG molecules by CLN5, other BMP species, including 22:6–22:6 BMP, remained undetectable, since CLN8-KO cells lack the capacity to introduce new acyl groups into *S*,*S*-GPG (Fig. 4b). Comparable increases in total cellular C18:1-LPG levels after both treatments confirmed that *S*,*S-* and *R*,*rac*-LPG were taken up with similar efficiency (Extended Data Fig. 9a). Notably, 18:1–18:1 BMP formation following *S*,*S*-C18:1-LPG supplementation was CLN5 dependent, as it was abolished in CLN5/8-DKO HeLa cells (Extended Data Fig. 9b). In these cells, C18:1-LPG accumulated to levels exceeding those in CLN8-KO cells, consistent with a block in its conversion to *S*,*S*-BMP (Extended Data Fig. 9c). Interestingly, although the chemically synthesized *S*,*S*-C18:1-LPG used in our rescue experiments is acylated at the *sn*-3 position, its accumulation in CLN5/8-DKO HeLa cells was accompanied by the substantial appearance of an *sn*-2-acylated C18:1-LPG pool (Extended Data Fig. 9d,e). This suggests that cells possess as-yet-unknown mechanisms for LPG acyl chain rearrangement, and potentially BMP acyl chain rearrangement, that operate independently of the CLN8–CLN5 biosynthetic pathway.

Next, we asked whether exogenous *S*,*S*-LPG could restore BMP synthesis in CLN8-deficient animals. To test this, we intravenously injected homozygous *Cln8*^*mnd/mnd*^ mice with a bolus of BSA-conjugated *S*,*S*-LPG or *R*,*rac*-LPG (2 μmol per mouse) and quantified hepatic 18:1–18:1 BMP 6 h later (Fig. 4c). BSA-injected WT littermates were analysed in parallel to establish the physiological baseline for this BMP species. *S*,*S*-LPG administration led to the appearance of 18:1–18:1 BMP in the liver, restoring it to levels above those detected in WT controls, whereas this species remained undetectable in vehicle- and *R*,*rac*-LPG-treated controls (Fig. 4d). Thus, exogenous *S*,*S*-LPG—the CLN8 reaction product—can bypass CLN8 deficiency and restore BMP synthesis in vivo. However, recovery of 18:1–18:1 BMP in the brains of *S*,*S*-LPG-treated *Cln8*^*mnd/mnd*^ mice was substantially poorer, reaching only a few per cent of the levels detected in BSA-injected WT controls (Extended Data Fig. 9f). We attributed this to poor blood–brain barrier permeability of C18:1-LPG in adult mice and therefore reasoned that a model with a more permissive early developmental barrier would be better suited for an initial in vivo assessment of *S*,*S*-LPG-mediated rescue of CLN8-dependent neurological phenotypes.

For this purpose, we chose *cln8* mutant zebrafish, whose larvae recapitulate key pathological signatures of CLN8 Batten disease—namely, impaired motor function, seizure-like brain activity and lysosomal dysfunction^39^. Importantly, zebrafish blood–brain barrier formation begins at approximately 72 h post-fertilization (h.p.f.) but does not fully mature until around 10 days after fertilization^40^, providing a developmental window in which exogenous *S*,*S*-LPG may access the nervous system more readily than in adult mice. *Cln8*^−/−^ larvae were treated with LPG stereoisomers or vehicle by adding the compounds directly to the fish water from 4 to 120 h.p.f., and locomotor behaviour and seizure-like activity were assessed at 120 h.p.f. (Fig. 4e). *S*,*S*-LPG treatment increased both distance travelled and swimming velocity compared with untreated, vehicle-treated and *R*,*rac*-LPG-treated mutants (Fig. 4f,g). Local field potential recordings at the same stage similarly revealed shorter seizure-like events in *S*,*S*-LPG-treated larvae, whereas event power showed high variability across all treatment groups (Fig. 4h and Extended Data Fig. 9g). Using our targeted LC–MS/MS methodology, 18:1–18:1 BMP was below the detection limit in WT larvae at 120 h.p.f. but was detectable in *S*,*S*-LPG-treated, but not *R*,*rac*-LPG-treated, *cln8*^−/−^ larvae (Extended Data Fig. 9h). Although this limits quantitative assessment of BMP restoration in zebrafish, it is consistent with pathway engagement in vivo. The low detectability of 18:1–18:1 BMP at 120 h.p.f. larvae may reflect developmental regulation of BMP synthesis, consistent with higher *cln8* mRNA abundance at 0 and 72 h.p.f. than at 120 h.p.f. (ref. ^39^), and the onset of zebrafish blood–brain barrier formation from approximately 72 h.p.f. (ref. ^40^) may allow greater *S*,*S*-LPG uptake during the early treatment window that is not captured by our 120-h.p.f. lipid analysis snapshot. Together, these data show that *S*,*S*-LPG treatment in *cln8*^−/−^ zebrafish leads to BMP formation and ameliorates key behavioural and electrophysiological deficits in vivo. These findings support BMP deficiency as a central driver of CLN8 Batten disease pathology and identify BMP precursor supplementation as a promising therapeutic strategy.

## Discussion

**Fig. 5:**
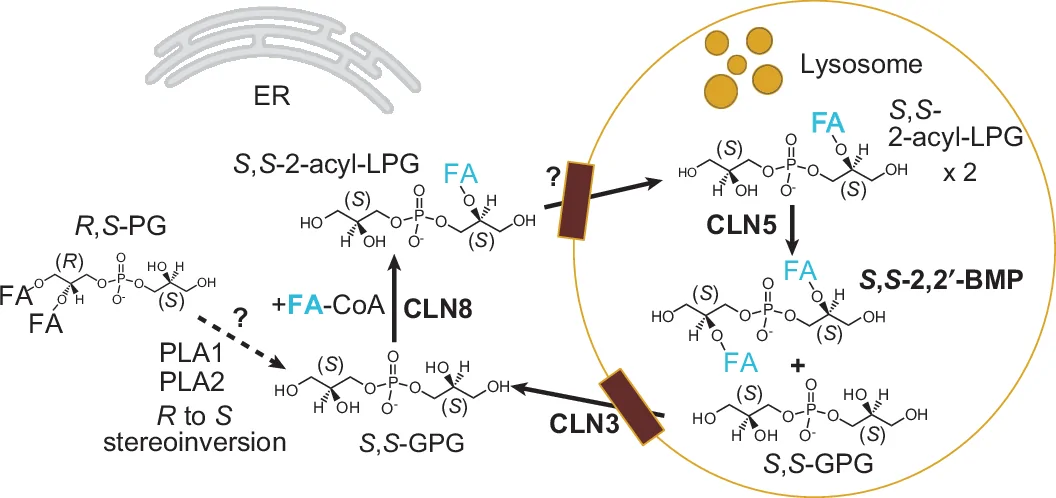
Working model of BMP biosynthesis. *R*,*S*-PG is first deacylated and undergoes *R*-to-*S* stereoinversion through an undefined mechanism to generate *S*,*S*-GPG. CLN8 then catalyses the key acyl transfer step, converting *S*,*S*-GPG to *S*,*S*-2-acyl-LPG at the ER. *S*,*S*-2-acyl-LPG enters the lysosomal lumen via an unknown route, where CLN5 uses it as both acyl donor and acyl acceptor to complete *S*,*S-*BMP synthesis, producing *S*,*S*-GPG as a by-product. The positional configuration of the CLN5-synthesized BMP product was not directly established in this study, although previous work indicates that lysosomal BMP predominantly adopts the 2,2′-acyl configuration. CLN5-generated *S*,*S*-GPG is exported back to the ER in a CLN3-dependent manner, where it can be re-acylated by CLN8. BMP degradation by lysosomal lipases also yields *S*,*S*-GPG, which is similarly exported via CLN3 to re-enter the pathway. As CLN8 generates the pool of *S*,*S*-2-acyl-LPG used by CLN5, its enzymatic activity ultimately defines the acyl composition of BMP species. PLA, phospholipase A.

In this work, we identify CLN8 as a stereospecific GPG acyltransferase that catalyses the formation of *S*,*S*-2-acyl-LPG, a key intermediate in the BMP synthesis pathway (Fig. 5). A separate study supports this conclusion by also independently identifying GPG as an intermediate in BMP biosynthesis^41^. The physiological source of *S*,*S*-GPG remains to be fully defined, but it is probably generated through PG deacylation followed by stereochemical conversion of the *R*,*S*-GPG backbone (Fig. 5). Its low abundance is consistent with its role as a transient pathway intermediate, while reported increases in GPG during ageing suggest that its steady-state level may be dynamically regulated by lysosomal stress and pathway activity^42^.

Our pathway model builds on the previously described CLN5-dependent lysosomal transacylation reaction, in which two LPG molecules are converted to BMP in the lysosomal lumen^4^ (Fig. 5). An alternative model proposes that PLD3 and PLD4 enzymes generate BMP through a distinct lysosomal transphosphatidylation reaction between LPG and monoacylglycerol^43^. Both models therefore involve LPG as a substrate but differ in how mature BMP is formed. Our study addresses the preceding step: the acyl-CoA-dependent generation of LPG by CLN8 outside the lysosome. Thus, our identification of CLN8 defines how the LPG intermediate is produced, while the precise lysosomal reaction by which LPG is subsequently converted to BMP remains to be resolved in future work.

The spatial organization of this pathway raises an important trafficking problem. CLN8 localizes to the ER/Golgi, whereas the final acyl transfer reaction that completes BMP synthesis occurs in the lysosomal lumen, requiring the CLN8-generated *S*,*S*-2-acyl-LPG intermediate to move between compartments (Fig. 5). How this intermediate reaches the lysosomal lumen remains unresolved but may involve membrane contact sites and a dedicated lysosomal lysophospholipid transporter that has yet to be identified.

This pathway architecture means that CLN8 determines the acyl composition of mature BMP species during synthesis. To remodel BMP acyl tails, BMP must first be deacylated in the lysosome to *S*,*S*-GPG, exported back to the ER in a CLN3-dependent manner^44^ and then re-acylated by CLN8 to re-enter the pathway (Fig. 5). Accordingly, CLN8 substrate selectivity and cellular acyl-CoA composition together shape BMP molecular diversity, with the enzyme’s strong preference for C22:6-CoA explaining the enrichment of C22:6-containing BMP species in the brain.

The structural and mechanistic insights presented here open opportunities for targeted modulation of BMP synthesis. The CLN8 paralogue CERS6 is inhibited by the fungal toxin fumonisin B1, which acts as an acyl acceptor and traps itself within the enzyme cavity once acylated^21^. By analogy, our structural data enable the rational design of similar acyl-accepting inhibitors targeting the cytoplasm-facing catalytic cavity of CLN8. Such compounds would serve as valuable chemical tools to dissect the physiological roles of BMP in diverse contexts and could have therapeutic relevance in conditions where transient suppression of lysosomal catabolism is advantageous.

The essentiality of CLN8 for BMP synthesis is underscored by the complete loss of all BMP species in the commonly used *Cln8*^*mnd/mnd*^ Batten disease mouse model. This striking phenotype is mirrored in CLN8-KO human cell lines^3^, highlighting the conserved and non-redundant role of CLN8 in BMP biosynthesis. Importantly, BMP synthesis can be restored by supplementing *S*,*S*-LPG—the CLN8 reaction product—in CLN8-deficient human cells and homozygous *Cln8*^*mnd/mnd*^ mouse liver. Furthermore, this metabolic rescue improves the neurological phenotypes of CLN8-deficient zebrafish larvae, demonstrating that BMP deficiency is the primary driver of neuropathology in CLN8 Batten disease. Supporting this, Batten disease-causing CLN8 missense mutations reduce CLN8 enzymatic activity and BMP levels in proportion to clinical severity.

The therapeutic potential of *S*,*S*-LPG now justifies further rescue studies in mammalian CLN8-deficient models. Conceptually, *S*,*S*-LPG provides a precursor-bypass strategy: it replaces the missing direct product of CLN8 and allows the endogenous CLN5-dependent pathway to generate BMP. However, our pilot mouse studies indicate that intravenous administration of BSA-conjugated *S*,*S*-C18:1-LPG results in only minimal 18:1–18:1 BMP formation in the brain over the acute treatment window, suggesting limited blood–brain barrier permeability of C18:1-LPG in adult mice. Optimizing the molecular properties of *S*,*S*-LPG, including acyl chain length and unsaturation, will therefore be important for improving in vivo delivery, brain penetration and rescue efficacy. Direct *S*,*S*-BMP supplementation may also be considered, although it is likely to face additional challenges related to aqueous solubility, membrane insertion, cellular uptake and lysosomal delivery.

The rescue experiments presented here should therefore be viewed as proof-of-concept studies showing that BMP production can be pharmacologically restored with functional benefit, rather than as optimized reconstitution of endogenous BMP diversity and function. Similarly, the exogenous lipids and tracers used here were applied at concentrations designed to establish enzymatic specificity and pathway connectivity, not to define physiological pathway flux. Future studies are therefore needed to define whether BMP precursors can be modified to improve blood–brain barrier penetration, to determine the turnover of endogenous brain BMP pools and to quantify flux through the BMP biosynthetic pathway under physiologically relevant conditions.

Although the human cell lines used in this study provided the genetic tractability required for mechanistic dissection of the pathway, human neuronal models are now essential to define how this pathway is regulated in physiologically relevant cells. In future studies, CLN8-deficient induced pluripotent stem cell-derived neurons and brain organoids will provide a lipid metabolic context more directly relevant to Batten disease and will establish whether optimized *S*,*S*-LPG analogues can restore BMP production and lysosomal function before further in vivo testing.

More broadly, as additional Batten disease genes, including *CLN3*^5^ and *CLN5*, participate in BMP homeostasis, BMP deficiency may represent a unifying pathogenic mechanism across multiple Batten disease subtypes—a hypothesis that now demands systematic investigation.

## Methods

All research was conducted in accordance with the relevant ethical regulations and institutional guidelines. As the study involved work performed across multiple collaborating institutions, the appropriate approving ethics committees, institutional review boards and animal-welfare bodies, together with the corresponding protocol or licence details, are specified in the relevant subsections in the Methods.

### Reagents and antibodies

Comprehensive lists of the antibodies and the chemicals/reagents used in this study are presented in Supplementary Tables 3 and 4, respectively.

### Plasmids

Details of plasmids used in this study are presented in Supplementary Table 5.

### Mouse studies

Tissues from age-matched (6-month-old) WT and homozygous *Cln8*^*mnd/mnd*^ mice were obtained from Sanford Research (Sioux Falls, SD, USA) for BMP, LPG and GPG analysis. Animals were bred, housed and killed under standardized conditions in an Association for Assessment and Accreditation of Laboratory Animal Care (AAALAC) International-accredited facility, in accordance with protocols approved by the Sanford Research Institutional Animal Care and Use Committee (IACUC; USDA licence 46-R-0009; protocol 2023-0102). Mice were group-housed, with up to five animals per cage, in individually ventilated cages with HEPA-filtered airflow (Tecniplast Green Line cage system), under a 10-h dark–14-h light cycle at 24 ± 1 °C and 30–70% relative humidity. Animals had ad libitum access to irradiated Teklad 2018 chow and non-acidified water. Teklad 7092–7097 corncob bedding was used, with Teklad 7099 laboratory animal bedding provided as enrichment (Teklad). All procedures complied with institutional, federal and international guidelines for the humane care and use of research animals.

Tissues from age-matched (8-week-old) WT and homozygous *Cln8*^*R24G/R24G*^ mice^38^ and their littermate controls were obtained from the University of Oulu (Finland). Animal care was carried out with the support of the Oulu Laboratory Animal Centre Research Infrastructure, University of Oulu (Finland). Animals were kept on 12-h light–dark cycle and had unlimited access to chow (Teclad 2018/2918, Inotiv) and water. All experimental procedures were conducted in accordance with the Finnish national legislation, EU Directive 2010/63/EU, and the ethical standards of the University of Oulu. All animal procedures were approved by the Project Authorisation Board at the State Provincial Office of Southern Finland (ESAVI/33743/2019 and ESAVI/16903/2024).

The LPG rescue experiment complied with all relevant ethical regulations and was conducted in accordance with Animal Research: Reporting of in Vivo Experiments (ARRIVE) 2.0 guidelines. All procedures were reviewed and approved by the University of Cambridge Animal Welfare and Ethical Review Body. Animal work and breeding was performed under the UK Animals (Scientific Procedures) Act 1986 (Home Office Project Licence PPL no. PP1740969) and in line with EU Directive 2010/63/EU. The *Cln8*^*mnd*^ mouse line was obtained from The Jackson Laboratory, and homozygous experimental animals and littermate WT controls were generated by crossing heterozygous parents. Both female and male mice were used in accordance with the 3Rs principles. Mice were housed in individually ventilated cages (Tecniplast) at 20–24 °C and 45–65% humidity on a 12 h light–dark cycle, with ad libitum access to SAFE 105 universal diet (Safe Diets) and water.

For the LPG rescue experiment, custom-synthesized *S*,*S*-C18:1-LPG was dissolved in chloroform, dried under a stream of nitrogen to remove solvent and the resulting lipid film reconstituted in 12% fatty acid- and endotoxin-free BSA (A8806, Sigma) prepared in 150 mM NaCl to a final concentration of 20 mM, using gentle heating and sonication. The control lipid (*R*,*rac*-C18:1-LPG; 858125, Avanti) was prepared in parallel using the same procedure. Conscious mice received an intravenous tail vein injection of 100 µl of lipid–BSA complex (or the matched BSA vehicle in 150 mM NaCl) and were killed 6 h later by cervical dislocation. Brains and livers were immediately snap frozen for lipid analysis.

### Zebrafish studies

Zebrafish experiments were performed using a previously characterized *cln8*^−/−^ zebrafish line^39^. Homozygous mutants were generated by intercrossing adult *cln8*^−/−^ males and females. Adult fish were maintained at 28 °C on a 14 h light–10 h dark cycle at a density of five or fewer fish per litre. Embryos were collected and raised at 28.5 °C in egg water from ‘Instant Ocean’ sea salts (60 µg ml^−1^) or E3 medium (292.2 mg NaCl, 12.6 mg KCl, 48.6 mg CaCl_2_·2H_2_O, 39.8 mg MgSO_4_ per litre), prepared according to standard protocols^45^. Developmental stages were recorded as h.p.f. All zebrafish procedures were carried out in accordance with EU Directive 2010/63/EU and were approved by the Italian Ministry of Health (authorization no. 1090/2024-PR) under the oversight of the IRCCS Fondazione Stella Maris Animal Care and Use Committee.

For LPG rescue experiments, *S*,*S*-C18:1-LPG and *R*,*rac*-C18:1-LPG control were prepared as described above but reconstituted in BSA at a final concentration of 10 mM. Both treatments, or an equivalent amount of BSA control, were added directly to egg water at a final concentration of 5 µM, and the treatments were repeated every 24 h until phenotypic analysis.

Larval motor behaviour was assessed as described^39^. Locomotor activity (distance travelled and velocity) was measured in *cln8*^−/−^ siblings at 120 h.p.f. using the DanioVision system with EthoVision XT automated tracking.

Electrophysiological recordings were performed in the forebrain of 120-h.p.f. larvae to assess seizure-like activity (epilepsy-like phenotype), following established procedures^46^.

For LC–MS/MS analyses, three to four independent larval pellets were collected per experimental group at 120 h.p.f., with each pellet containing 50 larvae. Larvae were pooled, washed once in egg water, excess medium was carefully removed and pellets were frozen and stored at −70 °C until lipid extraction.

### Molecular biology

Site-directed mutagenesis was performed using the Q5 Site-Directed Mutagenesis Kit (E0554S, New England Biolabs) on pcDNA3.1(+)-C-HA and pcDNA5/FRT/TO plasmids containing WT CLN8 inserts, following the manufacturer’s instructions. Resulting mutant constructs were verified by Sanger sequencing using CMVFpCDNA3 and BGHRev primers (for pcDNA3.1(+)-C-HA) or pCMVF and BGHRev primers (for pcDNA5/FRT/TO), performed by Source Genomics. In cases where pcDNA3.1(+)-C-HA constructs did not yield detectable CLN8 mutant protein expression, plasmid integrity and sequence were further validated by whole-plasmid Oxford Nanopore sequencing.

### Human cell culture

HeLa (ECACC 93021013) cell line was obtained from Culture Collections, UK Health Security Agency. The Flp-In T-REx 293 cell line was purchased from Thermo Fisher Scientific (R78007). All cell lines were maintained in DMEM (11965084, Thermo Fisher Scientific) supplemented with 10% fetal bovine serum (FBS; Sigma-Aldrich) at 37 °C in 5% CO_2_. Cells were passaged at ~90% confluence using TrypLE Express (12605010, Thermo Fisher Scientific) and routinely tested for mycoplasma contamination.

For transient transfection, cells cultured in standard growth medium were transfected with FuGENE HD (E2311, Promega) following the manufacturer’s protocol. In brief, for one well of a six-well plate, 20 μl Opti-MEM (31985062, Thermo Fisher Scientific), 0.4 μg plasmid DNA and 1.2 μl FuGENE HD were mixed. Medium was replaced the next day, and cells were analysed 48–72 h after transfection.

For lipid analyses, confluent cells in six-well plates were washed twice with 3 ml ice-cold PBS on ice, then scraped into 500 μl ice-cold PBS using an inverted 200-μl pipette tip. Cell suspensions were transferred to prechilled 2-ml plastic tubes (3469-11, Thermo Fisher Scientific) and centrifuged at 500*g* and 4 °C for 5 min. Supernatants were removed, and cell pellets were snap frozen and stored at −70 °C. Each well provided one replicate for lipid analysis.

For d5-PG labelling, 16:0-18:1 d5-PG (860385, Avanti) was dissolved in ethanol to prepare a 10 mM stock solution. The stock was added dropwise to complete DMEM to achieve a final concentration of 25 μM and mixed thoroughly by vortexing. Labelling was initiated by replacing the culture medium with the prewarmed d5-PG-containing medium.

For LPG rescue experiments, *S*,*S*-C18:1-LPG and *R*,*rac*-C18:1-LPG control were prepared as described above but reconstituted in BSA at a final concentration of 10 mM. Both treatments were added directly to cell culture medium at a final concentration of 10 µM, and cells were collected after the incubation times indicated in Fig. 4 and Extended Data Fig. 9.

### Human KO cell line generation

HeLa KO cell lines were generated by transfecting cells with recombinant complexes of Cas9 protein and synthetic guide RNAs (gRNAs). The HeLa CLN8-KO and corresponding control cell lines were previously established as described^3^. To generate a matched set of control, CLN8-KO, CLN5-KO and CLN5/8-double-KO (DKO) lines, both the published control and CLN8-KO cells were edited again using either a non-targeting or a CLN5-targeting CRISPR strategy, resulting in four parallel cell lines used in this study.

For transfection, cells were seeded in six-well plates in standard growth medium and transfected the following day at approximately 30% confluence. Transfections were performed using Lipofectamine CRISPRMAX Cas9 Transfection Reagent (CMAX00001, Thermo Fisher Scientific), TrueCut Cas9 Protein v2 (A36497, Thermo Fisher Scientific) and custom-synthesized TrueGuide synthetic gRNAs (Thermo Fisher Scientific), following the manufacturer’s instructions. Each CLN5-KO transfection contained 6,250 ng Cas9 protein and 600 ng of each targeting gRNA (GAAAUGUACAGUUGCCAAGU and GCCUUGAUUACACCAGAAAG), while control transfections contained 6,250 ng Cas9 protein and 1,200 ng non-targeting gRNA (A35526, Thermo Fisher Scientific) to generate the corresponding control cell lines.

After transfection, the medium was replaced after 24 h and cells were passaged into 10-cm dishes the following day. Control and KO populations were subsequently expanded in multiple 15-cm dishes, cryopreserved and later thawed for validation by western blotting and quantitative proteomics. All experiments were performed using low-passage cells following thawing.

### Human Flp-In T-REx 293 inducible cell line generation

Flp-In T-REx 293 cells were seeded in six-well plates and transfected at ~25% confluence. Transfections were performed using a mixture of 20 μl Opti-MEM, 0.36 μg pOG44 plasmid, 0.04 μg pcDNA5/FRT/TO plasmid encoding CLN8 WT or mutant and 1.2 μl FuGENE HD. Medium was replaced after 24 h, and cells were cultured for an additional 48 h before passaging into 6-cm dishes. Stable integrants were selected in standard medium containing 50 μg ml^−1^ hygromycin B for 2 weeks, with passaging on confluence. Hygromycin-resistant pools were expanded, aliquoted and cryopreserved. An aliquot was thawed and tested for inducible protein expression by tetracycline dose–response (48 h) and time-course assays (at the optimized dose). All CLN8 WT and mutant proteins were purified following induction with 1 μg ml^−1^ tetracycline for 48–72 h.

### Yeast strain generation for protein purification

*Saccharomyces cerevisiae* strain W303.1B (MATα leu2-3,112 trp1-1 can1-100 ura3-1 ade2-1 his3-11,15) (ATCC: 201238) was used for protein expression and purification. Yeast line expressing human CLN8 was generated according to the published protocol^47^. In brief, pYES2/CT C-HIS-CLN8 plasmid was transformed into competent W303.1B cells by lithium acetate/polyethylene glycol and heat shock. Transformants were selected on SC-Ura + 2% glucose agar plates at 30 °C for 48–72 h. Individual colonies were inoculated into 2 ml SC-Ura + 2% glucose liquid medium and grown overnight at 30 °C with shaking at 225 rpm. Glycerol stocks were prepared by mixing 750 μl culture with 750 μl 30% glycerol and stored at −70 °C.

### CLN8 protein purification from human cells

Flp-In T-REx 293 cells with inducible expression of C-terminal Flag-Strep-tagged CLN8 WT or mutant variants were seeded in 15-cm dishes (ten dishes per typical purification) in standard culture medium. The following day, at ~25% confluence, expression was induced with 1 μg ml^−1^ tetracycline for 48–72 h. Cells were then washed once with ice-cold PBS, scraped into ice-cold PBS on ice and pelleted by centrifugation at 500*g* and 4 °C for 5 min. Pellets were stored at −70 °C until use.

For purification, frozen pellets were thawed and resuspended in Hepes buffer (20 mM Hepes (pH 7.4), 150 mM NaCl). Solubilization was performed by adding 10% LMNG (Anatrace) to a final concentration of 1.5%, followed by incubation at 4 °C for 2 h. Lysates were cleared by centrifugation at 128,000*g* and 4 °C for 1 h. Meanwhile, 600 μl Strep-Tactin XT 4Flow bead slurry (Iba) was washed twice with Milli-Q water and once with wash buffer (20 mM Hepes (pH 7.4), 150 mM NaCl, 0.1% LMNG). The clarified supernatant was incubated with the washed beads for 2 h at 4 °C. Beads were then washed three times with wash buffer to remove unbound proteins. CLN8 was eluted in 1 ml elution buffer (50 mM Hepes (pH 7.4), 150 mM NaCl, 0.05% LMNG, 100 mM biotin). Excess biotin was removed by passage through PD Minitrap G-25 columns (17085101, Cytiva). Protein concentration was determined using a NanoDrop ND-8000 at 280 nm. Purified proteins were aliquoted and stored at −70 °C. All CLN8 mutants were purified using the same protocol alongside WT controls.

### CLN8 protein purification from yeast

W303 yeast strain expressing codon-optimized C-His-tagged human CLN8 was retrieved from glycerol stock and scaled up to 10 litres in YPG + 0.1% glucose at 30 °C with shaking (225 rpm). Expression was induced by adding galactose to a final concentration of 0.4% for 4 h. Cells were collected by centrifugation, washed with water and stored at −20 °C.

For membrane preparation, cell pellets were thawed and homogenized in breaking buffer (0.65 M sorbitol, 100 mM Tris-HCl (pH 8.0), 5 mM EDTA, 5 mM amino hexanoic acid, 5 mM benzamidine hydrochloride). Cells were lysed using a Constant Systems Cell Disruptor. Debris was cleared by centrifugation (3,000*g*, 20 min, 4 °C), and membranes were pelleted by ultracentrifugation (125,000*g*, 1 h, 4 °C). The membrane pellet was washed in wash buffer (0.65 M sorbitol, 100 mM Tris-HCl (pH 7.4), 5 mM amino hexanoic acid, 5 mM benzamidine hydrochloride), pelleted again then washed in TBG buffer (100 mM Tris-HCl (pH 7.4), 10% glycerol) and pelleted once more. The final membrane pellet was weighed, resuspended in TBG, aliquoted and stored at −70 °C.

For purification, yeast membranes were solubilized in 21 ml buffer containing 1.5% LMNG, 20 mM Hepes (pH 7.0), 150 mM NaCl, 30 mM imidazole and protease inhibitors and incubated for 1.5 h at 4 °C. Insoluble material was removed by centrifugation (118,000*g*, 60 min), and the supernatant was collected. Ni-Sepharose excel resin (Cytiva) was equilibrated with Milli-Q water and wash buffer A (20 mM Hepes (pH 7.0), 150 mM NaCl, 40 mM imidazole, 0.1% LMNG). The soluble fraction was incubated with resin for 2 h at 4 °C. Resin was washed three times with wash buffer A and once with wash buffer B (20 mM Hepes (pH 7.0), 100 mM NaCl, 2.5 mM CaCl_2_ and 0.05% LMNG). Bound proteins were resuspended in 1.5 ml buffer B, and elution was performed by overnight incubation at 4 °C with 20 μg Factor Xa protease and 2.5 mM CaCl_2_. Protein concentration was determined by measuring absorbance at 280 nm (NanoDrop ND-8000).

### SEC

CLN8 was purified from yeast as described above. The protein was concentrated to 3.5 mg ml⁻^1^ using a 10-kDa molecular-weight cutoff centrifugal concentrator (UCF501008, Millipore). A 130-µl aliquot was injected onto an ÄKTA Purifier system (GE Healthcare) equipped with a Superdex 200 Increase 10/300 GL column (GE Healthcare) equilibrated in size exclusion chromatography (SEC) buffer (50 mM Hepes (pH 7.4), 150 mM NaCl, 0.05% LMNG). Fractions corresponding to the main elution peak (indicated by dotted lines) were pooled and reconcentrated using the 10-kDa molecular-weight cutoff concentrator. The resulting sample was analysed by SDS–PAGE and in vitro activity assays, and the purified protein was used for cryo-EM grid preparation.

### Sample preparation and cryo-EM data acquisition

Cryo-EM grids were prepared using CLN8 at three concentrations (1, 2 and 3 mg ml^−^^1^). For the apo condition, the protein was incubated with 0.05% (w/v) fluorinated octyl maltoside for 45 min on ice before grid preparation. For the substrate-bound conditions, CLN8 (2 mg ml^−^^1^) was incubated in SEC buffer containing 100 µM C18:1-CoA or 100 µM C22:6-CoA for 1 h at room temperature, followed by the addition of 0.05% fluorinated octyl maltoside and incubation for 45 min on ice. For each condition, 3 µl of sample was applied to glow-discharged Quantifoil R1.2/1.3 300-mesh holey carbon copper grids (30 s at 10 mA) and vitrified in a Vitrobot Mark IV (Thermo Fisher Scientific) operated at 4 °C and 95% relative humidity (blot time, 3.0 s; blot force, −10).

Datasets were collected from a single grid per condition on a Titan Krios (Thermo Fisher Scientific) operating at 300 kV and nominal magnification ×165,000, equipped with a Falcon 4i detector and a Selectris X energy filter in counting mode. Movies were recorded in EPU v3.1 at a physical pixel size of 0.729 Å pixel^−^^1^, with a target defocus range of −1.8 to −2.4 µm in 0.2-µm increments; autofocus was executed every 10 µm of stage travel. The total exposure was 49.81, 49.89 and 50.14 e^−^ Å^−2^ for the apo, C18:1-bound and C22:6-bound datasets, respectively, with dose rates of 6.03, 6.04 and 6.07 e^−^ pixel^−^^1^ s^−^^1^. Data were acquired using aberration-free image shift (AFIS) with four exposures per hole, a 5-s delay after stage shifts and a 1-s delay after image shifts.

### Cryo-EM data processing

Processing was performed in cryoSPARC v3.3.2. For each dataset, ~9,000–10,000 movies were collected. Movie-frame alignment, contrast transfer function estimation, particle picking and particle extraction (box size, 360 pixels) were carried out in Warp^48^. Particles were initially Fourier binned to 2.35 Å per pixel and subjected to multiple rounds of 2D classification to remove artefacts, contaminants and low-quality particles. Ab initio reconstruction in cryoSPARC produced six initial classes, which were used for heterogeneous refinement to separate particle populations (for example, CLN8 dimers versus empty micelles). Two successive heterogeneous refinements were performed, after which selected particles were re-extracted at the full pixel size (0.729 Å per pixel) and refined using non-uniform refinement with the best heterogeneous-refinement map as reference, yielding final reconstructions at 2.69–3.65 Å resolution. Cryo-EM data collection, refinement and validation statistics are presented in Supplementary Table 1.

For the CLN8 apo dataset, an additional local refinement was performed after non-uniform refinement. A mask was generated in UCSF Chimera v1.13.1, and the final map was Gaussian filtered using ‘vop gaussian’ (*σ* = 4); the threshold was adjusted to encompass the dimeric assembly.

For the CLN8 apo dataset, additional processing was performed to investigate conformational heterogeneity in the TM4 region. HR-HAIR^23^ was performed on the full 260k particle stack using CryoSPARC with the following non-default parameters: number of classes, 2; initial resolution, 6 Å; maximum resolution, 4 Å; Fourier radius step, 0.005; initial minibatch size, 300; final minibatch size, 1,000; centre structures in real space, OFF. This approach, which uses only high spatial frequency data to assign particle orientations, revealed two discrete particle populations: a dominant class of ~143k particles yielding a 3.68 Å map and a secondary class of ~110k particles showing partial loss of density at the dimer interface. The dominant class was used for all subsequent processing and model building of the apo structure. Focused local refinement centred on TM4 was additionally performed using a soft mask encompassing the TM4 helix, with rotation search extent 2°, shift search extent 1 Å and initial lowpass resolution 6 Å.

### Model building, validation and structural analysis

AlphaFold-predicted CLN8 models^49,50^ were rigid-body fitted into the cryo-EM density in UCSF Chimera^51^, with side chains pruned to match the density. The model was rebuilt using ISOLDE^52^ within ChimeraX^53^ and further refined in Coot^54^. For the CLN8 + C18:1-CoA, CLN8 + C22:6-CoA and CLN8 apo CoA-bound datasets, clear densities were present in the CLN8 structures; the corresponding ligands were modelled with ChemEM^55^ guided by the cryo-EM maps. Restraint files for C18:1, C22:6 and CoA were taken from the CCP4 monomer library. Models were validated with MolProbity^56^. Final positional minimization and group B-factor refinement were performed in Servalcat^57^ against the cryoSPARC-refined maps. Structural figures were prepared in PyMOL (Schrödinger).

The acyl chain was oriented with its carboxyl group adjacent to H138-H139 because this places the hydrocarbon tail within the narrow hydrophobic channel and, importantly, is the only polarity compatible with accommodation of the bulky CoA moiety in the adjacent cytoplasm-facing cavity before cleavage.

The experimentally determined CLN8 structure closely matches the previously reported AlphaFold2 model^3,49^, with an overall root-mean-square deviation (RMSD) of 0.53 Å (all-atom RMSD calculated using PyMOL), indicating that the global fold was accurately predicted.

Water molecules were added to cryo-EM maps of the C18:1-CoA and C22:6-CoA states using phenix.douse^58^ and manually pruned by inspection against the map in Coot^54^. Cavity volumes were determined using MDAnalysis^59^, by measuring the solvent-accessible volume within an 8-Å radius sphere centred on the Cα atom of the catalytic His residue.

### Denaturing PAGE, in-gel fluorescence, Coomassie staining and western blotting

Twelve percent SDS–polyacrylamide gels were manually cast using Invitrogen SureCast Acrylamide Solution (40%). Samples were prepared at room temperature without heating, using Pierce Lane Marker Reducing Sample Buffer at a final concentration of 2.5×. Notably, heating led to high-molecular-weight aggregation of CLN8 proteins, consistent with observations for other transmembrane proteins^60^. Electrophoresis was performed in Tris-glycine-SDS buffer at 120 V for stacking and 180 V for resolving gels.

For Coomassie staining, gels were incubated in 15 ml InstantBlue Protein Stain (Abcam) overnight and then destained with water overnight. Stained gels were imaged using a flatbed scanner.

For in-gel NBD fluorescence, 1.5 μg purified protein was incubated with 5 μM NBD-C18:1-CoA alone or with 5 μM NBD-C18:1-CoA plus 500 μM *S*,*S*-GPG at 37 °C for 15 min. Reactions were stopped by adding sample buffer (2.5×) and then analysed by SDS–PAGE as above. Fluorescent images were acquired using an Amersham Typhoon imager (Cy2 setting), followed by Coomassie staining and imaging.

For western blotting, proteins were transferred onto 0.45-μm polyvinylidene difluoride (PVDF) membranes (Thermo Fisher Scientific) in chilled Tris-glycine buffer with 10% methanol at 90 V for 100 min using a Mini Trans-Blot system (Bio-Rad) with an ice pack. Membranes were blocked in 5% skimmed milk in TBST, and then incubated overnight at 4 °C with primary antibodies (Supplementary Table 3). After washing, membranes were incubated with secondary antibodies (1:5,000) for 1 h at room temperature. Detection was performed by enhanced chemiluminescence (Thermo Fisher Scientific) and imaged on a ChemiDoc MP system (Bio-Rad). Where indicated, blots are shown as overlays of chemiluminescence and colourimetric images to display molecular-weight markers.

### Quantitative analysis of CLN5 protein abundance

Cell pellets were lysed in 100 µl lysis buffer consisting of 1% sodium deoxycholate (Sigma, #D6750), 100 mM triethylammonium bicarbonate (Sigma, #T7408), 10% isopropanol, 50 mM NaCl, 1× protease and phosphatase inhibitor cocktail (Thermo Fisher Scientific, #78441) and nuclease (Thermo Fisher Scientific, #88700; 0.5 µl per 1 ml buffer). Lysates were briefly sonicated to improve sample dissolution, and protein concentrations were determined using the Quick Start Bradford Protein Assay (Bio-Rad) according to the manufacturer’s instructions. For each sample, 10 µg protein was reduced and alkylated in 5 mM tris(2-carboxyethyl)phosphine (TCEP; Thermo Fisher Scientific, #77720) and 10 mM iodoacetamide at room temperature for 1 h in the dark, followed by overnight digestion at room temperature with 500 ng trypsin (Pierce, #90058). Peptides were acidified with trifluoroacetic acid (TFA) to precipitate sodium deoxycholate, which was removed by centrifugation at 12,000*g*. The clarified peptide solutions were desalted using C18 Spin Tips (Pierce). In brief, C18 tips were conditioned with 80% acetonitrile (ACN; Fisher Scientific)/0.1% TFA and equilibrated with 0.1% TFA, after which acidified peptides were loaded, washed with 0.1% TFA and eluted with 80% ACN/0.1% TFA. Eluates were dried in a SpeedVac (Eppendorf) and resuspended in 40 µl 0.1% formic acid (FA); 2 µl (approximately 500 ng peptide) was injected for LC–MS analysis.

Peptides were analysed on a Vanquish Neo ultra-high performance liquid chromatography system (Thermo Fisher Scientific) coupled to an Orbitrap Fusion Lumos Tribrid MS (Thermo Fisher Scientific). Peptides were first trapped on a 300 µm × 5 mm C18 trap cartridge (5 µm, 100 Å) and then separated over a 100-min gradient on a 75 µm × 50 cm C18 reversed-phase column (2 µm, 100 Å) at a flow rate of 300 nl min^−^^1^. In each acquisition cycle, one full MS scan (*m*/*z* 375–1,200) was acquired in the Orbitrap at 60,000 resolution with a normalized automatic gain control (AGC) target of 300% and automatic maximum injection time (MIT). Data-independent acquisition (DIA) spectra were then acquired in the Orbitrap at 30,000 resolution (normalized AGC 800%, automatic MIT, HCD collision energy 30%) using 42 DIA windows across a 400–900-*m*/*z* precursor scan range.

Raw data were processed using DIA-NN (version 2.0). An in silico-predicted spectral library was generated from a human FASTA database (9606_2025_01_23) and used to analyse the DIA data, allowing one missed cleavage, N-terminal methionine excision and carbamidomethylation of cysteine as a static modification. The peptide length range was set to 7–30 amino acids, precursor charge range to 1–4, precursor *m*/*z* range to 375–1,200 and fragment ion *m*/*z* range to 145–1,450. Match-between-runs was enabled, and the quantification strategy was set to ‘QuantUMS (high precision)’. MS1 and MS2 mass accuracy and retention time window (scans) were set to 5, 15 and 8, respectively. All other DIA-NN settings were left at default, using retention time-dependent cross-run normalization.

For the present study, downstream analysis was restricted to the relative abundance of CLN5 peptides. No proteome-wide differential abundance analysis was performed, and abundance measurements for other proteins were not included in the study.

### Chemical synthesis of *R*,*S*-GPG standard

The target compound *R*,*S*-GPG ((*R*)-2,3-dihydroxypropyl ((*S*)-2,3-dihydroxypropyl) hydrogen phosphate) was synthesized through a nine-step sequence starting from (*S*)-(2,2-dimethyl-1,3-dioxolan-4-yl) methanol. In the first step, benzylation with benzyl bromide in dimethyl sulfoxide (DMSO) using sodium hydride afforded (*S*)-4-((benzyloxy)methyl)-2,2-dimethyl-1,3-dioxolane (92% yield). Subsequent acidic hydrolysis (70% acetic acid, 65 °C) yielded (*R*)-3-(benzyloxy)propane-1,2-diol (81%). Selective tritylation of the primary alcohol with trityl chloride, triethylamine and 4-dimethylaminopyridine (DMAP) in tetrahydrofuran (THF) at 80 °C gave (*S*)-1-(benzyloxy)-3-(trityloxy)propan-2-ol (81%). Further benzylation with benzyl bromide under basic conditions produced (*S*)-((2,3-bis(benzyloxy)propoxy)methanetriyl) tribenzene (86%).

Cleavage of the trityl group by heating in 70% acetic acid provided (*R*)-2,3-bis(benzyloxy)propan-1-ol (82%), which was phosphorylated with diphenyl phosphonate in pyridine to afford triethylammonium (*S*)-2,3-bis(benzyloxy)propyl phosphonate (59%). This intermediate was coupled with (*S*)-(2,2-dimethyl-1,3-dioxolan-4-yl)methanol using pivaloyl chloride activation, followed by oxidative iodine treatment, to yield triethylammonium (*S*)-2,3-bis(benzyloxy)propyl (((*R*)-2,2-dimethyl-1,3-dioxolan-4-yl)methyl) phosphate (22%). Acidic deprotection (TFA) furnished (*S*)-2,3-bis(benzyloxy)propyl ((*R*)-2,3-dihydroxypropyl) hydrogen phosphate (53%). Finally, hydrogenolysis over Pd(OH)_2_ in MeOH delivered the target diol phosphate *R*,*S-*GPG in 10% yield.

All intermediates were characterized by MS and ^1^H-NMR spectroscopy. Purifications were performed by silica or neutral alumina chromatography with appropriate solvent gradients.

### Synthesis of GPG stereoisomers

*R*,*R*-, *R*,*S*- and *S*,*S*-GPG stereoisomers were generated by mild methanolysis from *R*,*R*-BMP (857136, Avanti), *R*,*S-*BMP (857133, Avanti) and *S*,*S*-BMP (857135, Avanti), respectively. A total of 5 mg of each BMP were dissolved in 1 ml 0.1 M NaOH in methanol and incubated at 37 °C for 1 h. Reactions were neutralized with 1 M acetic acid. Fatty acid methyl esters, residual BMP and partially hydrolysed LPG contaminants were removed by butanol–methanol extraction, and the aqueous phase containing GPGs was collected and dried under nitrogen. The dried GPGs were resuspended in methanol, insoluble salts were removed by paper filtration and the filtrate was again dried under nitrogen. Purified GPGs were dissolved in water, aliquoted and stored at −20 °C. Concentrations were determined by LC–MS/MS against a chemically synthesized *R*,*S*-GPG standard. LC–MS/MS analysis confirmed the absence of residual BMP or LPG in all GPG preparations.

### Chemical synthesis of *S*,*S*-C18:1-LPG

*S*,*S*-configured LPG (18:1-(*S*,*S*′)-LPG) was synthesized via a multistep stereospecific route starting from (*R*)-(2,2-dimethyl-1,3-dioxolan-4-yl)methanol. The alcohol was first coupled with oleoyl chloride in dichloromethane containing pyridine and DMAP to yield (*R*)-(2,2-dimethyl-1,3-dioxolan-4-yl)methyl oleate. Subsequent acetal hydrolysis with aqueous acetic acid afforded (*R*)-2,3-dihydroxypropyl oleate, which was protected as the bis-*tert*-butyldimethylsilyl (TBS) ether using TBSCl and imidazole in THF/*N*,*N*-dimethylformamide (DMF). Selective desilylation with pyridinium hydrofluoride produced the mono-TBS derivative, (*R*)-2-((tert-butyldimethylsilyl)oxy)-3-hydroxypropyl oleate.

The free hydroxyl group was phosphitylated using 2-cyanoethyl (((*S*)-2,2-dimethyl-1,3-dioxolan-4-yl)methyl) diisopropylphosphoramidite and 1*H*-tetrazole in THF to generate a phosphite intermediate, which was oxidized with *tert*-butyl hydroperoxide to the corresponding phosphate triester. Removal of the cyanoethyl protecting group with triethylamine yielded the monoester phosphate, and final global deprotection with aqueous acetic acid (50 °C, 2 h) removed both the TBS and acetonide groups to yield 18:1-(*S*,*S*′)-LPG. The product was neutralized with ammonium hydroxide and purified by preparative reverse-phase high-performance liquid chromatography (HPLC; Waters XBridge BEH C18, 10 mM NH_4_HCO_3_/ACN, 20–60% gradient over 15 min).

The final compound was obtained as an off-white gum (25% yield, 97% purity) and confirmed by ^1^H and ^31^P NMR (CD_3_OD) and LC–MS/MS, consistent with the expected structure of 18:1-(*S*,*S*′)-LPG.

### Synthesis of C18:1-LPG acyl isomer standards

*R*,*rac-*LPG (858125, Avanti) was used as the 1-C18:1-LPG standard. The 2-C18:1-LPG standard was generated as previously described^61^. In brief, 1 mg of 18:1–18:1 PG (840475, Avanti) was incubated at 37 °C for 80 min in a reaction mixture containing 78 mM phosphate, 10% diethyl ether, 2 mM CaCl_2_, 0.05% Triton X-100 and 4.5 µg ml^−^^1^ *Rhizomucor miehei* lipase (A225779, Antibodies.com), which exhibits intrinsic PLA_1_ activity (pH 7.4). The reaction was quenched by adding nine volumes of acidic methanol (pH 4.0). Both *R*,*rac*-LPG and enzymatically generated 2-C18:1-LPG were diluted to 1 µM in acidic methanol and analysed in parallel by LC–MS/MS.

### Acyltransferase activity assays

Enzymatic reactions were performed in 25-μl volumes in 1.5-ml microcentrifuge tubes using Hepes buffer (20 mM Hepes (pH 7.4), 150 mM NaCl, 0.05% LMNG). Unless otherwise indicated, reactions contained 3.4 nM CLN8, 100 μM acyl-CoA (substrate A) and 500 μM LPG or GPG (substrate B). When using acyl-CoA mixtures, each was present at 100 μM. Reactions were assembled on ice and incubated at 37 °C for 15 min (or as specified for time-course experiments). Reactions were quenched by adding either 500 μl butanol/methanol (3:1) for TLC or 200 μl 80% ACN with 0.2% FA for LC–MS/MS.

For *K*_m_ determination of *S*,*S*-GPG, acyl-CoA was fixed at 100 μM. Radiolabelling assays used a 10× tracer mix prepared by combining oleoyl-[1-^14^C]-CoA (American Radiochemicals) with unlabelled oleoyl-CoA at a 1:12.5 ratio, yielding a final acyl-CoA concentration of 100 μM in the reaction.

All assays included controls lacking enzyme, substrate A or substrate B, run under identical conditions. For time-course assays, controls were incubated for the longest timepoint; for concentration series, controls contained the highest substrate concentration.

### Lipid extraction

Total lipids were extracted from cells, in vitro assays, animal tissues and zebrafish larvae using the butanol–methanol (BUME) method^62^. Extractions were performed in 2-ml screw-cap plastic tubes (3469-11, Thermo Fisher Scientific), with blank extractions run in parallel to monitor plastic-derived contaminants. For tissues and zebrafish larvae, preweighed frozen samples were homogenized with 1.4-mm ceramic beads (19-645-3, Cole Parmer UK) directly in 0.5 ml ice-cold butanol:methanol (3:1). Subsequently, 0.5 ml 1% acetic acid and 0.5 ml heptane:ethyl acetate (3:1) were added, and samples were vortexed vigorously for 5 min. After centrifugation (6,000*g*, 5 min), the upper organic phase was transferred to glass vials. A second extraction was performed on the remaining aqueous phase, and organic layers were combined. Solvents were evaporated under nitrogen, and dried lipid extracts were stored at −70 °C until analysis.

### GPG extraction and LC–MS/MS quantification

GPG was extracted from mouse tissue by homogenizing samples in 2-ml screw-cap tubes (3469-11, Thermo Fisher Scientific) containing 1 ml 80% methanol and 1.4-mm ceramic beads (19-645-3, Cole Parmer UK). Homogenates were clarified by centrifugation (20,000*g*, 15 min, 4 °C), and the resulting supernatants were transferred to glass vials for analysis.

GPG was analysed by LC–MS/MS using a Shimadzu 8060 triple quadrupole MS (Shimadzu, UK). For initial MS^1^ and product-ion scans, 2 µl of a 100-nM *R*,*S*-GPG standard in methanol was introduced by flow injection, and spectra were acquired over an *m*/*z* range of 100–1,000. Chromatographic separation was performed on an Atlantis Premier BEH Z-HILIC column (1.7 µm, 2.1 × 150 mm; Waters) using mobile phase A (15 mM ammonium acetate with 0.1% ammonium) and mobile phase B (100% methanol). The column was operated at a flow rate of 0.2 ml min^−^^1^, and 2 µl sample was injected.

HPLC was conducted on an LC-30AD system at 0.1 ml min^−^^1^, with samples injected at 0.5 min using a SIL-30AC autosampler. The total run time was 6 min, with the following gradient: 0–1 min, 80% B; 1–3 min, 80% → 20% B; 3–5 min, 20% B; 5–6 min, 80% B. No internal standard was used.

Source parameters were as follows: nebulising gas, 3 L min^−^^1^; heating gas, 10 L min^−^^1^; interface temperature, 300 °C; desolvation line temperature, 245 °C; heat-block temperature, 400 °C; drying gas, 10 L min^−^^1^. GPG was detected in negative-ion mode using the following transitions: *m*/*z* 244.5 → 152.9 (dwell 100 ms; Q1 20 V; CE 15 eV; Q3 20 V), 244.5 → 78.95 (dwell 100 ms; Q1 20 V; CE 35 eV; Q3 20 V) and 244.5 → 170.95 (dwell 100 ms; Q1 20 V; CE 17 eV; Q3 21 V).

### Thin-layer chromatography

Lipids extracted from in vitro assays were dissolved in 25 μl HPLC-grade chloroform and spotted at the base of 10 cm × 10 cm silica TLC plates (821050, Macherey-Nagel). Plates were developed in sealed glass chambers using a solvent system of chloroform:methanol:ammonium hydroxide (65:25:4, v/v) until the solvent front reached ~1 cm from the top. Developed plates were dried under a laminar flow hood. For fluorescence detection, plates were imaged on an Amersham Typhoon imager using the Cy2 fluorescence setting. For phosphorimaging, TLC plates were exposed to pre-erased phosphor screens (GE Healthcare) in light-tight cassettes overnight and then scanned on the Typhoon imager with the phosphor imaging method. The migration of LPG and BMP reaction products was confirmed by *R*,*rac*-LPG (858125, Avanti) and *R*,*S*-BMP (857133, Avanti) standards, visualized by primuline staining and UV detection.

### Targeted LC–MS/MS analysis of lipids

Targeted quantification of LPGs and BMPs was performed on a Xevo TQ-S triple quadrupole MS (Waters) coupled to an I-Class ACQUITY ultra performance liquid chromatography (UPLC) system (Waters). Samples were stored in glass vials at 8 °C before analysis, and 10 µl was injected via autosampler for each run. Chromatographic separation was achieved using a BEH C18 column (1 mm × 50 mm, 1.7 µm particle size, 130 Å pore size; Waters) maintained at 30 °C and fitted with a 0.2-µm UPLC in-line filter.

Mobile phase A consisted of 5% ACN and 0.1% FA, and mobile phase B of 95% ACN and 0.1% FA, delivered at a flow rate of 0.2 ml min^−^^1^ under the following gradient: 0–1.5 min, 5% B; 1.5–3 min, 5–40% B; 3–12 min, 40–70% B; 12–15 min, 70–100% B; and from 15 min to the end of the run, 100% B. The total run time was 20 min when measuring LPG alone or 60 min when measuring both LPG and BMP.

MS detection was carried out in positive ion mode using multiple reaction monitoring with electrospray ionization. The source parameters were as follows: capillary voltage, 3.1 kV; desolvation temperature, 500 °C; and source temperature, 150 °C. Nitrogen served as the curtain gas and argon as the collision gas. Cone voltages, collision energies and analyte-specific MS/MS transitions are presented in Supplementary Table 6. For BMP, both [M + H]^+^ and [M+Na]^+^ adduct peaks could be detected, which facilitated correct peak assignment. Both adducts gave the same qualitative results; however, the [M+Na]^+^-to-[M+Na]^+^ transition^63^ offered increased sensitivity.

Quantification was performed in MassLynx 4.1 software using automatic peak selection followed by manual inspection and correction when required. LPGs and BMPs were quantified against a five-point calibration curve generated from tenfold serial dilutions of *R*,*rac*-LPG (858125, Avanti) and *R*,*S*-BMP (857133, Avanti) standards, analysed alongside each sample batch. Multiple transitions were included for peak validation, as both transitions should have identical retention times.

Under our workflow, BMP acyl positional isomers and stereoisomers were not resolved; therefore, any BMP acyl-position assignments shown in schemes or figures are inferred from previous literature^20^ rather than directly determined by our LC–MS/MS workflow. Similarly, *S*,*S*-18:1-LPG and *R*,*rac*-18:1-LPG could not be distinguished chromatographically, as both produced identical MS signals under our conditions (Extended Data Fig. 9d). However, LPG acyl-positional isomers were resolved under our standard LC–MS/MS conditions, consistently appearing as two distinct peaks separated by 0.2–0.5 min in retention time. These peaks corresponded to the 2-acyl- and 1-acyl-LPG positional isomers, with the 2-acyl species eluting earlier under reverse-phase conditions^64^. In all CLN8 in vitro assays, only the first (2-acyl) LPG peak was detected and quantified; for the C18:1 species, the identity of this peak was confirmed using 1-acyl and 2-acyl C18:1-LPG standards. In biological samples, where appropriate, the integrated areas of both positional acyl isomers were combined to represent the total abundance of the corresponding LPG species.

### Statistics and reproducibility

No statistical methods were used to predetermine sample sizes. Sample sizes were selected on the basis of previous experience and were similar to those used in comparable published studies^43^. The number of biological replicates or animals used in each experiment is stated in the corresponding figure legend (Figs. 1–4 and Extended Data Figs. 1–3 and 7–9. For animal experiments, the species, strain, sex, age and number of animals are provided in the relevant subsection of the Methods and figure legend.

Unless otherwise stated, data are presented as the mean ± s.e.m., with individual data points shown. Where applicable, values are expressed as fold changes relative to the indicated control, as specified in the figure legends. For clarity of presentation, data from males and females were pooled in some animal experiments because no sex-dependent effects were observed; male and female animals are nevertheless distinguished by different symbol shapes in the figures. Analysing the data with sex included as a covariate produced comparable results and did not alter the statistical conclusions.

Statistical analyses were performed using GraphPad Prism version 10.2. The statistical test used for each experiment is specified in the corresponding figure legend. Comparisons between two groups were performed using two-tailed Student’s *t*-tests. Comparisons involving more than two groups were performed using one-way analysis of variance (ANOVA). Where experiments involved two independent variables, two-way ANOVA was used to assess the main effects and their interaction, followed by Sidak’s or Dunnett’s multiple-comparisons test, as indicated. Data distribution was assumed to be normal but was not formally tested. Exact *P* values are reported where applicable.

No data were excluded from the analyses. Mice were randomly assigned to experimental groups while maintaining a balanced distribution of males and females between groups. Mouse tissue analyses were performed with investigators blinded to group allocation.

Each data point from an in vitro biochemical or enzymatic assay represents an individual measurement, unless otherwise stated. All in vitro experiments were independently repeated at least once using a separate batch of purified protein. Separate protein batches were generated from the same stable cell line cultured and purified on different occasions and were therefore considered biological replicates. Independent experiments produced consistent results; where results from a representative experiment are shown, this is stated in the corresponding figure legend.

Graphs and enzyme kinetic analyses were prepared using GraphPad Prism version 10.2. Figures were assembled and edited using Affinity Publisher version 2.4.2.

### Reporting summary

Further information on research design is available in the Nature Portfolio Reporting Summary linked to this article.

## Online content

Any methods, additional references, Nature Portfolio reporting summaries, source data, extended data, supplementary information, acknowledgements, peer review information; details of author contributions and competing interests; and statements of data and code availability are available at https://doi.org/10.1038/s41556-026-02061-0.

## Supplementary information

Reporting Summary

Peer Review File

Supplementary Tables 1–6 Supplementary Table 1. Cryo-EM data collection, refinement and validation statistics. Supplementary Table 2. Solvent-accessible cavity volumes around the catalytic histidine residues of CLN8 and related structural homologues. Supplementary Table 3. Details of antibodies used in this study. Supplementary Table 4. Details of chemicals and reagents used in this study. Supplementary Table 5. Details of plasmids used in this study. Supplementary Table 6. MS/MS transitions (positive mode), cone voltages and collision energies used in this study.

## Source data

Source Data Fig. 1 Statistical source data.

Source Data Fig. 2 Statistical source data.

Source Data Fig. 3 Statistical source data.

Source Data Fig. 4 Statistical source data.

Source Data Extended Data Fig. 1 Statistical source data.

Source Data Extended Data Fig. 2 Statistical source data.

Source Data Extended Data Fig. 3 Statistical source data.

Source Data Extended Data Fig. 7 Statistical source data.

Source Data Extended Data Fig. 9 Statistical source data.

Unprocessed gels and blots Unprocessed scans, gels and blots for all relevant figures.

## Data Availability

Coordinates and cryo-EM maps have been deposited in the Protein Data Bank (PDB) and Electron Microscopy Data Bank (EMDB), with the following accession numbers: apo-CLN8 (PDB: 9THK; and EMD: 55929), CLN8 + C18:1-CoA (PDB: 9TKA; and EMD: 56022), CLN8 + C22:6-CoA (PDB: 9TK9; and EMD: 56021). Requests for reagents or biological materials should be directed to the lead author (kp416@cam.ac.uk). Source data are provided for Figs. 1–4 and Extended Data Figs. 1–3, 7 and 9. All other data supporting the findings of this study are available from the corresponding author on reasonable request.
