# Supplementary material for: The Batten disease protein CLN8 is a stereospecific acyltransferase in bis(monoacylglycero)phosphate biosynthesis
Source: Nat Cell Biol. 2026 Sep 15;28(10):2068–77. doi: 10.1038/s41556-026-02061-0 (PMC13661276; doi:10.1038/s41556-026-02061-0)

Fig. 1h (TLC autoradiogram)

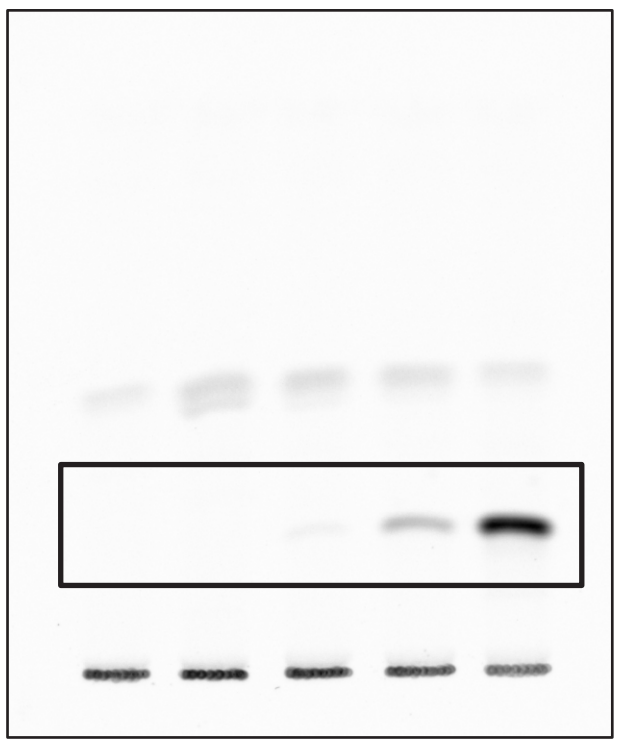

Fig. 2d (TLC fluorescence)

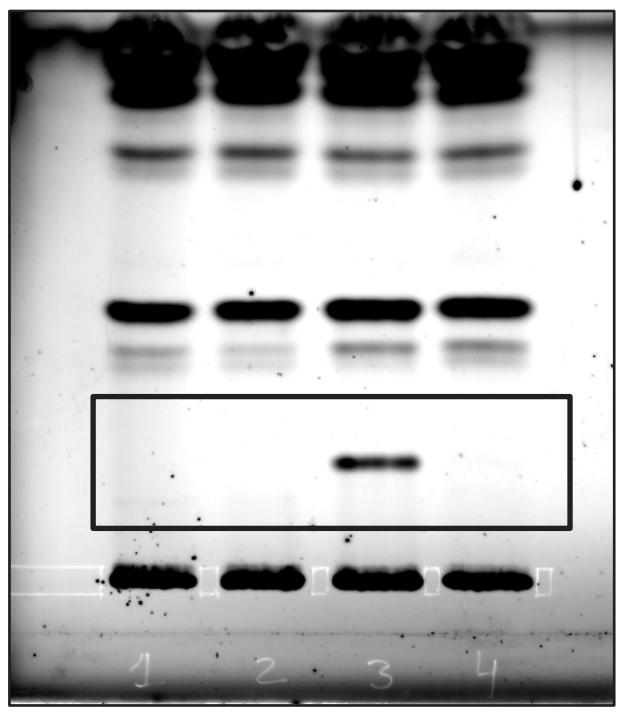

Fig. 2e

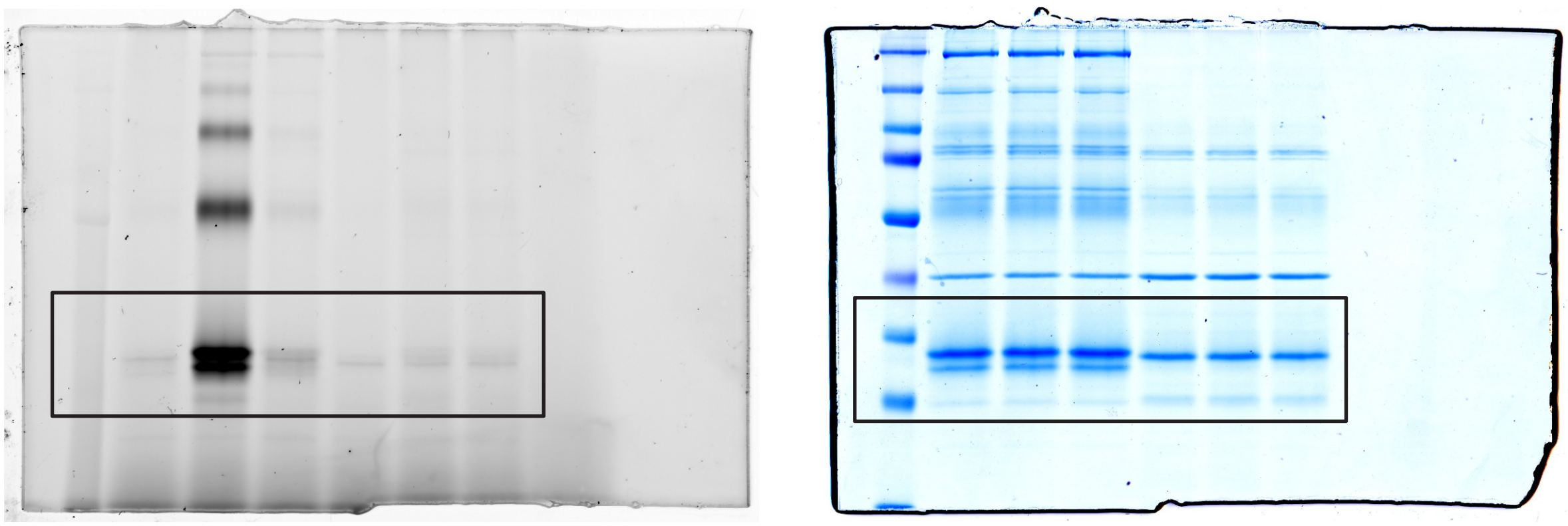

Extended Data Fig. 1c

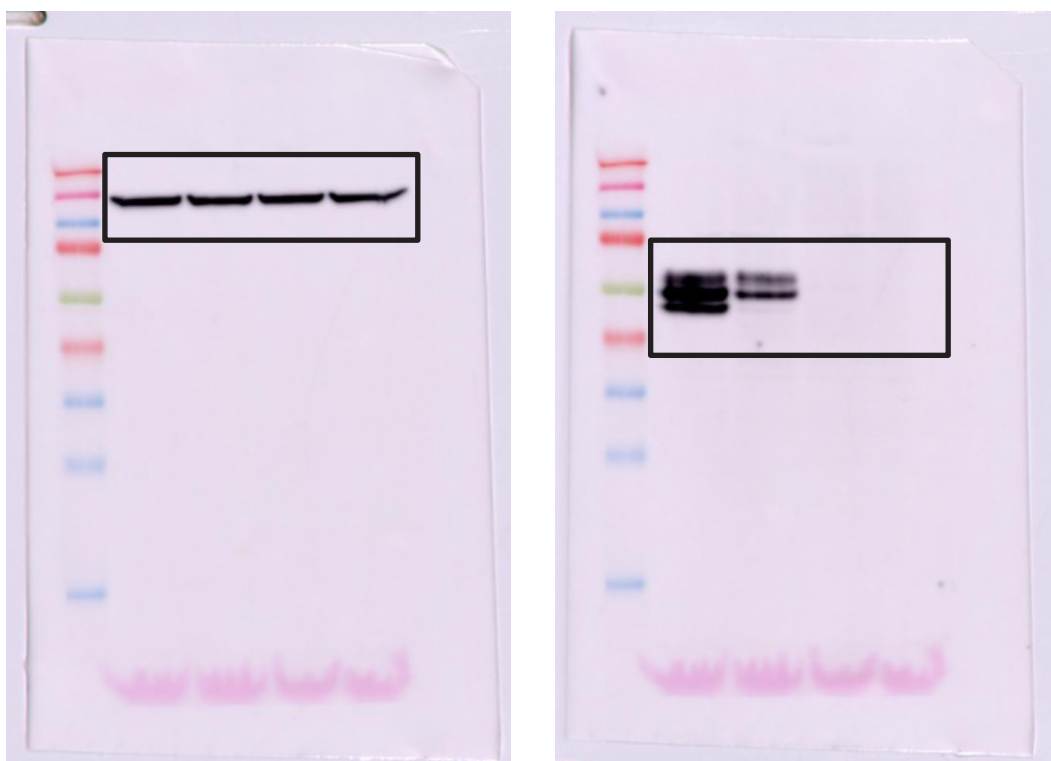

Extended Data Fig. 1e

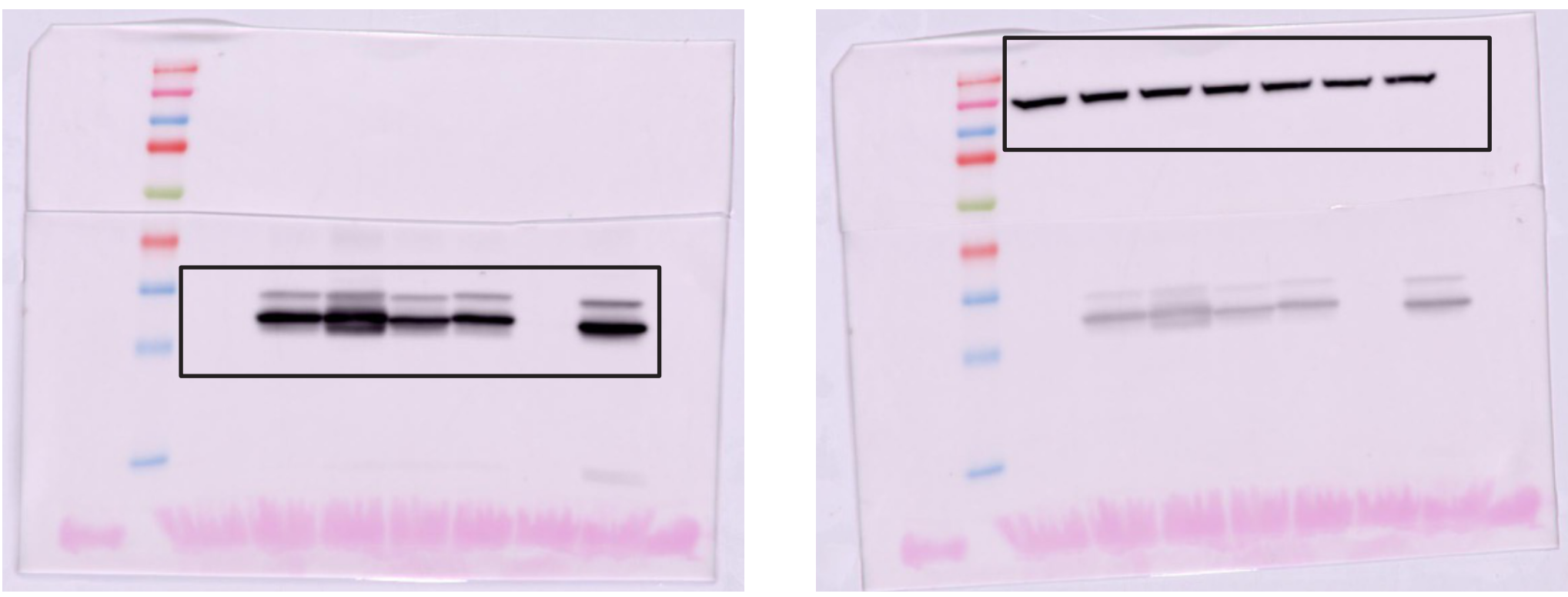

Extended Data Fig. 2a-b

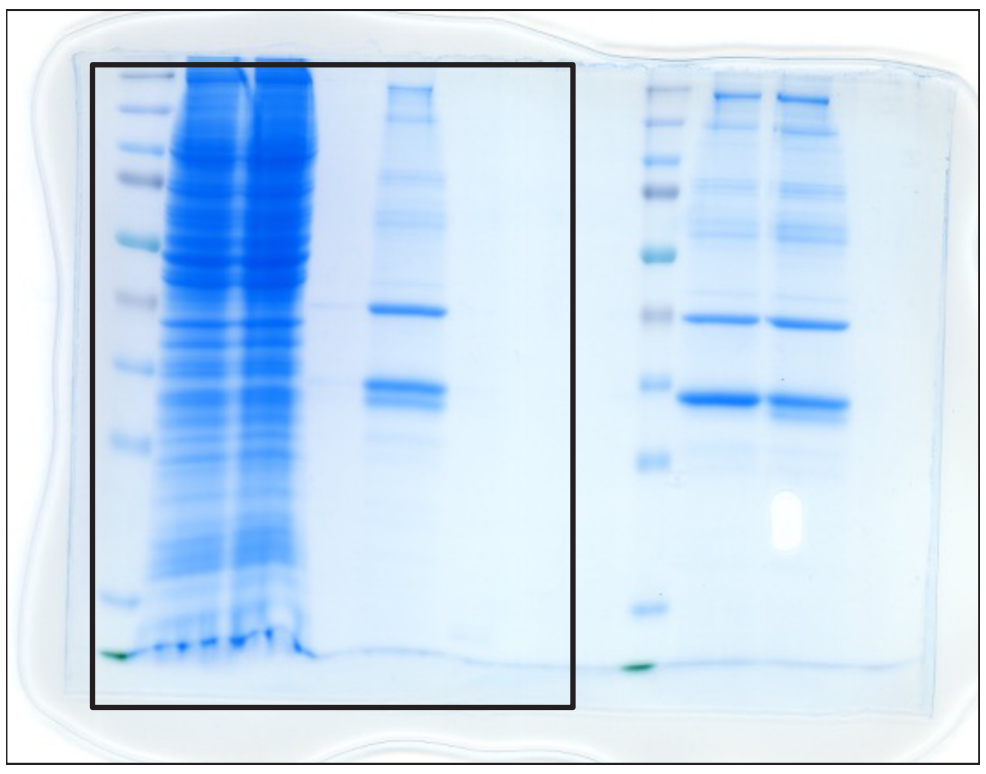

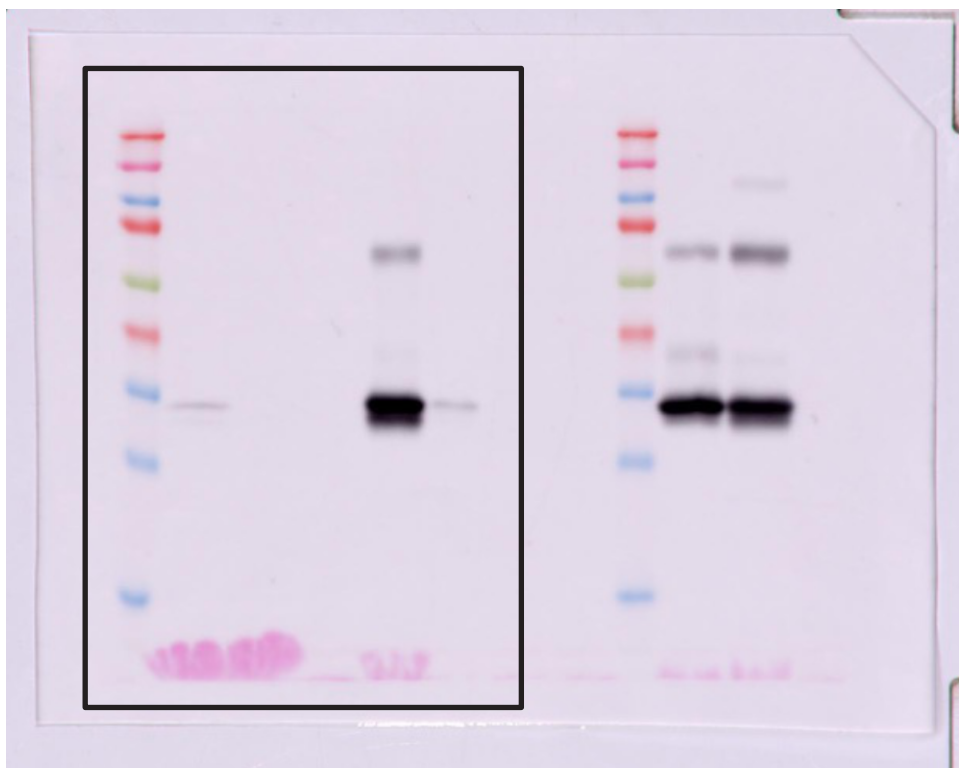

Extended Data Fig. 3a-b

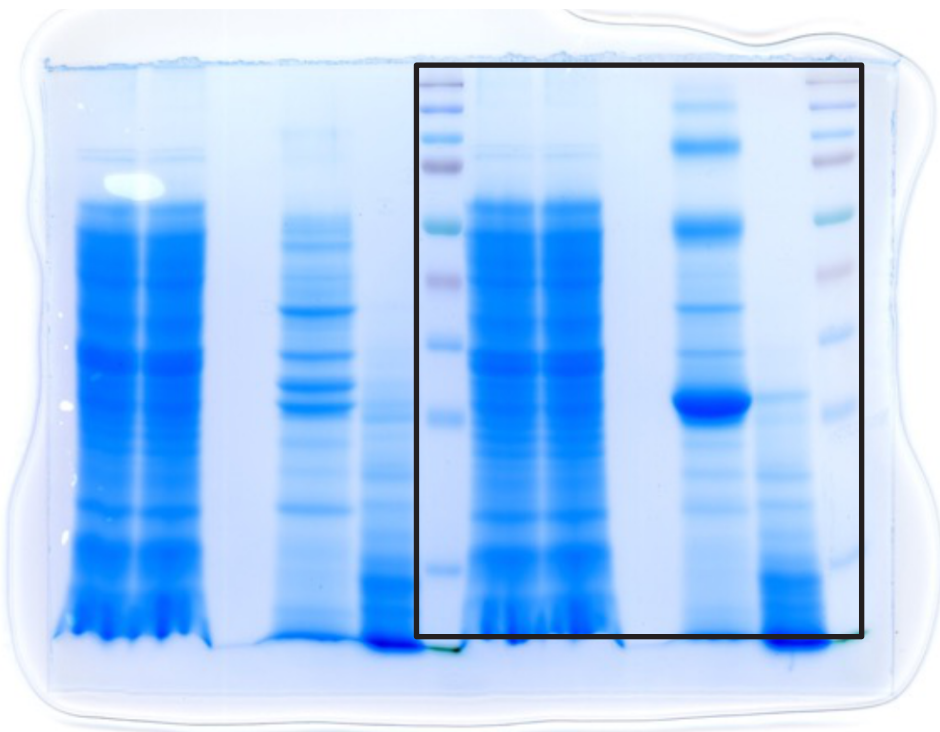

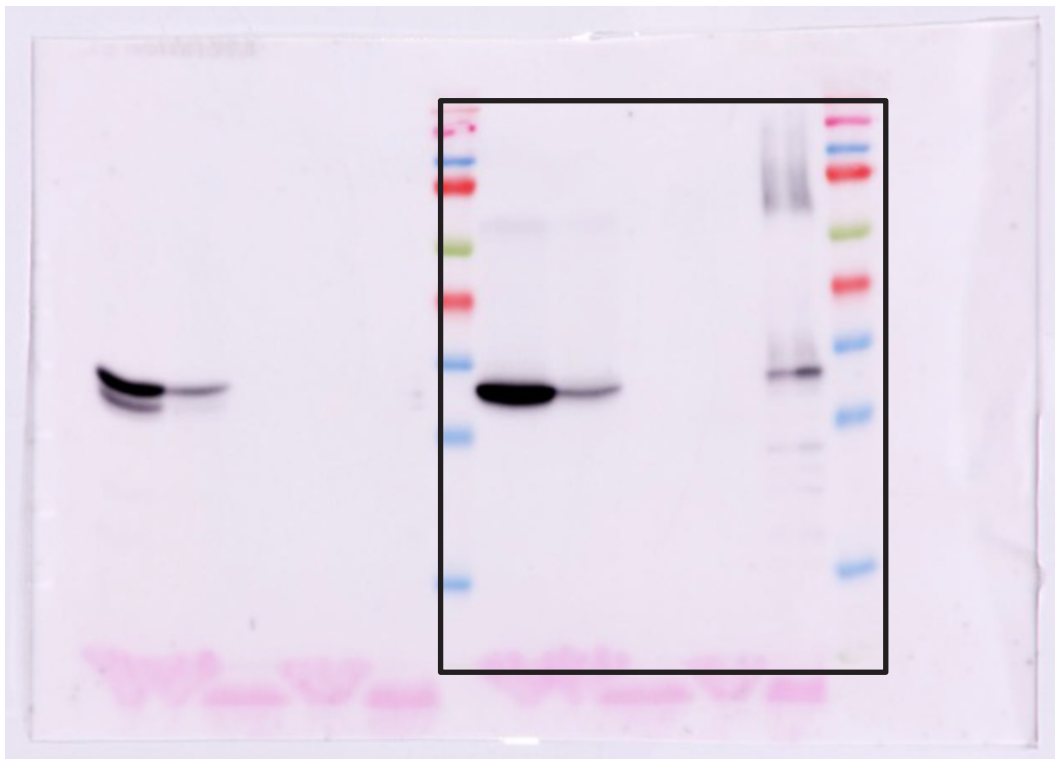

Extended Data Fig. 3d

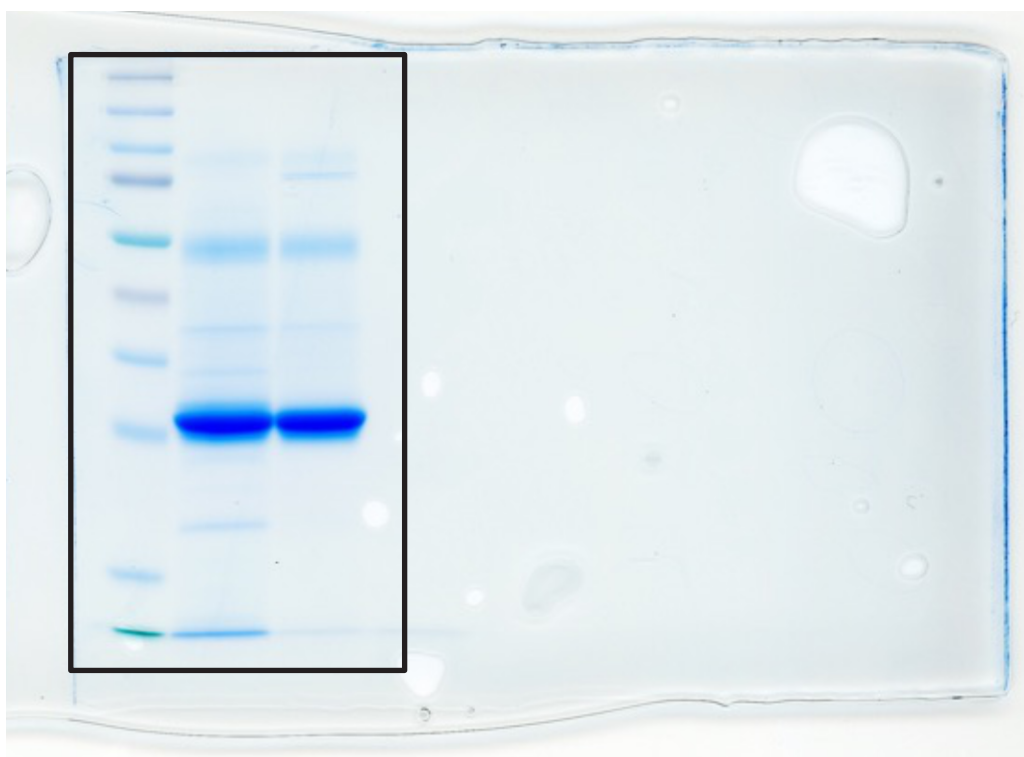

Extended Data Fig. 7a

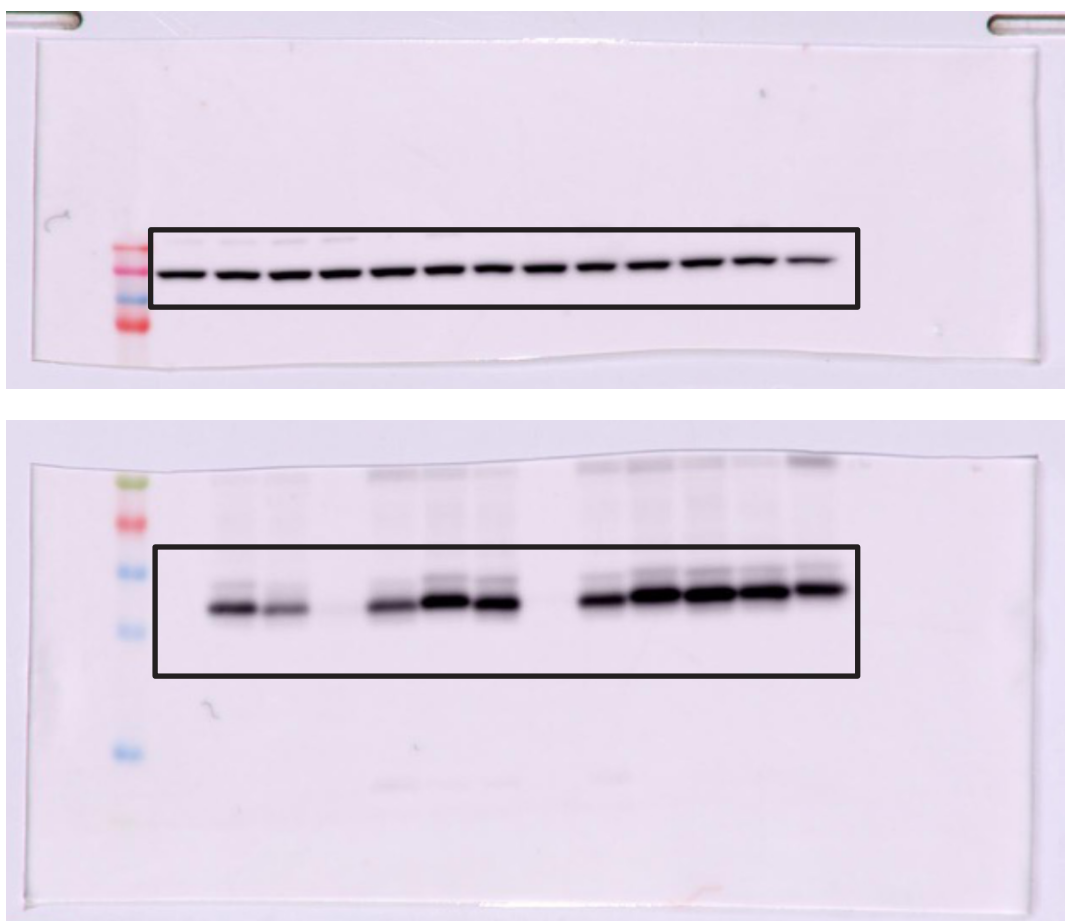

Extended Data Fig. 8a-b

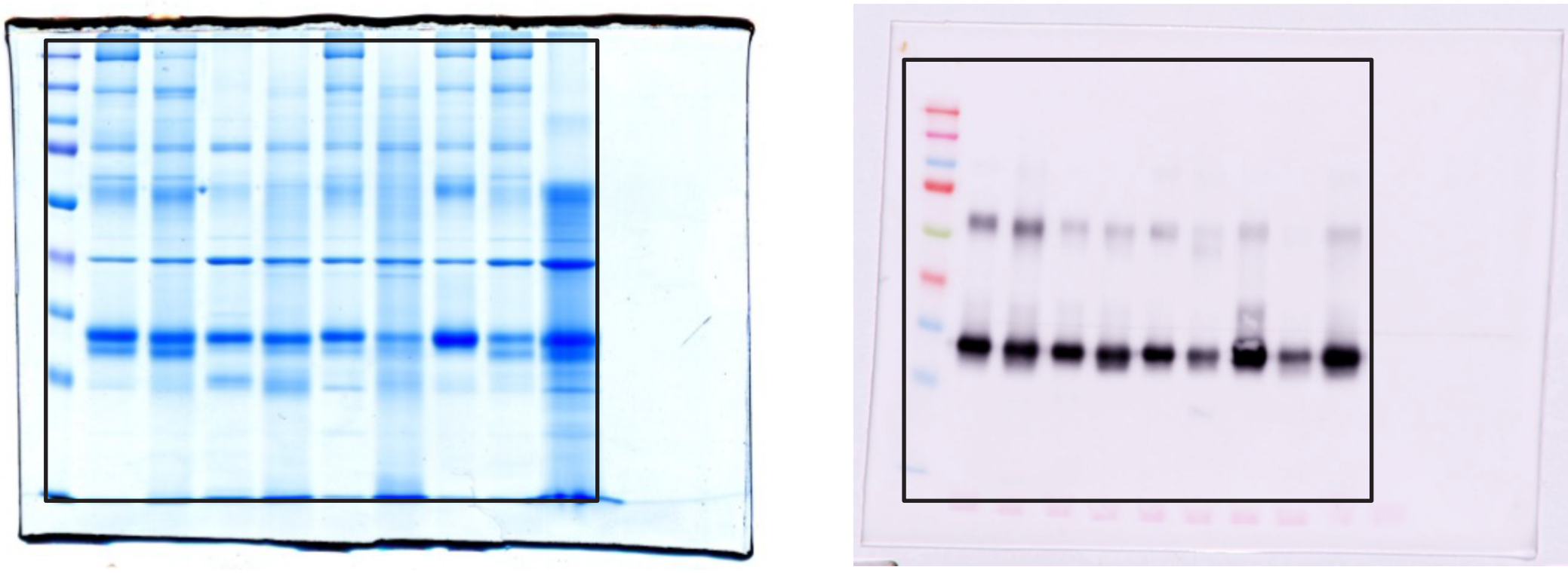

Extended Data Fig. 8c (TLC autoradiogram)

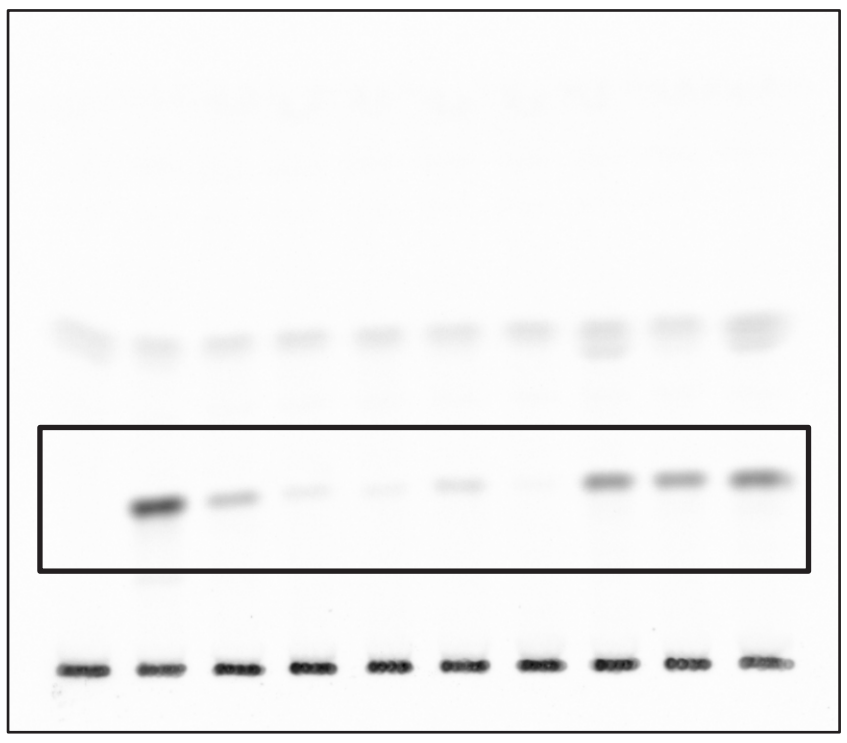

Extended Data Fig. 8d

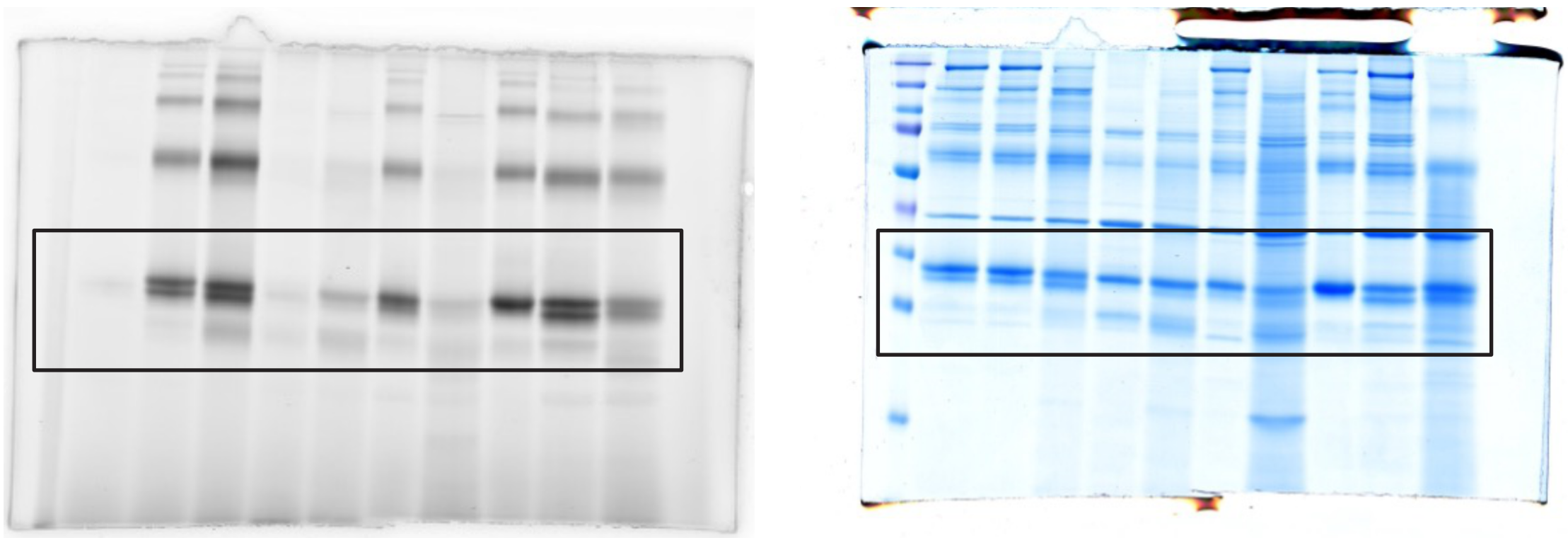

Extended Data Fig. 8e

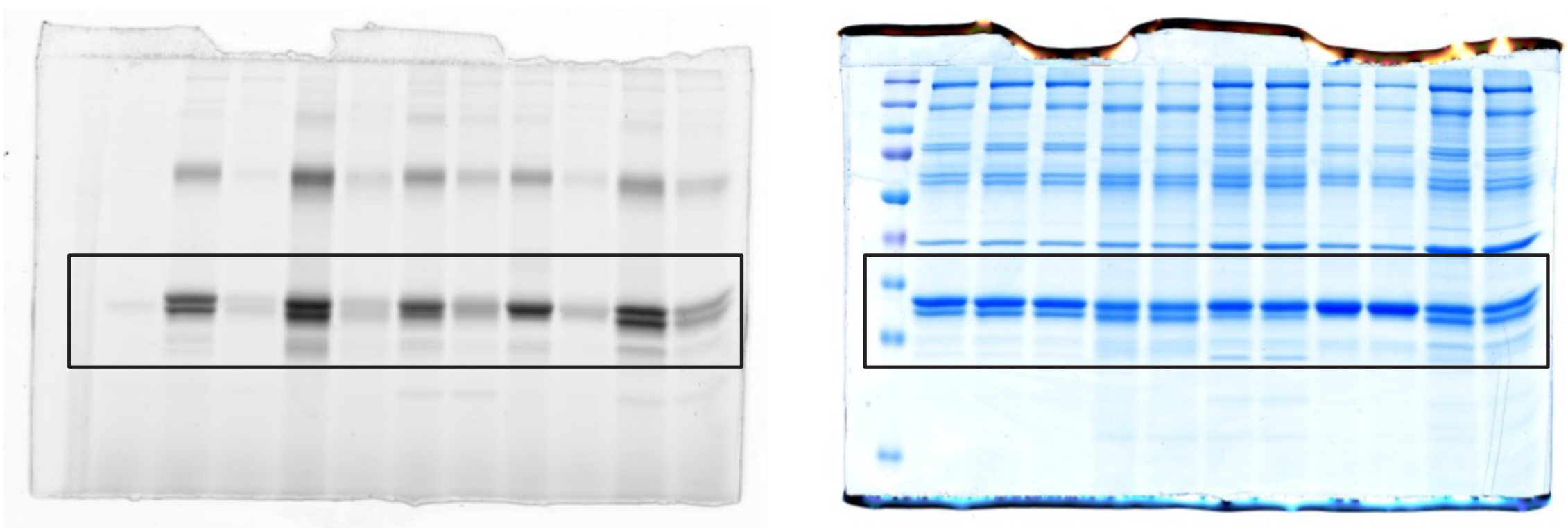

Supplement: Supplementary file 1 [file 41556_2026_2061_MOESM13_ESM.pdf]
