# Supplementary material for: The Batten disease protein CLN8 is a stereospecific acyltransferase in bis(monoacylglycero)phosphate biosynthesis
Source: Nat Cell Biol. 2026 Sep 15;28(10):2068–77. doi: 10.1038/s41556-026-02061-0 (PMC13661276; doi:10.1038/s41556-026-02061-0)
Supplement: Supplementary file 2 [file 41556_2026_2061_MOESM1_ESM.pdf]

nature portfolio

Corresponding author(s): Kasparas Petkevicius

Last updated by author(s): Jul 20, 2026

Reporting Summary

Nature Portfolio wishes to improve the reproducibility of the work that we publish. This form provides structure for consistency and transparency in reporting. For further information on Nature Portfolio policies, see our Editorial Policies and the Editorial Policy Checklist.

Statistics

For all statistical analyses, confirm that the following items are present in the figure legend, table legend, main text, or Methods section.

n/a	Confirmed	
☐	☒	The exact sample size (*n*) for each experimental group/condition, given as a discrete number and unit of measurement
☐	☒	A statement on whether measurements were taken from distinct samples or whether the same sample was measured repeatedly
☐	☒	The statistical test(s) used AND whether they are one- or two-sided<br>*Only common tests should be described solely by name; describe more complex techniques in the Methods section.*
☐	☒	A description of all covariates tested
☐	☒	A description of any assumptions or corrections, such as tests of normality and adjustment for multiple comparisons
☐	☒	A full description of the statistical parameters including central tendency (e.g. means) or other basic estimates (e.g. regression coefficient) AND variation (e.g. standard deviation) or associated estimates of uncertainty (e.g. confidence intervals)
☐	☒	For null hypothesis testing, the test statistic (e.g. F, t, r) with confidence intervals, effect sizes, degrees of freedom and P value noted<br>*Give P values as exact values whenever suitable.*
☒	☐	For Bayesian analysis, information on the choice of priors and Markov chain Monte Carlo settings
☒	☐	For hierarchical and complex designs, identification of the appropriate level for tests and full reporting of outcomes
☒	☐	Estimates of effect sizes (e.g. Cohen's *d*, Pearson's *r*), indicating how they were calculated

Our web collection on statistics for biologists contains articles on many of the points above.

Software and code

Policy information about availability of computer code

Data collection	Cryo-EM movies were recorded using EPU v3.1 (ThermoFisher). Zebrafish locomotor behaviour was acquired using the DanioVision system with EthoVision XT automated tracking. Targeted lipid LC-MS/MS data acquisition was done with MassLynx 4.1 (Waters).
Data analysis	Cryo-EM analysis: Warp — https://github.com/cramerlab/warp ; cryoSPARC 3.3.2; UCSF ChimeraX 1.7.1; ISOLDE 1.10; ChemEM v0.0.6; MolProbity 4.5.2; Servalcat; PyMOL 3.1.6.1 (Schrödinger, LLC). Targeted lipid LC-MS/MS quantification was performed in MassLynx 4.1 (Waters). Proteomics DIA data were processed in DIA-NN v2.0. Statistics/graphing/kinetics were performed in GraphPad Prism v10.2, and figures were assembled in Affinity Publisher v2.4.2. No custom code was developed for this study.

For manuscripts utilizing custom algorithms or software that are central to the research but not yet described in published literature, software must be made available to editors and reviewers. We strongly encourage code deposition in a community repository (e.g. GitHub). See the Nature Portfolio guidelines for submitting code & software for further information.

Data

Policy information about availability of data

All manuscripts must include a data availability statement. This statement should provide the following information, where applicable:

- Accession codes, unique identifiers, or web links for publicly available datasets
- A description of any restrictions on data availability
- For clinical datasets or third party data, please ensure that the statement adheres to our policy

Coordinates and cryo-EM maps have been deposited in the PDB and EMDB, with the following accession numbers: apo-CLN8 (PDB: 9THK; and EMD: 55929), CLN8 + C18:1-CoA (PDB: 9TKA; and EMD: 56022), CLN8 + C22:6-CoA (PDB: 9TK9 and EMD: 56021). Source data have been provided in Source Data. All other data supporting the findings of this study are available from the corresponding author on reasonable request. Requests for reagents or biological materials should be directed to the lead author (kp416@cam.ac.uk).

Research involving human participants, their data, or biological material

Policy information about studies with human participants or human data. See also policy information about sex, gender (identity/presentation), and sexual orientation and race, ethnicity and racism.

Reporting on sex and gender	n/a
Reporting on race, ethnicity, or other socially relevant groupings	n/a
Population characteristics	n/a
Recruitment	n/a
Ethics oversight	n/a

Note that full information on the approval of the study protocol must also be provided in the manuscript.

Field-specific reporting

Please select the one below that is the best fit for your research. If you are not sure, read the appropriate sections before making your selection.

☒ Life sciences ☐ Behavioural & social sciences ☐ Ecological, evolutionary & environmental sciences

For a reference copy of the document with all sections, see nature.com/documents/nr-reporting-summary-flat.pdf

Life sciences study design

All studies must disclose on these points even when the disclosure is negative.

Sample size	No statistical method was used to predetermine sample sizes. Sample sizes were based on prior experience with the relevant assays, comparable published studies and the availability of animals of the required genotype, age and sex. The precise sample size and experimental unit are stated in each figure legend. Cell-based quantitative comparisons generally used three independent biological replicates. In vitro biochemical assays used assay replicates within an experiment and were independently repeated with at least two separately prepared protein batches. Mouse studies used individual animals as biological replicates. Zebrafish locomotor assays combined three independent experiments, and electrophysiology used eight larvae per group. Sample sizes were not selected on the basis of post hoc statistical significance.
Data exclusions	No data, animals or experimental replicates were excluded from the analyses. No pre-established exclusion criteria were applied.
Replication	Cell-based experiments were independently repeated at least twice and yielded consistent results. In vitro biochemical and enzymatic assays were independently repeated using at least two separate protein preparations; where plotted points are technical assay replicates, this is identified in the figure legend. Zebrafish locomotor measurements were obtained across three independent experiments. Mouse studies were performed using individual animals as biological replicates in the cohort sizes stated in the figure legends. Cryo-EM structures were determined from one dataset per biochemical condition and were supported by independent biochemical experiments. All attempted experimental replications were successful.
Randomization	Mice in the LPG rescue experiment were randomly allocated to treatment groups while balancing sex. Genotype-comparison cohorts were defined by genotype and therefore could not be randomly allocated; age- and sex-matched or littermate wild-type controls were used. Targeted LC-MS/MS acquisition order was randomized where feasible. Cell and biochemical groups were defined by genotype, construct or reaction condition and were processed in parallel using identical workflows. Zebrafish larvae were randomly allocated to treatment groups.
Blinding	Mouse tissue analyses were performed with investigators blinded to genotype/treatment allocation. Other experiments were not blinded

Blinding	because the primary readouts were quantitative or automated and all groups were processed identically. Zebrafish locomotion was quantified by automated tracking. Local-field-potential recordings and event analysis were blinded.

Reporting for specific materials, systems and methods

We require information from authors about some types of materials, experimental systems and methods used in many studies. Here, indicate whether each material, system or method listed is relevant to your study. If you are not sure if a list item applies to your research, read the appropriate section before selecting a response.

Materials & experimental systems

n/a	Involved in the study
☐	☒ Antibodies
☐	☒ Eukaryotic cell lines
☒	☐ Palaeontology and archaeology
☐	☒ Animals and other organisms
☒	☐ Clinical data
☒	☐ Dual use research of concern
☒	☐ Plants

Methods

n/a	Involved in the study
☒	☐ ChIP-seq
☒	☐ Flow cytometry
☒	☐ MRI-based neuroimaging

Antibodies

Antibodies used	Primary antibodies were rabbit anti-CLN5 (Abcam, cat. no. ab170899, RRID AB_3662651), mouse anti-vinculin (Merck/Sigma-Aldrich, cat. no. V9131, RRID AB_477629), mouse monoclonal anti-FLAG M2 (Sigma-Aldrich, cat. no. F1804, RRID AB_262044), rabbit polyclonal anti-HA tag SG77 (Thermo Fisher Scientific, cat. no. 71-5500, RRID AB_2533988), and HRP-conjugated anti-6×His (Abcam, cat. no. ab1187, RRID AB_298652). All primary antibodies were used at a 1:1,000 dilution and incubated in a cold room for 16–18 h. Secondary antibodies were HRP-conjugated anti-mouse IgG (Cell Signaling Technology, cat. no. 7076-S, RRID AB_330924) and HRP-conjugated anti-rabbit IgG (Cell Signaling Technology, cat. no. 7074-S, RRID AB_2099233). Secondary antibodies were used at a 1:5,000 dilution for 1 h at room temperature.
Validation	The anti-CLN5 antibody was validated by loss of the expected CLN5 signal in CLN5-knockout cells (Extended Data Fig. 1c), consistent with the manufacturer's knockout validation. Anti-HA, anti-FLAG and anti-6×His antibodies detected proteins only in samples expressing the corresponding epitope-tagged constructs and not in the relevant negative controls, at the expected apparent molecular masses. The anti-vinculin antibody detected a band at the expected molecular mass and was used as a loading control. Manufacturer validation information and catalogue/RRID identifiers are provided above.

Eukaryotic cell lines

Policy information about cell lines and Sex and Gender in Research

Cell line source(s)	HeLa (ECACC 93021013) cell line was obtained from Culture Collections, UK Health Security Agency. The Flp-In T-REx 293 cell line was purchased from Thermo Fisher Scientific (R78007). Saccharomyces cerevisiae strain W303.1B was acquired from ATCC (201238).
Authentication	Human cell lines were authenticated by STR profiling after purchase and again following establishment of in-house master cell banks. Aliquots from these authenticated banks were used for all experiments and to generate derivative cell lines.
Mycoplasma contamination	All human cell lines were routinely tested for mycoplasma contamination and were negative.
Commonly misidentified lines (See ICLAC register)	n/a

Animals and other research organisms

Policy information about studies involving animals; ARRIVE guidelines recommended for reporting animal research, and Sex and Gender in Research

Laboratory animals	Mice (Mus musculus) and zebrafish (Danio rerio) were used. Total BMP analyses used wild-type and homozygous Cln8 mnd/mnd mice on a C57BL/6J background, aged 6–7 months, with four females and four males per genotype. Cln8 R24G/R24G knock-in analyses used 8-week-old wild-type and homozygous mice on a C57BL/6J background, with five females and two males per genotype. The LPG rescue study used 10-week-old Cln8 mnd/mnd mice and wild-type littermates: WT+BSA, two females and two males; Cln8 mnd/mnd + BSA, two females and one male; Cln8 mnd/mnd + R,rac-LPG, two females and two males; and Cln8 mnd/mnd + S,S-LPG, five females and five males. Zebrafish experiments used a previously characterised cln8−/− line; larvae were analysed at 120 hours post-fertilisation. Mouse and zebrafish housing conditions, diet, light cycle and environmental parameters are reported in the Methods.
Wild animals	No wild animals were used.

Reporting on sex	Both female and male mice were included. Sex distributions are stated in the relevant figure legends and individual data points are distinguished by symbol shape where applicable. Where formally tested, no significant sex-dependent effect was detected, so sexes were pooled for the principal genotype/treatment comparisons. Zebrafish larvae were analysed at 120 hours post-fertilisation and were not sexed because sex cannot be determined at this developmental stage.
Field-collected samples	n/a
Ethics oversight	Mouse studies at Sanford Research were approved by the Sanford Research Institutional Animal Care and Use Committee (USDA licence 46-R-0009; protocol 2023-0102). Cln8 R24G mouse studies were authorised by the Project Authorisation Board at the State Provincial Office of Southern Finland (ESAVI/33743/2019 and ESAVI/16903/2024). Mouse LPG rescue studies were approved by the University of Cambridge Animal Welfare and Ethical Review Body and performed under UK Home Office Project Licence PP1740969 in accordance with the Animals (Scientific Procedures) Act 1986. Zebrafish procedures were approved by the Italian Ministry of Health (authorisation 1090/2024-PR) under the oversight of the IRCCS Fondazione Stella Maris Animal Care and Use Committee and in accordance with EU Directive 2010/63/EU.

Note that full information on the approval of the study protocol must also be provided in the manuscript.

Plants

Seed stocks	n/a
Novel plant genotypes	n/a
Authentication	n/a
