# Supplementary material for: The Batten disease protein CLN8 is a stereospecific acyltransferase in bis(monoacylglycero)phosphate biosynthesis
Source: Nat Cell Biol. 2026 Sep 15;28(10):2068–77. doi: 10.1038/s41556-026-02061-0 (PMC13661276; doi:10.1038/s41556-026-02061-0)
Supplement: Supplementary file 3 [file 41556_2026_2061_MOESM2_ESM.pdf]

nature portfolio

Peer Review File

The Batten disease protein CLN8 is a stereospecific acyltransferase in bis(monoacylglycero)phosphate biosynthesis

Corresponding Author: Dr Kasparas Petkevicius

Parts of this Peer Review File have been redacted as indicated to maintain the confidentiality of unpublished data.

Version 0:

Decision Letter:

*Please delete the link to your author homepage if you wish to forward this email to co-authors.

Dear Dr Petkevicius,

Thank you again for submitting your manuscript, "Stereospecific GPG acylation by CLN8 drives BMP biosynthesis and its loss leads to Batten disease", to Nature Cell Biology.

I apologize for the delay in sharing our decision. I'm writing to let you know that we have decided to send your manuscript for peer review, but several points require your attention before we can proceed with peer review.

I am re-opening the manuscript submission system for you to resubmit your manuscript with all associated files needed for peer review directly, within ~2 business days if possible. Please follow the link at the bottom of this email to upload the documents listed below. If you have any issues, please reach out to us before completing the submission.

1. STRUCTURAL DATA:
We require official wwPDB validation reports for newly described atomic structures per journal policy. We also request that authors provide cryo-EM maps, half-maps and models, as well as maps and models obtained from subtomogram averaging if it applies to the work, to help the reviewers in assessing the work. We recommend the use of figshare in our system, which allows for provision of anonymous access links for the referees (https://www.springernature.com/gp/authors/research-data/figshare-integration). Alternatively, please upload .zip folders directly with the submission.
Manuscripts reporting new structures should contain a table summarizing structural and refinement statistics. Please include these tables for new cryo-EM or -ET (https://www.nature.com/documents/nr-tables-cryo-em.doc and modifying this file for ET), NMR (https://www.nature.com/documents/nr-tables-nmr.doc) and X-ray crystallography (https://www.nature.com/documents/nr-tables-xray.doc). To facilitate assessment of the quality of the structural data, a stereo image of a portion of the electron density map (for crystallography papers) or of the superimposed lowest energy structures (>10; for NMR papers) should be provided with the submitted manuscript. If the reported structure represents a novel overall fold, a stereo image of the entire structure (as a backbone trace) should also be provided. For cryo-EM structures, a representative micrograph showing individual particles should be provided in the figures alongside a processing workflow (please see our colleagues at NSMB's Editorial for more information: https://www.nature.com/articles/s41594-025-01567-9).

2. REPORTING SUMMARY:
We want to ensure that the methods and statistics reporting in our papers are of the highest quality. To that end, we ask authors to fill out a Reporting Summary that collects information on experimental design and reagents, as well as an editorial Policy Checklist, which confirms compliance with our editorial policies, including the declaration of Competing Interests. If your paper includes ChIP-seq or flow cytometry data, we ask that you take special care to complete those sections of the Reporting Summary as this data will aid greatly in the review of your manuscript.

Reporting Summary:
https://www.nature.com/documents/nr-reporting-summary.pdf

3- PROTEOMICS AND SEQUENCING:
In order for us to proceed with peer review, please provide accession numbers and reviewer tokens to access sequencing or proteomics datasets if any unpublished datasets are part of your study. Please add this information to your manuscript file. Proteomics data should be deposited to any ProteomeXchange member repository and DNA sequencing, RNA sequencing, genome assembly data should be deposited to any INSDC member repository or Genome Sequence Archive.

4- CODE, MD SIMULATIONS, AND MACHINE LEARNING REPORTING:
Please provide the code used to analyse the data if newly developed (unpublished) code was used in the work. For the reviewers to evaluate the work adequately, they must be able to test the software/review the code themselves. If you have not yet provided the software, we therefore request that you provide a single compressed zip file containing the software with a readme.txt file or other user manual containing complete instructions for installing and running the software. If appropriate, please provide example data and expected output. Sufficient material should be provided for referees to directly test the performance of the software/algorithm. Alternatively, you can upload the code to GitHub and provide the link directly in the manuscript.

Please fill out and return to me the code and software submission checklist that will be made available to editors and reviewers. This form is a dynamic 'smart pdf' and must therefore be downloaded and completed in Adobe Reader, instead of opening it in a web browser.
https://www.nature.com/documents/nr-software-policy.pdf

If molecular dynamics (MD) simulations were performed, please refer to this Editorial and provide a completed MD simulations checklist with your revision: https://www.nature.com/articles/s42003-023-04653-0

Lastly, if it applies, please complete and return the completed machine learning checklist with your submission for re-review:
https://www.nature.com/documents/machine-learning-checklist.pdf

Please use the link below to submit the files. **Please also remember to move forward all other files associated with this version of the paper.**

Link Redacted

*This url links to your confidential home page and associated information about manuscripts you may have submitted or be reviewing for us. If you wish to forward this email to co-authors, please delete the link to your homepage.

Note: This URL links to your confidential home page and associated information about manuscripts you may have submitted, or that you are reviewing for us. If you wish to forward this email to co-authors, please delete the link to your homepage.

We look forward to seeing the revised manuscript and thank you for the opportunity to review your work.

Best wishes,

Melina

Melina Casadio, PhD
Senior Editor, Nature Cell Biology
ORCID ID: https://orcid.org/0000-0003-2389-2243

GUIDELINES FOR SUBMISSION OF NATURE CELL BIOLOGY ARTICLES

READABILITY OF MANUSCRIPTS – Nature Cell Biology is read by cell biologists from diverse backgrounds, many of whom are not native English speakers. Authors should aim to communicate their findings clearly, explaining technical jargon that might be unfamiliar to non-specialists, and avoiding non-standard abbreviations. Titles and abstracts should concisely communicate the main findings of the study, and the background, rationale, results and conclusions should be clearly explained in the manuscript in a manner accessible to a broad cell biology audience. Nature Cell Biology uses British spelling.

ARTICLE FORMAT

TITLE – should be no more than 100 characters including spaces, without punctuation and avoiding technical terms, abbreviations, and active verbs..

AUTHOR NAMES – should be given in full.

AUTHOR AFFILIATIONS – should be denoted with numerical superscripts (not symbols) preceding the names. Full addresses should be included, with US states in full and providing zip/post codes. The corresponding author is denoted by: "Correspondence should be addressed to [initials]."

ABSTRACT – should not exceed 150 words and should be unreferenced. This paragraph is the most visible part of the paper and should briefly outline the background and rationale for the work, and accurately summarize the main results and conclusions. Key genes, proteins and organisms should be specified to ensure discoverability of the paper in online searches.

TEXT – the main text consists of the Introduction, Results, and Discussion sections and must not exceed 3500 words including the abstract. The Introduction should expand on the background relating to the work. The Results should be divided in subsections with subheadings, and should provide a concise and accurate description of the experimental findings. The Discussion should expand on the findings and their implications. All relevant primary literature should be cited, in particular when discussing the background and specific findings.

ACKNOWLEDGEMENTS – should be kept brief. Professional titles and affiliations are unnecessary. Grant numbers can be listed.

AUTHOR CONTRIBUTIONS – must be included after the Acknowledgements, detailing the contributions of each author to the paper (e.g. experimental work, project planning, data analysis etc.). Each author should be listed by his/her initials.

FINANCIAL AND NON-FINANCIAL COMPETING INTERESTS – the authors must include one of three declarations: (1) that they have

no financial and non-financial competing interests; (2) that they have financial and non-financial competing interests; or (3) that they decline to respond, after the Author Contributions section. This statement will be published with the article, and in cases where financial and non-financial competing interests are declared, these will be itemized in a web supplement to the article. For further details please see https://www.nature.com/licenceforms/nrg/competing-interests.pdf.

REFERENCES – are limited to a total of 70 in the main text and Methods combined,. They must be numbered sequentially as they appear in the main text, tables and figure legends and Methods and must follow the precise style of Nature Cell Biology references. References only cited in the Methods should be numbered consecutively following the last reference cited in the main text. References only associated with Supplementary Information (e.g. in supplementary legends) do not count toward the total reference limit and do not need to be cited in numerical continuity with references in the main text. Only published papers can be cited, and each publication cited should be included in the numbered reference list, which should include the manuscript titles. Footnotes are not permitted.

METHODS – Nature Cell Biology publishes methods online. The methods section should be provided as a separate Word document, which will be copyedited and appended to the manuscript PDF, and incorporated within the HTML format of the paper.

Methods should be written concisely, but should contain all elements necessary to allow interpretation and replication of the results. As a guideline, Methods sections typically do not exceed 3,000 words. The Methods should be divided into subsections listing reagents and techniques. When citing previous methods, accurate references should be provided and any alterations should be noted. Information must be provided about: antibody dilutions, company names, catalogue numbers and clone numbers for monoclonal antibodies; sequences of RNAi and cDNA probes/primers or company names and catalogue numbers if reagents are commercial; cell line names, sources and information on cell line identity and authentication. Animal studies and experiments involving human subjects must be reported in detail, identifying the committees approving the protocols. For studies involving human subjects/samples, a statement must be included confirming that informed consent was obtained. Statistical analyses and information on the reproducibility of experimental results should be provided in a section titled “Statistics and Reproducibility”.

All Nature Cell Biology manuscripts submitted on or after March 21 2016, must include a Data availability statement as a separate section after Methods but before references, under the heading "Data Availability”. For Springer Nature policies on data availability see http://www.nature.com/authors/policies/availability.html; for more information on this particular policy see http://www.nature.com/authors/policies/data/data-availability-statements-data-citations.pdf. The Data availability statement should include:

• Accession codes for primary datasets (generated during the study under consideration and designated as "primary accessions") and secondary datasets (published datasets reanalysed during the study under consideration, designated as "referenced accessions"). For primary accessions data should be made public to coincide with publication of the manuscript. A list of data types for which submission to community-endorsed public repositories is mandated (including sequence, structure, microarray, deep sequencing data) can be found here http://www.nature.com/authors/policies/availability.html#data.

• Unique identifiers (accession codes, DOIs or other unique persistent identifier) and hyperlinks for datasets deposited in an approved repository, but for which data deposition is not mandated (see here for details http://www.nature.com/sdata/data-policies/repositories).

• At a minimum, please include a statement confirming that all relevant data are available from the authors, and/or are included with the manuscript (e.g. as source data or supplementary information), listing which data are included (e.g. by figure panels and data types) and mentioning any restrictions on availability.

• If a dataset has a Digital Object Identifier (DOI) as its unique identifier, we strongly encourage including this in the Reference list and citing the dataset in the Methods.

We recommend that you upload the step-by-step protocols used in this manuscript to protocols.io. More details can found at https://www.protocols.io/help/publish-articles.

DISPLAY ITEMS – main display items are limited to 6-8 main figures and/or main tables. For Supplementary Information see below.

FIGURES – Colour figure publication costs $395 per colour figure. All panels of a multi-panel figure must be logically connected and arranged as they would appear in the final version. Unnecessary figures and figure panels should be avoided (e.g. data presented in small tables could be stated briefly in the text instead).

All imaging data should be accompanied by scale bars, which should be defined in the legend.
Cropped images of gels/blots are acceptable, but need to be accompanied by size markers, and to retain visible background signal within the linear range (i.e. should not be saturated). The boundaries of panels with low background have to be demarked with black lines. Splicing of panels should only be considered if unavoidable, and must be clearly marked on the figure, and noted in the legend with a statement on whether the samples were obtained and processed simultaneously. Quantitative comparisons between samples on different gels/blots are discouraged; if this is unavoidable, it has be performed for samples derived from the same experiment with gels/blots were processed in parallel, which needs to be stated in the legend.

Figures should be provided at approximately the size that they are to be printed at (single column is 86 mm, double column is 170 mm) and should not exceed an A4 page (8.5 x 11"). Reduction to the scale that will be used on the page is not necessary, but multi-panel figures should be sized so that the whole figure can be reduced by the same amount at the smallest size at which essential details in each panel are visible. In the interest of our colour-blind readers we ask that you avoid using red and green for contrast in figures. Replacing red with magenta and green with turquoise are two possible colour-safe alternatives. Lines with widths of less than 1 point should be avoided. Sans serif typefaces, such as Helvetica (preferred) or Arial should be used. All text that forms part of a figure should be rewritable and removable.

We accept files from the following graphics packages in either PC or Macintosh format:

- For line art, graphs, charts and schematics we prefer Adobe Illustrator (.AI), Encapsulated PostScript (.EPS) or Portable Document Format (.PDF). Files should be saved or exported as such directly from the application in which they were made, to allow us to restyle them according to our journal house style.

- We accept PowerPoint (.PPT) files if they are fully editable. However, please refrain from adding PowerPoint graphical effects to objects, as this results in them outputting poor quality raster art. Text used for PowerPoint figures should be Helvetica (preferred) or Arial.

- We do not recommend using Adobe Photoshop for designing figures, but we can accept Photoshop generated (.PSD or .TIFF) files only if each element included in the figure (text, labels, pictures, graphs, arrows and scale bars) are on separate layers. All text should be editable in 'type layers' and line-art such as graphs and other simple schematics should be preserved and embedded within 'vector smart objects' - not flattened raster/bitmap graphics.

- Some programs can generate Postscript by 'printing to file' (found in the Print dialogue). If using an application not listed above, save the file in PostScript format or email our Art Editor, Allen Beattie for advice (a.beattie@nature.com).

Regardless of format, all figures must be vector graphic compatible files, not supplied in a flattened raster/bitmap graphics format, but should be fully editable, allowing us to highlight/copy/paste all text and move individual parts of the figures (i.e. arrows, lines, x and y axes, graphs, tick marks, scale bars etc). The only parts of the figure that should be in pixel raster/bitmap format are photographic images or 3D rendered graphics/complex technical illustrations.

All placed images (i.e. a photo incorporated into a figure) should be on a separate layer and independent from any superimposed scale bars or text. Individual photographic images must be a minimum of 300+ DPI (at actual size) or kept constant from the original picture acquisition and not decreased in resolution post image acquisition. All colour artwork should be RGB format.

FIGURE LEGENDS – must not exceed 350 words for each figure to allow fit on a single printed NCB page together with the figure. They must include a brief title for the whole figure, and short descriptions of each panel with definitions of the symbols used, but without detailing methodology.

TABLES – main tables should be provided as individual Word files, together with a brief title and legend. For supplementary tables see below.

SUPPLEMENTARY INFORMATION – Supplementary information is material directly relevant to the conclusion of a paper, but which cannot be included in the printed version in order to keep the manuscript concise and accessible to the general reader. Supplementary information is an integral part of a Nature Cell Biology publication and should be prepared and presented with as much care as the main display item, but it must not include non-essential data or text, which may be removed at the editor's discretion. All supplementary material is fully peer-reviewed and published online as part of the HTML version of the manuscript. Supplementary Figures and Supplementary Notes are appended at the end of the main PDF of the published manuscript.

Supplementary items should relate to a main text figure, wherever possible, and should be mentioned sequentially in the main manuscript, designated as Supplementary Figure, Table, Video, or Note, and numbered continuously (e.g. Supplementary Figure 1, Supplementary Figure 2, Supplementary Table 1, Supplementary Table 2 etc.).

Unprocessed scans of all key data generated through electrophoretic separation techniques need to be presented in a supplementary figure that should be labeled and numbered as the final supplementary figure, and should be mentioned in every relevant figure legend. This figure does not count towards the total number of figures and is the only figure that can be displayed over multiple pages, but should be provided as a single file, in PDF or TIFF format. Data in this figure can be displayed in a relatively informal style, but size markers and the figures panels corresponding to the presented data must be indicated.

The total number of Supplementary Figures (not including the "unprocessed scans" Supplementary Figure) should not exceed the number of main display items (figures and/or tables (see our Guide to Authors and March 2012 editorial http://www.nature.com/ncb/authors/submit/index.html#suppinfo; http://www.nature.com/ncb/journal/v14/n3/index.html#ed). No restrictions apply to Supplementary Tables or Videos, but we advise authors to be selective in including supplemental data.

Each Supplementary Figure should be provided as a single page and as an individual file in one of our accepted figure formats and should be presented according to our figure guidelines (see above). Supplementary Tables should be provided as individual Excel files. Supplementary Videos should be provided as .avi or .mov files up to 50 MB in size. Supplementary Figures, Tables and Videos much be accompanied by a separate Word document including titles and legends.

GUIDELINES FOR EXPERIMENTAL AND STATISTICAL REPORTING

REPORTING REQUIREMENTS – To improve the quality of methods and statistics reporting in our papers we have recently revised the reporting checklist we introduced in 2013. We are now asking all life sciences authors to complete two items: an Editorial Policy Checklist (found here https://www.nature.com/authors/policies/Policy.pdf) that verifies compliance with all required editorial policies and a Reporting Summary (found here https://www.nature.com/authors/policies/ReportingSummary.pdf) that collects information on experimental design and reagents. These documents are available to referees to aid the evaluation of the manuscript. Please note that these forms are dynamic 'smart pdfs' and must therefore be downloaded and completed in Adobe Reader. We will then flatten them for ease of use by the reviewers. If you would like to reference the guidance text as you complete the template, please access these flattened versions at http://www.nature.com/authors/policies/availability.html.

STATISTICS – Wherever statistics have been derived the legend needs to provide the n number (i.e. the sample size used to derive statistics) as a precise value (not a range), and define what this value represents. Error bars need to be defined in the legends (e.g. SD, SEM) together with a measure of centre (e.g. mean, median). Box plots need to be defined in terms of minima, maxima, centre, and percentiles. Ranges are more appropriate than standard errors for small data sets. Wherever statistical significance has been derived, precise p values need to be provided and the statistical test used needs to be stated in the legend. Statistics such as error bars must not be derived from n<3. For sample sizes of n<5 please plot the individual data points rather than providing bar graphs. Deriving statistics from technical replicate samples, rather than biological replicates is strongly discouraged. Wherever statistical significance has been derived, precise p values need to be provided and the statistical test stated in the legend.

Information on how many times each experiment was repeated independently with similar results needs to be provided in the legends and/or Methods for all experiments, and in particular wherever representative experiments are shown.

We strongly recommend the presentation of source data for graphical and statistical analyses as a separate Supplementary Table, and request that source data for all independent repeats are provided when representative experiments of multiple independent repeats, or averages of two independent experiments are presented. This supplementary table should be in Excel format, with data for different figures provided as different sheets within a single Excel file. It should be labelled and numbered as one of the supplementary tables, titled "Statistics Source Data", and mentioned in all relevant figure legends.

--------- Please don't hesitate to contact NCB@nature.com should you have queries about any of the above requirements ---------

Version 1:

Decision Letter:

*Please delete the link to your author homepage if you wish to forward this email to co-authors.

Dear Dr Petkevicius,

Thank you again for submitting your manuscript, "Stereospecific GPG acylation by CLN8 drives BMP biosynthesis and its loss leads to Batten disease", to Nature Cell Biology. It has now been seen by 4 referees, who are experts in lipid metabolism (Referee #1); LSDs (Referee #2); lipidomics, lipid biology (Referee #3); and cryo-EM (Referee #4). As you will see from their comments (attached below), they found the work of potential interest but have raised substantial concerns, which in our view would need to be addressed with considerable revisions before we can consider publication in Nature Cell Biology.

Nature Cell Biology editors discuss the referee reports in detail within the editorial team, including the chief editor, to identify key referee points that should be addressed with priority, and requests that are overruled as being beyond the scope of the current study. To guide the scope of the revisions, I have listed these points below. Our standard revision period is six months, and we are committed to providing a fair and constructive peer-review process, so please feel free to contact me if you would like to discuss any of the referee comments further or anticipate any issues addressing the reviews.

I should stress that the referees' concerns point to a premature dataset and their concerns would need to be addressed thoroughly experimentally, and reconsideration of the study for this journal and re-engagement of referees will depend on the strength of the revisions. In particular, it would be essential to address the reviews as follows:

-- Rev#4's comments about the model quality, structural analysis, and mechanism of action need to be addressed thoroughly and in full;

-- additional phenotypic characterizations and expanded supplementation studies as suggested by Rev#1 in point #3 and Rev#2 in points #3-4 should also be a priority in revision;

-- Rev#3's comments about the tracer data (#1, #5) and Rev#1's comments about the mass spec analyses should be addressed (#1-2).

-- Please address with discussion the open questions raised by the experts on the panel, including trafficking of lipids from ER to lysosomes & BMP synthesis pathways per Rev#1-Rev#2-Rev#3, and discussion of the relevance of the data to brain metabolic health/disease (Rev#1, Rev#3)

-- All other referee concerns pertaining to strengthening existing data, providing controls, methodological details, clarifications and textual changes, should also be addressed.

-- Finally, please pay close attention to our guidelines on statistical and methodological reporting (listed below) as failure to do so may delay the reconsideration of the revised manuscript. In particular, please provide:

- a Supplementary Figure including unprocessed images of all gels/blots in the form of a multi-page pdf file. Please ensure that blots/gels are labeled and the sections presented in the figures are clearly indicated.

- a Supplementary Table including all numerical source data in Excel format, with data for different figures provided as different sheets within a single Excel file. The file should include source data giving rise to graphical representations and statistical descriptions in the paper and for all instances where the figures present representative experiments of multiple independent repeats, the source data of all repeats should be provided.

We would be happy to consider a revised manuscript that would satisfactorily address these points, unless a similar paper is published elsewhere, or is accepted for publication in Nature Cell Biology in the meantime.

When revising the manuscript please:

- ensure that it conforms to our format instructions and publication policies (see below and https://www.nature.com/nature/for-authors).

- provide a point-by-point rebuttal to the full referee reports verbatim, as provided at the end of this letter.

- for any revision that includes light microscopy data, we ask our authors to please include a completed light microscopy reporting table https://www.nature.com/documents/Light_microscopy_reporting_table.xlsx to ensure the methods are described thoroughly. The table will be available to reviewers and ultimately published should the manuscript be accepted at the journal.

- provide the completed Reporting Summary (found here https://www.nature.com/documents/nr-reporting-summary.pdf). This is essential for reconsideration of the manuscript will be available to editors and referees in the event of peer review. For more information see http://www.nature.com/authors/policies/availability.html or contact me.

When submitting the revised version of your manuscript, please pay close attention to our href="https://www.nature.com/nature-portfolio/editorial-policies/image-integrity">Digital Image Integrity Guidelines. and to the following points below:

-- that unprocessed scans are clearly labelled and match the gels and western blots presented in figures.
-- that control panels for gels and western blots are appropriately described as loading on sample processing controls
-- all images in the paper are checked for duplication of panels and for splicing of gel lanes.

Finally, please ensure that you retain unprocessed data and metadata files after publication, ideally archiving data in perpetuity, as these may be requested during the peer review and production process or after publication if any issues arise.

Nature Cell Biology is committed to improving transparency in authorship. As part of our efforts in this direction, we are now requesting that all authors identified as 'corresponding author' on published papers create and link their Open Researcher and Contributor Identifier (ORCID) with their account on the Manuscript Tracking System (MTS), prior to acceptance. ORCID helps the scientific community achieve unambiguous attribution of all scholarly contributions. You can create and link your ORCID from the home page of the MTS by clicking on 'Modify my Springer Nature account'. For more information please visit please visit www.springernature.com/orcid.

This journal strongly supports public availability of data. Please place the data used in your paper into a public data repository, or alternatively, present the data as Supplementary Information. If data can only be shared on request, please explain why in your Data Availability Statement, and also in the correspondence with your editor. Please note that for some data types, deposition in a public repository is mandatory - more information on our data deposition policies and available repositories appears below.

Please submit the revised manuscript files and the point-by-point rebuttal to the referee comments using this link:

Link Redacted

*This url links to your confidential home page and associated information about manuscripts you may have submitted or be reviewing for us. If you wish to forward this email to co-authors, please delete the link to your homepage.

We hope that you will find our referees' comments and editorial guidance helpful. Please do not hesitate to contact me if there is anything you would like to discuss. Thank you again for considering NCB for your work.

Best wishes,

Melina

Melina Casadio, PhD
Senior Editor, Nature Cell Biology
ORCID ID: https://orcid.org/0000-0003-2389-2243

Reviewers' Comments:

Reviewer #1 (Remarks to the Author):

In this manuscript, Pradeep K. Sheokand et al. present compelling data identifying CLN8 as a stereospecific acyltransferase that converts S,S-glycerophosphoglycerol (PGP) to S,S-lysophosphatidylglycerol (LPG), a substrate precursor for bis(monoacylglycero)phosphate (BMP) biosynthesis. Using high-resolution cryo-electron microscopy (cryo-EM), the authors reveal a ping-pong acyl transfer mechanism for CLN8. Furthermore, they demonstrate that mutations associated with CLN8-related Batten disease resulted in a graded loss of enzymatic activity, correlating directly with reduced BMP levels and clinical severity. These findings offer robust evidence that disrupted BMP biosynthesis is a primary driver of Batten disease. Importantly, the study shows that exogenous S,S-LPG supplementation can restore BMP level both in vitro and in vivo, leading to modest improvements in neurological phenotypes in a cln8 mutant zebrafish model. Given the underexplored role of BMP in lysosomal regulation, identifying CLN8's specific function is a significant advancement for the field.

While the mass spectrometry (MS)-based lipidomics is generally robust, refinements regarding isomer resolution and quantification transparency are necessary to strengthen the study's rigor. I strongly support the publication of this work pending these revisions.

1. The study’s key rescue experiments rely on distinguishing S,S-LPG from R,rac-LPG, and resolving BMP positional isomers (2,2’-, 2,3’-, 3,3’- acyl configurations). While LC-MS/MS is used for detection, the manuscript lacks details on whether these stereoisomers/positional isomers achieve baseline separation under the described chromatographic conditions. For example, Supplementary Table 5 lists MRM transitions but does not provide retention time windows or chromatograms demonstrating resolved peaks for S,S-LPG vs. other isomers. Because ion ionization efficiency can vary between stereoisomers, the authors should provide calibration curves using purified synthetic standards for each isomer to correct for potential quantification bias. This is particularly critical for the in vivo rescue experiments (Fig. 4d) and BMP isomer analysis in Cln8R24G mice (Fig. 3e), where conclusions about residual enzymatic activity depend on relative isomer abundance.

2. The manuscript identifies 2-acyl-LPG as the CLN8 product and 2,2’-BMP as the mature end product. However, the MS/MS-based assignment of BMP positional isomers (e.g., Extended Data Fig. 2h) requires more explicit validation. The assignment of 2,2’ vs. 2,3’ isomers currently relies on retention time comparison. The authors should provide MS/MS spectra of synthetic BMP positional isomers to highlight diagnostic fragment ions that distinguish these configurations. Given that BMP isomers differ in biological functon(such as resistance to lysosomal hydrolysis), unambiguous assignment is essential for the study's mechanistic claims.

3. The rescue experiments with S,S-LPG are a highlight of the paper, yet there is a slight imbalance in the data provided for each model. In mouse models, the authors provide BMP restoration data but lack accompanying phenotypic/behavioral rescue data. In zebrafish models, the authors show modest neurological improvement but lack biochemical data confirming BMP restoration levels. For both models, the authors should indicate the extent to which BMP and locomotor activity are restored relative to wild type baseline. This is necessary to evaluate the true therapeutic potential of S,S-LPG supplementation.

4. The manuscript proposes stereochemically defined S,S-LPG as a therapeutic strategy. Since impaired BMP synthesis is the core defect, the discussion would benefit from a brief comparison of the chemical and cellular advantages of supplementing with the LPG precursor versus the BMP end-product (e.g., bioavailability, stability, or cellular uptake).

5. The manuscript would benefit from an expanded discussion section that better contextualizes the CLN8 pathway within the broader framework of cellular physiology. Specifically, I recommend further elaborating on the cellular origins of GPG, the mechanisms underlying LPG membrane transport, and related processes.

6. For the sake of non-specialist readers, please add positional labels to the acyl chains in the chemical structure figures where appropriate.

Reviewer #1 (Remarks on Protocol(s)):

Please refer to my related comments in the general comments section.

Reviewer #2 (Remarks to the Author):

Sheokand et al. present a large body of data relating to the enzymatic activity of CLN8 and its involvement in Batten disease. They have identified its substrate, and used cryoelectron microscopy to derive its three-dimensional structure. With this structure in hand, the authors have examined a number of mutations in the CLN8 gene associated with forms of Batten disease differing in severity, and have been able to establish that the different mutations affect the catalytic mechanism of CLN8 in different ways. Further, the authors have examined two Cln8-deficient mouse models to establish the consequences of Cln8 deficiency on the BMP metabolic pathway. In the final section of the manuscript, the authors show that S,S-LPG supplementation restores BMP synthesis in CNL8-deficient HeLa cells and mice, and that this treatment improves the locomotion of cln8-mutant zebrafish. These results warrant further studies to establish whether S,S-LPG supplementation provides clinical benefits to Batten disease patients carrying CLN8 mutations.

Comments

[1] The title of the manuscript contains three abbreviations that are not too common. The more abbreviations in the title of a research paper, the fewer readers it attracts, and the fewer times it gets cited. In particular for a journal with a such wide readership as Nature Cell Biology, the authors should strive to make the title of their research paper intelligible and appealing for the wider research community.

[2] The writing of the Main section can be improved. Again, the wide readership of Nature Cell Biology includes many who are not specialists in BMP metabolism (second paragraph), and the link with Batten disease (first paragraph) may not be obvious for them.

[3] On page 8, penultimate paragraph, results are presented from administering S,S-LPG or R,rac-LPG to homozygous Cln8mnd mice (Fig. 4 c and d). Exogenous S,S-LPG resulted in the appearance of BMP in the mutant mice. However, for therapeutic purposes, it is critical to establish to what extent a treatment restores a deficit relative to its physiological value. So, it would be good to extend Fig. 4 d with the BMP level of untreated WT mice of the same strain background as the homozygous Cln8mnd mice.

[4A] On page 8, last paragraph, results are presented from administering S,S-LPG or R,rac-LPG to cln8 mutant zebrafish larvae. S,S-LPG lessened the pathology of the mutant larvae (Figures 4 f, g, and h). Nevertheless, also for this experiment, the efficacy of the therapeutic intervention can only be properly assessed when the outcomes are compared to physiological standards. Therefore, it would be good to extend Figures 4 f, g, and h with the corresponding data of untreated wild-type larvae of the same strain as the mutants.
[4B] Have the authors determined whether S,S-LPG supplementation restores the levels of BMP in cln8 mutant zebrafish larvae?

[5] The bottom panels of Extended Data Fig. 4 are very small, and their lettering is challenging to read. It would be good if these panels were replaced with larger versions.
[6] Discussion: In a previous study (DOI: 10.1126/sciadv.adr3723), the authors used the AlphaFold2 model of CLN8. In would be good to compare and contrast the AlphaFold2 model with the cryoelectron microscopy-derived structure of CLN8 presented here. Does the

empirical model differ much from the in silico model?

[7] Recently, Singh et al. (https://doi.org/10.1016/j.cell.2024.09.036) proposed a metabolic pathway for BMP synthesis that involves lysosomal phospholipases D3/D4 (PLD3/4). The authors of the current manuscript are encouraged to expand their Discussion, comparing and contrasting it with their own work. It is important that the authors point out in some detail how the approaches of Singh et al. led to insights that are different from those of the current manuscript. Alternatively, the authors could provide a synthesis of both studies, showing how the roles of PLD3/4 and CLN8 may be complementary, following the wider scope of the Discussion of Bulfon et al. (https://doi.org/10.1038/s41467-024-54213-1). With the current knowledge, would it be is possible to arrive at a unified vision of BMP synthesis, or are there outstanding issues to be resolved experimentally?

Reviewer #2 (Remarks on Protocol(s)):

The methods have been adequately described.

Reviewer #3 (Remarks to the Author):

The authors provide a comprehensive biochemical, structural, and in vivo characterization of CLN8, definitively establishing it as the enzyme responsible for stereospecific glycerophosphoglycerol (GPG) acylation during bis(monoacylglycero)phosphate (BMP) biosynthesis. By integrating enzymology, stereochemically defined lipid substrates, high-resolution cryo-EM structures, disease-associated mutations, and multiple animal models, the study convincingly links impaired BMP synthesis to the pathogenesis of CLN8-associated Batten disease. Overall, this work represents a technical and conceptual advance in the fields of lysosomal lipid metabolism and neurodegenerative disease.

Comments/Suggestions
1. While the enzymatic and tracer data convincingly establish GPG as a bona fide substrate for CLN8, additional discussion of the physiological sources, abundance, and turnover of S,S-GPG would help contextualize how this pathway operates under basal versus stress or disease conditions.
2. The proposed model requires trafficking of CLN8-generated S,S-LPG from the ER to the lysosome for CLN5-mediated condensation. This step remains mechanistically unresolved. A brief discussion of potential trafficking routes would strengthen the conceptual model.
3. The S,S-LPG rescue experiments are well executed. It may be helpful to more explicitly distinguish functional rescue from complete restoration of endogenous lipid diversity and remodeling dynamics.
4. Given the neurological focus of Batten disease, a short discussion acknowledging the limitations of tracer delivery across the blood–brain barrier, and how brain BMP pools are maintained, would further align the metabolic data with disease relevance.
5. In several experiments, exogenous lipid substrates or tracers are applied at concentrations and for durations that may exceed endogenous levels or turnover rates. While appropriate for establishing enzymatic specificity and pathway connectivity, a brief discussion distinguishing demonstration of pathway capability from physiological contribution would help prevent overinterpretation.
6. Most mechanistic experiments are performed in HeLa and HEK293-derived systems, which may not fully capture the lipid metabolic context relevant to neurodegeneration. Given the otherwise high level of rigor and depth in the study, the limited use of more disease-relevant cellular models stands out and warrants discussion.

Reviewer #4 (Remarks to the Author):

See attatched

Reviewer #4 (Remarks on Protocol(s)):

Protein purification and cryo-EM collection/analysis are sound.

GUIDELINES FOR MANUSCRIPT SUBMISSION TO NATURE CELL BIOLOGY

READABILITY OF MANUSCRIPTS – Nature Cell Biology is read by cell biologists from diverse backgrounds, many of whom are not native English speakers. Authors should aim to communicate their findings clearly, explaining technical jargon that might be unfamiliar to non-specialists, and avoiding non-standard abbreviations. Titles and abstracts should concisely communicate the main findings of the study, and the background, rationale, results and conclusions should be clearly explained in the manuscript in a manner accessible to a broad cell biology audience. Nature Cell Biology uses British spelling.

MANUSCRIPT FORMAT – please follow the guidelines listed in our Guide to Authors regarding manuscript formats at Nature Cell Biology.

TITLE – should be no more than 100 characters including spaces, without punctuation and avoiding technical terms, abbreviations, and active verbs..

AUTHOR NAMES – should be given in full.

AUTHOR AFFILIATIONS – should be denoted with numerical superscripts (not symbols) preceding the names. Full addresses should be included, with US states in full and providing zip/post codes. The corresponding author is denoted by: "Correspondence should be addressed to [initials]."

ABSTRACT AND MAIN TEXT – please follow the guidelines that are specific to the format of your manuscript, as listed in our Guide to Authors (http://www.nature.com/ncb/pdf/ncb_gta.pdf) Briefly, Nature Cell Biology Articles, Resources and Technical Reports have 3500 words, including a 150 word abstract, and the main text is subdivided in Introduction, Results, and Discussion sections. Nature Cell Biology Letters have up to 2500 words, including a 180 word introductory paragraph (abstract), and the text is not subdivided in sections.

ACKNOWLEDGEMENTS – should be kept brief. Professional titles and affiliations are unnecessary. Grant numbers can be listed.

AUTHOR CONTRIBUTIONS – must be included after the Acknowledgements, detailing the contributions of each author to the paper (e.g. experimental work, project planning, data analysis etc.). Each author should be listed by his/her initials.

FINANCIAL AND NON-FINANCIAL COMPETING INTERESTS – the authors must include one of three declarations: (1) that they have no financial and non-financial competing interests; (2) that they have financial and non-financial competing interests; or (3) that they decline to respond, after the Author Contributions section. This statement will be published with the article, and in cases where financial and non-financial competing interests are declared, these will be itemized in a web supplement to the article. For further details please see https://www.nature.com/licenceforms/nrg/competing-interests.pdf.

REFERENCES – are limited to a total of 70 for Articles, Resources, Technical Reports; and 40 for Letters. This includes references in the main text and Methods combined. References must be numbered sequentially as they appear in the main text, tables and figure legends and Methods and must follow the precise style of Nature Cell Biology references. References only cited in the Methods should be numbered consecutively following the last reference cited in the main text. References only associated with Supplementary Information (e.g. in supplementary legends) do not count toward the total reference limit and do not need to be cited in numerical continuity with references in the main text. Only published papers can be cited, and each publication cited should be included in the numbered reference list, which should include the manuscript titles. Footnotes are not permitted.

METHODS – Nature Cell Biology publishes methods online. The methods section should be provided as a separate Word document, which will be copyedited and appended to the manuscript PDF, and incorporated within the HTML format of the paper.

Methods should be written concisely, but should contain all elements necessary to allow interpretation and replication of the results. As a guideline, Methods sections typically do not exceed 3,000 words. The Methods should be divided into subsections listing reagents and techniques. When citing previous methods, accurate references should be provided and any alterations should be noted. Information must be provided about: antibody dilutions, company names, catalogue numbers and clone numbers for monoclonal antibodies; sequences of RNAi and cDNA probes/primers or company names and catalogue numbers if reagents are commercial; cell line names, sources and information on cell line identity and authentication. Animal studies and experiments involving human subjects must be reported in detail, identifying the committees approving the protocols. For studies involving human subjects/samples, a statement must be included confirming that informed consent was obtained. Statistical analyses and information on the reproducibility of experimental results should be provided in a section titled “Statistics and Reproducibility”.

All Nature Cell Biology manuscripts submitted on or after March 21 2016 must include a Data availability statement as a separate section after Methods but before references, under the heading "Data Availability". . For Springer Nature policies on data availability see http://www.nature.com/authors/policies/availability.html; for more information on this particular policy see http://www.nature.com/authors/policies/data/data-availability-statements-data-citations.pdf. The Data availability statement should include:

• Accession codes for primary datasets (generated during the study under consideration and designated as "primary accessions") and secondary datasets (published datasets reanalysed during the study under consideration, designated as "referenced accessions"). For primary accessions data should be made public to coincide with publication of the manuscript. A list of data types for which submission to community-endorsed public repositories is mandated (including sequence, structure, microarray, deep sequencing data) can be found here http://www.nature.com/authors/policies/availability.html#data.

• Unique identifiers (accession codes, DOIs or other unique persistent identifier) and hyperlinks for datasets deposited in an approved repository, but for which data deposition is not mandated (see here for details http://www.nature.com/sdata/data-policies/repositories).

• At a minimum, please include a statement confirming that all relevant data are available from the authors, and/or are included with the manuscript (e.g. as source data or supplementary information), listing which data are included (e.g. by figure panels and data types) and mentioning any restrictions on availability.

• If a dataset has a Digital Object Identifier (DOI) as its unique identifier, we strongly encourage including this in the Reference list and citing the dataset in the Methods.

We recommend that you upload the step-by-step protocols used in this manuscript to protocols.io. More details can found at https://www.protocols.io/help/publish-articles.

DISPLAY ITEMS – main display items are limited to 6-8 main figures and/or main tables for Articles, Resources, Technical Reports; and 5 main figures and/or main tables for Letters. For Supplementary Information see below.

FIGURES – Colour figure publication costs $600 for the first, and $300 for each subsequent colour figure. All panels of a multi-panel figure must be logically connected and arranged as they would appear in the final version. Unnecessary figures and figure panels should be avoided (e.g. data presented in small tables could be stated briefly in the text instead).

All imaging data should be accompanied by scale bars, which should be defined in the legend.

Cropped images of gels/blots are acceptable, but need to be accompanied by size markers, and to retain visible background signal within the linear range (i.e. should not be saturated). The boundaries of panels with low background have to be demarked with black lines. Splicing of panels should only be considered if unavoidable, and must be clearly marked on the figure, and noted in the legend with a statement on whether the samples were obtained and processed simultaneously. Quantitative comparisons between samples on different gels/blots are discouraged; if this is unavoidable, it should only be performed for samples derived from the same experiment with gels/blots were processed in parallel, which needs to be stated in the legend.

Figures should be provided at approximately the size that they are to be printed at (single column is 86 mm, double column is 170 mm) and should not exceed an A4 page (8.5 x 11"). Reduction to the scale that will be used on the page is not necessary, but multi-panel figures should be sized so that the whole figure can be reduced by the same amount at the smallest size at which essential details in each panel are visible. In the interest of our colour-blind readers we ask that you avoid using red and green for contrast in figures. Replacing red with magenta and green with turquoise are two possible colour-safe alternatives. Lines with widths of less than 1 point should be avoided. Sans serif typefaces, such as Helvetica (preferred) or Arial should be used. All text that forms part of a figure should be rewritable and removable.

We accept files from the following graphics packages in either PC or Macintosh format:

- For line art, graphs, charts and schematics we prefer Adobe Illustrator (.AI), Encapsulated PostScript (.EPS) or Portable Document Format (.PDF). Files should be saved or exported as such directly from the application in which they were made, to allow us to restyle them according to our journal house style.

- We accept PowerPoint (.PPT) files if they are fully editable. However, please refrain from adding PowerPoint graphical effects to objects, as this results in them outputting poor quality raster art. Text used for PowerPoint figures should be Helvetica (preferred) or Arial.

- We do not recommend using Adobe Photoshop for designing figures, but we can accept Photoshop generated (.PSD or .TIFF) files only if each element included in the figure (text, labels, pictures, graphs, arrows and scale bars) are on separate layers. All text should be editable in 'type layers' and line-art such as graphs and other simple schematics should be preserved and embedded within 'vector smart objects' - not flattened raster/bitmap graphics.

- Some programs can generate Postscript by 'printing to file' (found in the Print dialogue). If using an application not listed above, save the file in PostScript format or email our Art Editor, Allen Beattie for advice (a.beattie@nature.com).

Regardless of format, all figures must be vector graphic compatible files, not supplied in a flattened raster/bitmap graphics format, but should be fully editable, allowing us to highlight/copy/paste all text and move individual parts of the figures (i.e. arrows, lines, x and y axes, graphs, tick marks, scale bars etc.). The only parts of the figure that should be in pixel raster/bitmap format are photographic images or 3D rendered graphics/complex technical illustrations.

All placed images (i.e. a photo incorporated into a figure) should be on a separate layer and independent from any superimposed scale bars or text. Individual photographic images must be a minimum of 300+ DPI (at actual size) or kept constant from the original picture acquisition and not decreased in resolution post image acquisition. All colour artwork should be RGB format.

EXTENDED DATA FIGURES - When re-submitting your manuscript, please ensure that any supplementary figures and tables that are crucial to the manuscript's conclusions are converted into Extended Data figures and tables to increase visibility of these data. Extended Data figures and tables are online-only (present in the online PDF and full-text HTML versions of the paper), peer-reviewed display items that provide essential background to the article but are not included in the main article due to space constraints. A maximum of ten Extended Data display items (figures and tables) is permitted.

FIGURE LEGENDS – must not exceed 350 words for each figure to allow fit on a single printed NCB page together with the figure. They must include a brief title for the whole figure, and short descriptions of each panel with definitions of the symbols used, but without detailing methodology.

TABLES – main tables should be provided as individual Word files, together with a brief title and legend. For supplementary tables see below.

SUPPLEMENTARY INFORMATION – Supplementary information is material directly relevant to the conclusion of a paper, but which cannot be included in the printed version in order to keep the manuscript concise and accessible to the general reader. Supplementary information is an integral part of a Nature Cell Biology publication and should be prepared and presented with as much care as the main display item, but it must not include non-essential data or text, which may be removed at the editor's discretion. All supplementary material is fully peer-reviewed and published online as part of the HTML version of the manuscript. Supplementary Figures and Supplementary Notes are appended at the end of the main PDF of the published manuscript.

Supplementary items should relate to a main text figure, wherever possible, and should be mentioned sequentially in the main manuscript, designated as Supplementary Figure, Table, Video, or Note, and numbered continuously (e.g. Supplementary Figure 1, Supplementary Figure 2, Supplementary Table 1, Supplementary Table 2 etc.).

Unprocessed scans of all key data generated through electrophoretic separation techniques need to be presented in a supplementary figure that should be labelled and numbered as the final supplementary figure, and should be mentioned in every relevant figure legend. This figure does not count towards the total number of figures and is the only figure that can be displayed over multiple pages, but should be provided as a single file, in PDF or TIFF format. Data in this figure can be displayed in a relatively informal style, but size markers and the figures panels corresponding to the presented data must be indicated.

The total number of Supplementary Figures (not including the "unprocessed scans" Supplementary Figure) should not exceed the number of main display items (figures and/or tables (see our Guide to Authors and March 2012 editorial http://www.nature.com/ncb/authors/submit/index.html#suppinfo; http://www.nature.com/ncb/journal/v14/n3/index.html#ed). No restrictions apply to Supplementary Tables or Videos, but we advise authors to be selective in including supplemental data.

Each Supplementary Figure should be provided as a single page and as an individual file in one of our accepted figure formats and should be presented according to our figure guidelines (see above). Supplementary Tables should be provided as individual Excel files. Supplementary Videos should be provided as .avi or .mov files up to 50 MB in size. Supplementary Figures, Tables and Videos much be accompanied by a separate Word document including titles and legends.

GUIDELINES FOR EXPERIMENTAL AND STATISTICAL REPORTING

REPORTING REQUIREMENTS – We are trying to improve the quality of methods and statistics reporting in our papers. To that end, we are now asking authors to complete a reporting summary that collects information on experimental design and reagents. The Reporting Summary can be found here https://www.nature.com/documents/nr-reporting-summary.pdf)If you would like to reference the guidance text as you complete the template, please access these flattened versions at http://www.nature.com/authors/policies/availability.html.

STATISTICS – Wherever statistics have been derived the legend needs to provide the n number (i.e. the sample size used to derive statistics) as a precise value (not a range), and define what this value represents. Error bars need to be defined in the legends (e.g. SD, SEM) together with a measure of centre (e.g. mean, median). Box plots need to be defined in terms of minima, maxima, centre, and percentiles. Ranges are more appropriate than standard errors for small data sets. Wherever statistical significance has been derived, precise p values need to be provided and the statistical test used needs to be stated in the legend. Statistics such as error bars must not be derived from n<3. For sample sizes of n<5 please plot the individual data points rather than providing bar graphs. Deriving statistics from technical replicate samples, rather than biological replicates is strongly discouraged. Wherever statistical significance has been derived, precise p values need to be provided and the statistical test stated in the legend.

Information on how many times each experiment was repeated independently with similar results needs to be provided in the legends and/or Methods for all experiments, and in particular wherever representative experiments are shown.

We strongly recommend the presentation of source data for graphical and statistical analyses as a separate Supplementary Table, and request that source data for all independent repeats are provided when representative experiments of multiple independent repeats, or averages of two independent experiments are presented. This supplementary table should be in Excel format, with data for different figures provided as different sheets within a single Excel file. It should be labelled and numbered as one of the supplementary tables, titled "Statistics Source Data", and mentioned in all relevant figure legends.

--------- Please don't hesitate to contact NCB@nature.com should you have queries about any of the above requirements ---------

Version 2:

Decision Letter:

*Please delete the link to your author homepage if you wish to forward this email to co-authors.

Dear Dr Petkevicius,

Thank you for your patience with the re-review process.

Your manuscript, "The Batten disease protein CLN8 is a stereospecific acyltransferase in bis(monoacylglycero)phosphate biosynthesis", has now been seen by the original referees. As you will see from their comments (attached below), they continue to find this work of interest but have raised some important persisting concerns. Although we are also very interested in this study, we believe that their concerns should be addressed before we can consider publication in Nature Cell Biology.

We have discussed their remaining concerns - you will see that Rev#2 asked for expanded discussion, which we agree should be added. Rev#4 did not find your responses convincing regarding the quality of the model built into TM4. Please follow this referee's recommendations for additional cryo-EM data processing efforts - this is important to ensure the model is convincing to readers and experts in this area.

We would like to receive the revision within four weeks. Please let us know if this is not possible or if you anticipate delays. Thank you in advance for your efforts to resolve these outstanding remaining issues.

To save time with the resubmission and next steps editorially, we also encourage you to check that your Article meets our formatting requirements, which include: having the manuscript divided into Introduction/Results (with subsections)/Discussion (no subsections); up to 8 main and 10 Extended Data figures;. Please provide ALL supplementary tables in excel, ideally in one workbook with one numbered supplementary table per tab. NCB Articles DO NOT have "main" tables or "ED" tables. NCB Articles only have "supplementary" tables, in excel (unlike some of our sister journals).

REPORTING OF STRUCTURAL DATA: If there are additional or modified structures in the final revision, please submit the corresponding PDB validation reports with your other files, and use the figshare integration system to provide access to maps, half-maps, and models

IMPORTANT: Manuscripts reporting new structures should contain a SUPPLEMENTARY (not main) table summarizing structural and refinement statistics. Please include these tables for new cryo-EM or -ET (https://www.nature.com/documents/nr-tables-cryo-em.doc[KC3.1] and modifying this file for ET), NMR (https://www.nature.com/documents/nr-tables-nmr.doc) and X-ray crystallography (https://www.nature.com/documents/nr-tables-xray.doc). To facilitate assessment of the quality of the structural data, a stereo image of a portion of the electron density map (for crystallography papers) or of the superimposed lowest energy structures (>10; for NMR papers)

should be provided with the submitted manuscript. If the reported structure represents a novel overall fold, a stereo image of the entire structure (as a backbone trace) should also be provided. For cryo-EM structures, a representative micrograph showing individual particles should be provided in the figures alongside a processing workflow (please see our sister journal Editorial for more information: https://www.nature.com/articles/s41594-025-01567-9).

DATA DEPOSITION: We require deposition of coordinates (and, in the case of crystal structures, structure factors) into the Protein Data Bank with the designation of immediate release upon publication (HPUB). Electron microscopy-derived density maps and coordinate data must be deposited in EMDB and released upon publication. Deposition and immediate release of NMR chemical shift assignments are requested. Deposition of deep sequencing and microarray data is mandatory, and the datasets must be released **prior to** publication. To avoid delays in publication, dataset accession numbers must be supplied with the revised manuscript and appropriate release dates must be indicated at the galley proof stage.

We strongly encourage you to ask for release of ALL the deposited data now to avoid delays at publication.

Finally, as always, please pay close attention to our guidelines on statistical and methodological reporting (listed below) as failure to do so may delay the reconsideration of the revised manuscript. In particular, please provide:

- a Supplementary Figure including unprocessed images of all gels/blots in the form of a multi-page pdf file. Please ensure that blots/gels are labeled and the sections presented in the figures are clearly indicated.

- a Supplementary Table including all numerical source data in Excel format, with data for different figures provided as different sheets within a single Excel file. The file should include source data giving rise to graphical representations and statistical descriptions in the paper and for all instances where the figures present representative experiments of multiple independent repeats, the source data of all repeats should be provided.

We therefore invite you to take these points into account when revising the manuscript. In addition, when preparing the revision please:

- ensure that it conforms to our format instructions and publication policies (see below and https://www.nature.com/nature/for-authors).

- provide a point-by-point rebuttal to the full referee reports verbatim, as provided at the end of this letter.

- for any revision that includes light microscopy data, we ask our authors to please include a completed light microscopy reporting table https://www.nature.com/documents/Light_microscopy_reporting_table.xlsx to ensure the methods are described thoroughly. The table will be available to reviewers and ultimately published should the manuscript be accepted at the journal.

- provide the completed Reporting Summary (found here https://www.nature.com/documents/nr-reporting-summary.pdf). This is essential for reconsideration of the manuscript and will be available to editors and referees in the event of peer review. For more information see http://www.nature.com/authors/policies/availability.html or contact me.

When submitting the revised version of your manuscript, please pay close attention to our href="https://www.nature.com/nature-portfolio/editorial-policies/image-integrity">Digital Image Integrity Guidelines. and to the following points below:

-- that unprocessed scans are clearly labelled and match the gels and western blots presented in figures.
-- that control panels for gels and western blots are appropriately described as loading on sample processing controls
-- all images in the paper are checked for duplication of panels and for splicing of gel lanes.

EXTENDED DATA FIGURES

When re-submitting your manuscript, please ensure that any supplementary figures and tables that are crucial to the manuscript's conclusions are converted into Extended Data figures and tables to increase visibility of these data. Extended Data figures and tables are online-only (present in the online PDF and full-text HTML versions of the paper), peer-reviewed display items that provide essential background to the article but are not included in the main article due to space constraints. A maximum of ten Extended Data display items is permitted.

Finally, please ensure that you retain unprocessed data and metadata files after publication, ideally archiving data in perpetuity, as these may be requested during the peer review and production process or after publication if any issues arise.

Nature Cell Biology is committed to improving transparency in authorship. As part of our efforts in this direction, we are now requesting that all authors identified as 'corresponding author' on published papers create and link their Open Researcher and Contributor Identifier (ORCID) with their account on the Manuscript Tracking System (MTS), prior to acceptance. ORCID helps the scientific community achieve unambiguous attribution of all scholarly contributions. You can create and link your ORCID from the home page of the MTS by clicking on 'Modify my Springer Nature account'. For more information please visit please visit www.springernature.com/orcid.

This journal strongly supports public availability of data. Please place the data used in your paper into a public data repository, or alternatively, present the data as Supplementary Information. If data can only be shared on request, please explain why in your Data Availability Statement, and also in the correspondence with your editor. Please note that for some data types, deposition in a public repository is mandatory - more information on our data deposition policies and available repositories appears below.

Please submit the revised manuscript files and the point-by-point rebuttal to the referee comments using this link:

Link Redacted

*This url links to your confidential home page and associated information about manuscripts you may have submitted or be reviewing for us. If you wish to forward this email to co-authors, please delete the link to your homepage.

We hope that you will find our referees' comments and editorial guidance helpful. Please do not hesitate to contact me if there is anything you would like to discuss. Thank you again for considering NCB for this work.

Best wishes,

Melina

Melina Casadio, PhD
Senior Editor, Nature Cell Biology
ORCID ID: https://orcid.org/0000-0003-2389-2243

Reviewers' Comments:

Reviewer #1 (Remarks to the Author):

The authors have done an excellent job of addressing my technical concerns, particularly those regarding the mass spectrometry measurements of BMP lipids. Their revisions and additional clarifications have significantly strengthened the analytical rigor of the manuscript. I congratulate the authors on their compelling findings.

Reviewer #2 (Remarks to the Author):

The authors have adequately addressed my concerns regarding the previous version of their manuscript, except my question [7]. I asked the authors to compare and contrast, in the Discussion, their findings with those of Singh et al. (https://doi.org/10.1016/j.cell.2024.09.036), to point out in some detail how the approaches of Singh et al. led to insights that are different from those of their manuscript. In reply, the authors have added this phrase "Although PLD3/4 has also been proposed as an alternative to CLN5 for catalysing this final lysosomal step [43], ..." This sentence underplays the differences between the authors' study and that of Singh et al. I would like the authors to expand on these differences in their Discussion.

Reviewer #3 (Remarks to the Author):

The authors have sufficiently addressed my comments and concerns. thank you.

Reviewer #4 (Remarks to the Author):

I thank the authors for their thorough and careful responses to my previous comments. I am satisfied with how the majority of points have been addressed. However, I remain concerned regarding the quality of the model built into TM4, and do not feel this issue has been adequately resolved.

The additional densities observed in this region appear intimately associated with the helix backbone, making an assignment to a lipid or detergent molecule unlikely on geometric grounds alone. Furthermore, the morphology of these densities is not consistent with what one would typically expect for either lipid or detergent, and I therefore do not find this interpretation convincing.

I strongly recommend that the authors undertake additional cryo-EM data processing efforts specifically targeting this region before this manuscript can be considered further. This should include focused classification without alignment, local refinement centred on TM4, multibody refinement, density subtraction-based focused refinement, and careful optimisation of per-particle defocus refinement and Bayesian polishing for this region. The authors should additionally consider the ab initio reconstruction workflow of Kim, Li & Clarke (bioRxiv, 2025.09.08.674935) as it is particularly good for small proteins.

I consider this analysis particularly important because the poorly resolved density in this region may be masking a genuine conformational change that is not currently being captured; given that this is the apo state, the possibility that TM4 samples an alternative conformation in the absence of substrate would be an interesting finding in its own right and is worth investigating. If these approaches collectively fail to yield a meaningful improvement in local resolution, the authors should explicitly acknowledge in the manuscript that the registry of TM4 could not be assigned with confidence. Given that this is the apo state – and that the stabilising interactions provided by the substrate (C18/22) are absent – it is reasonable to attribute the lower local resolution in this region to inherent conformational flexibility.

Reviewer #4 (Remarks on Protocol(s)):

Yes for structural and cryo-EM analysis

GUIDELINES FOR SUBMISSION OF NATURE CELL BIOLOGY ARTICLES

READABILITY OF MANUSCRIPTS – Nature Cell Biology is read by cell biologists from diverse backgrounds, many of whom are not native English speakers. Authors should aim to communicate their findings clearly, explaining technical jargon that might be unfamiliar to non-specialists, and avoiding non-standard abbreviations. Titles and abstracts should concisely communicate the main findings of the study, and the background, rationale, results and conclusions should be clearly explained in the manuscript in a manner accessible to a broad cell biology audience. Nature Cell Biology uses British spelling.

ARTICLE FORMAT

TITLE – should be no more than 100 characters including spaces, without punctuation and avoiding technical terms, abbreviations, and active verbs..

AUTHOR NAMES – should be given in full.

AUTHOR AFFILIATIONS – should be denoted with numerical superscripts (not symbols) preceding the names. Full addresses should be included, with US states in full and providing zip/post codes. The corresponding author is denoted by: "Correspondence should be addressed to [initials]."

ABSTRACT – should not exceed 150 words and should be unreferenced. This paragraph is the most visible part of the paper and should briefly outline the background and rationale for the work, and accurately summarize the main results and conclusions. Key genes, proteins and organisms should be specified to ensure discoverability of the paper in online searches.

TEXT – the main text consists of the Introduction, Results, and Discussion sections and must not exceed 3500 words including the abstract. The Introduction should expand on the background relating to the work. The Results should be divided in subsections with subheadings, and should provide a concise and accurate description of the experimental findings. The Discussion should expand on the findings and their implications. All relevant primary literature should be cited, in particular when discussing the background and specific findings.

ACKNOWLEDGEMENTS – should be kept brief. Professional titles and affiliations are unnecessary. Grant numbers can be listed.

AUTHOR CONTRIBUTIONS – must be included after the Acknowledgements, detailing the contributions of each author to the paper (e.g. experimental work, project planning, data analysis etc.). Each author should be listed by his/her initials.

FINANCIAL AND NON-FINANCIAL COMPETING INTERESTS – the authors must include one of three declarations: (1) that they have no financial and non-financial competing interests; (2) that they have financial and non-financial competing interests; or (3) that they decline to respond, after the Author Contributions section. This statement will be published with the article, and in cases where financial and non-financial competing interests are declared, these will be itemized in a web supplement to the article. For further details please see https://www.nature.com/licenceforms/nrg/competing-interests.pdf.

REFERENCES – are limited to a total of 70 in the main text and Methods combined,. They must be numbered sequentially as they appear in the main text, tables and figure legends and Methods and must follow the precise style of Nature Cell Biology references. References only cited in the Methods should be numbered consecutively following the last reference cited in the main text. References only associated with Supplementary Information (e.g. in supplementary legends) do not count toward the total reference limit and do not need to be cited in numerical continuity with references in the main text. Only published papers can be cited, and each publication cited should be included in the numbered reference list, which should include the manuscript titles. Footnotes are not permitted.

METHODS – Nature Cell Biology publishes methods online. The methods section should be provided as a separate Word document, which will be copyedited and appended to the manuscript PDF, and incorporated within the HTML format of the paper.

Methods should be written concisely, but should contain all elements necessary to allow interpretation and replication of the results. As a guideline, Methods sections typically do not exceed 3,000 words. The Methods should be divided into subsections listing reagents and techniques. When citing previous methods, accurate references should be provided and any alterations should be noted. Information must be provided about: antibody dilutions, company names, catalogue numbers and clone numbers for monoclonal antibodies; sequences of RNAi and cDNA probes/primers or company names and catalogue numbers if reagents are commercial; cell line names, sources and information on cell line identity and authentication. Animal studies and experiments involving human subjects must be reported in detail, identifying the committees approving the protocols. For studies involving human subjects/samples, a statement must be included confirming that informed consent was obtained. Statistical analyses and information on the reproducibility of experimental results should be provided in a section titled “Statistics and Reproducibility”.

All Nature Cell Biology manuscripts submitted on or after March 21 2016, must include a Data availability statement as a separate section after Methods but before references, under the heading "Data Availability". For Springer Nature policies on data availability see http://www.nature.com/authors/policies/availability.html; for more information on this particular policy see http://www.nature.com/authors/policies/data/data-availability-statements-data-citations.pdf. The Data availability statement should include:

• Accession codes for primary datasets (generated during the study under consideration and designated as "primary accessions") and secondary datasets (published datasets reanalysed during the study under consideration, designated as "referenced accessions"). For primary accessions data should be made public to coincide with publication of the manuscript. A list of data types for which submission to community-endorsed public repositories is mandated (including sequence, structure, microarray, deep sequencing data) can be found here http://www.nature.com/authors/policies/availability.html#data.

• Unique identifiers (accession codes, DOIs or other unique persistent identifier) and hyperlinks for datasets deposited in an approved repository, but for which data deposition is not mandated (see here for details http://www.nature.com/sdata/data-policies/repositories).

• At a minimum, please include a statement confirming that all relevant data are available from the authors, and/or are included with the manuscript (e.g. as source data or supplementary information), listing which data are included (e.g. by figure panels and data types) and mentioning any restrictions on availability.

• If a dataset has a Digital Object Identifier (DOI) as its unique identifier, we strongly encourage including this in the Reference list and citing the dataset in the Methods.

We recommend that you upload the step-by-step protocols used in this manuscript to protocols.io. More details can found at https://www.protocols.io/help/publish-articles.

DISPLAY ITEMS – main display items are limited to 6-8 main figures and/or main tables. For Supplementary Information see below.

FIGURES – Colour figure publication costs $395 per colour figure. All panels of a multi-panel figure must be logically connected and arranged as they would appear in the final version. Unnecessary figures and figure panels should be avoided (e.g. data presented in small tables could be stated briefly in the text instead).

All imaging data should be accompanied by scale bars, which should be defined in the legend.
Cropped images of gels/blots are acceptable, but need to be accompanied by size markers, and to retain visible background signal within the linear range (i.e. should not be saturated). The boundaries of panels with low background have to be demarked with black lines. Splicing of panels should only be considered if unavoidable, and must be clearly marked on the figure, and noted in the legend with a statement on whether the samples were obtained and processed simultaneously. Quantitative comparisons between samples on different gels/blots are discouraged; if this is unavoidable, it has be performed for samples derived from the same experiment with gels/blots were processed in parallel, which needs to be stated in the legend.

Figures should be provided at approximately the size that they are to be printed at (single column is 86 mm, double column is 170 mm) and should not exceed an A4 page (8.5 x 11"). Reduction to the scale that will be used on the page is not necessary, but multi-panel figures should be sized so that the whole figure can be reduced by the same amount at the smallest size at which essential details in each panel are visible. In the interest of our colour-blind readers we ask that you avoid using red and green for contrast in figures. Replacing red with magenta and green with turquoise are two possible colour-safe alternatives. Lines with widths of less than 1 point should be avoided. Sans serif typefaces, such as Helvetica (preferred) or Arial should be used. All text that forms part of a figure should be rewritable and removable.

We accept files from the following graphics packages in either PC or Macintosh format:

- For line art, graphs, charts and schematics we prefer Adobe Illustrator (.AI), Encapsulated PostScript (.EPS) or Portable Document Format (.PDF). Files should be saved or exported as such directly from the application in which they were made, to allow us to restyle them according to our journal house style.

- We accept PowerPoint (.PPT) files if they are fully editable. However, please refrain from adding PowerPoint graphical effects to objects, as this results in them outputting poor quality raster art. Text used for PowerPoint figures should be Helvetica (preferred) or Arial.

- We do not recommend using Adobe Photoshop for designing figures, but we can accept Photoshop generated (.PSD or .TIFF) files only if each element included in the figure (text, labels, pictures, graphs, arrows and scale bars) are on separate layers. All text should be editable in 'type layers' and line-art such as graphs and other simple schematics should be preserved and embedded within 'vector smart objects' - not flattened raster/bitmap graphics.

- Some programs can generate Postscript by 'printing to file' (found in the Print dialogue). If using an application not listed above, save the file in PostScript format or email our Art Editor, Allen Beattie for advice (a.beattie@nature.com).

Regardless of format, all figures must be vector graphic compatible files, not supplied in a flattened raster/bitmap graphics format, but should be fully editable, allowing us to highlight/copy/paste all text and move individual parts of the figures (i.e. arrows, lines, x and y axes, graphs, tick marks, scale bars etc). The only parts of the figure that should be in pixel raster/bitmap format are photographic images or 3D rendered graphics/complex technical illustrations.

All placed images (i.e. a photo incorporated into a figure) should be on a separate layer and independent from any superimposed scale bars or text. Individual photographic images must be a minimum of 300+ DPI (at actual size) or kept constant from the original picture acquisition and not decreased in resolution post image acquisition. All colour artwork should be RGB format.

FIGURE LEGENDS – must not exceed 350 words for each figure to allow fit on a single printed NCB page together with the figure. They must include a brief title for the whole figure, and short descriptions of each panel with definitions of the symbols used, but without detailing methodology.

TABLES – main tables should be provided as individual Word files, together with a brief title and legend. For supplementary tables see below.

SUPPLEMENTARY INFORMATION – Supplementary information is material directly relevant to the conclusion of a paper, but which cannot be included in the printed version in order to keep the manuscript concise and accessible to the general reader. Supplementary information is an integral part of a Nature Cell Biology publication and should be prepared and presented with as much care as the main display item, but it must not include non-essential data or text, which may be removed at the editor's discretion. All supplementary material is fully peer-reviewed and published online as part of the HTML version of the manuscript. Supplementary Figures and Supplementary Notes are appended at the end of the main PDF of the published manuscript.

Supplementary items should relate to a main text figure, wherever possible, and should be mentioned sequentially in the main manuscript, designated as Supplementary Figure, Table, Video, or Note, and numbered continuously (e.g. Supplementary Figure 1, Supplementary Figure 2, Supplementary Table 1, Supplementary Table 2 etc.).

Unprocessed scans of all key data generated through electrophoretic separation techniques need to be presented in a supplementary figure that should be labeled and numbered as the final supplementary figure, and should be mentioned in every relevant figure legend. This figure does not count towards the total number of figures and is the only figure that can be displayed over multiple pages, but should be provided as a single file, in PDF or TIFF format. Data in this figure can be displayed in a relatively informal style, but size markers and the figures panels corresponding to the presented data must be indicated.

The total number of Supplementary Figures (not including the “unprocessed scans” Supplementary Figure) should not exceed the number of main display items (figures and/or tables (see our Guide to Authors and March 2012 editorial http://www.nature.com/ncb/authors/submit/index.html#suppinfo; http://www.nature.com/ncb/journal/v14/n3/index.html#ed). No restrictions apply to Supplementary Tables or Videos, but we advise authors to be selective in including supplemental data.

Each Supplementary Figure should be provided as a single page and as an individual file in one of our accepted figure formats and should be presented according to our figure guidelines (see above). Supplementary Tables should be provided as individual Excel files. Supplementary Videos should be provided as .avi or .mov files up to 50 MB in size. Supplementary Figures, Tables and Videos much be accompanied by a separate Word document including titles and legends.

GUIDELINES FOR EXPERIMENTAL AND STATISTICAL REPORTING

REPORTING REQUIREMENTS – We ask authors to complete a Reporting Summary that collects information on experimental design and reagents. We hope this will aid in your evaluation of the paper. The Reporting Summary can be found here https://www.nature.com/documents/nr-reporting-summary.pdf) Please note that these forms are dynamic ‘smart pdfs’ and must therefore be downloaded and completed in Adobe Reader. We will then flatten them for ease of use. If you would like to reference the guidance text as you complete the template, please access these flattened versions at http://www.nature.com/authors/policies/availability.html.

STATISTICS – Wherever statistics have been derived the legend needs to provide the n number (i.e. the sample size used to derive statistics) as a precise value (not a range), and define what this value represents. Error bars need to be defined in the legends (e.g. SD, SEM) together with a measure of centre (e.g. mean, median). Box plots need to be defined in terms of minima, maxima, centre, and percentiles. Ranges are more appropriate than standard errors for small data sets. Wherever statistical significance has been derived, precise p values need to be provided and the statistical test used needs to be stated in the legend. Statistics such as error bars must not be derived from n<3. For sample sizes of n<5 please plot the individual data points rather than providing bar graphs. Deriving statistics from technical replicate samples, rather than biological replicates is strongly discouraged. Wherever statistical significance has been derived, precise p values need to be provided and the statistical test stated in the legend.

Information on how many times each experiment was repeated independently with similar results needs to be provided in the legends and/or Methods for all experiments, and in particular wherever representative experiments are shown.

We strongly recommend the presentation of source data for graphical and statistical analyses as a separate Supplementary Table, and request that source data for all independent repeats are provided when representative experiments of multiple independent repeats, or averages of two independent experiments are presented. This supplementary table should be in Excel format, with data for different figures provided as different sheets within a single Excel file. It should be labelled and numbered as one of the supplementary tables, titled “Statistics Source Data”, and mentioned in all relevant figure legends.

--------- Please don't hesitate to contact NCB@nature.com should you have queries about any of the above requirements ---------

Version 3:

Decision Letter:

Our ref: NCB-A60924C

15th July 2026

Dear Dr. Petkevicius,

Thank you for submitting your revised manuscript "The Batten disease protein CLN8 is a stereospecific acyltransferase in bis(monoacylglycero)phosphate biosynthesis" (NCB-A60924C). It has now been seen by the original Referee #4 and their comments are below. The reviewer finds that the paper has improved in revision, and therefore we'll be happy in principle to publish it in Nature Cell Biology, pending minor revisions to comply with our editorial and formatting guidelines.

We are now performing detailed checks on your paper and will send you a checklist detailing our editorial and formatting requirements in 2-3 weeks. Please do not upload the final materials and make any revisions until you receive this additional information from us.

Thank you again for your interest in Nature Cell Biology. Please do not hesitate to contact me if you have any questions.

Sincerely,

Melina Casadio, PhD
Senior Editor, Nature Cell Biology
ORCID ID: https://orcid.org/0000-0003-2389-2243

Reviewer #4 (Remarks to the Author):

I am satisfied with these changes and am glad these suggestions were helpful. Well done on a very nice manuscript.

Reviewer #4 (Remarks on Protocol(s)):

yes for cryo-EM

INTERNAL USE ONLY AIP modules required: ["Clinical","Life Science"]

Version 4:

Decision Letter:

Dear Dr Petkevicius,

I am pleased to inform you that your manuscript, "The Batten disease protein CLN8 is a stereospecific acyltransferase in bis(monoacylglycero)phosphate biosynthesis", has now been accepted for publication in Nature Cell Biology.

Thank you for sending us the final manuscript files to be processed for print and online production, and for returning the manuscript checklists and other forms. Your manuscript will now be passed to our production team who will be in contact with you if there are any questions with the production quality of supplied figures and text.

Over the next few weeks, your paper will be copyedited to ensure that it conforms to Nature Cell Biology style. Once your paper is typeset, you will receive an email with a link to choose the appropriate publishing options for your paper and our Author Services team will be in touch regarding any additional information that may be required.

After the grant of rights is completed, you will receive a link to your electronic proof via email with a request to make any corrections within 48 hours. If, when you receive your proof, you cannot meet this deadline, please inform us at rjsproduction@springernature.com immediately.

You will not receive your proofs until the publishing agreement has been received through our system.

Due to the importance of these deadlines, we ask that you please let us know now whether you will be difficult to contact over the next month. If this is the case, we ask you provide us with the contact information (email, phone and fax) of someone who will be able to check the proofs on your behalf, and who will be available to address any last-minute problems.

If you have any questions about our publishing options, costs, Open Access requirements, or our legal forms, please contact ASJournals@springernature.com

Once your paper has been scheduled for online publication, the Nature press office will be in touch to confirm the details. An online order form for reprints of your paper is available at https://www.nature.com/reprints/author-reprints.html. All co-authors, authors' institutions and authors' funding agencies can order reprints using the form appropriate to their geographical region.

Publication is conditional on the manuscript not being published elsewhere and on there being no announcement of this work to any media outlet until the online publication date in Nature Cell Biology.

You may wish to make your media relations office aware of your accepted publication, in case they consider it appropriate to organize some internal or external publicity. Once your paper has been scheduled you will receive an email confirming the publication details. This is normally 3-4 working days in advance of publication. If you need additional notice of the date and time of publication, please let the production team know when you receive the proof of your article to ensure there is sufficient time to coordinate. Further information on our embargo policies can be found here: https://www.nature.com/authors/policies/embargo.html

Please note that *Nature Cell Biology* is a Transformative Journal (TJ). Authors may publish their research with us through the traditional subscription access route or make their paper immediately open access through payment of an article-processing charge (APC). Authors will not be required to make a final decision about access to their article until it has been accepted. <a

href="https://www.springernature.com/gp/open-research/transformative-journals"> Find out more about Transformative Journals

Authors may need to take specific actions to achieve compliance with funder and institutional open access mandates. If your research is supported by a funder that requires immediate open access (e.g. according to Plan S principles or the NIH public access policy) then you should select the gold OA route, and we will direct you to the compliant route where possible. Because authors warrant under our subscription licensing terms that they haven't committed to licensing any version of their article under a licence inconsistent with the terms of our agreement – including the applicable embargo period – publication under the subscription model isn't suitable for authors whose funders require no embargo.

To assist our authors in disseminating their research to the broader community, our SharedIt initiative provides you with a unique shareable link that will allow anyone (with or without a subscription) to read the published article. Recipients of the link with a subscription will also be able to download and print the PDF.

If your paper includes color figures, please be aware that in order to help cover some of the additional cost of four-color reproduction, Nature Portfolio charges our authors a fee for the printing of their color figures. Please contact our offices for exact pricing and details.

As soon as your article is published, you will receive an automated email with your shareable link.

If you have not already done so, we strongly recommend that you upload the step-by-step protocols used in this manuscript to protocols.io (https://protocols.io), an open online resource that allows researchers to share their detailed experimental know-how. All uploaded protocols are made freely available and are assigned DOIs for ease of citation. Protocols and Nature Portfolio journal papers in which they are used can be linked to one another, and this link is clearly and prominently visible in the online versions of both. Authors who performed the specific experiments can act as primary authors for the Protocol as they will be best placed to share the methodology details, but the Corresponding Author of the present research paper should be included as one of the authors. By uploading your Protocols onto protocols.io, you are enabling researchers to more readily reproduce or adapt the methodology you use, as well as increasing the visibility of your protocols and papers. You can also establish a dedicated workspace to collect your lab Protocols. Further information can be found at https://www.protocols.io/help/publish-articles.

You can use a single sign-on for all your accounts, view the status of all your manuscript submissions and reviews, access usage statistics for your published articles and download a record of your refereeing activity for the Nature Portfolio.

Nature Cell Biology encourages authors presenting evidence for cell, biological, molecular, and genetic interactions to consider communicating these findings using Biofactoid (https://biofactoid.org/). This tool helps users share a searchable representation of interactions (e.g. binding, gene expression, post-translational modification) between genes, gene products, or chemicals. Information added to Biofactoid, with author attribution, is shared on social media and public databases, such as Pathway Commons, where it can be discovered and analyzed in the context of a large and growing corpus of knowledge.

Please feel free to contact us if you have any questions.

With kind regards,

Melina Casadio, PhD
Senior Editor, Nature Cell Biology
ORCID ID: https://orcid.org/0000-0003-2389-2243

Click here if you would like to recommend Nature Cell Biology to your librarian
http://www.nature.com/subscriptions/recommend.html#forms

** Visit the Springer Nature Editorial and Publishing website at www.springernature.com/editorial-and-publishing-jobs for more information about our career opportunities. If you have any questions please click here.**

Open Access This Peer Review File is licensed under a Creative Commons Attribution 4.0 International License, which permits use, sharing, adaptation, distribution and reproduction in any medium or format, as long as you give appropriate credit to the original author(s) and the source, provide a link to the Creative Commons license, and indicate if changes were made.
In cases where reviewers are anonymous, credit should be given to 'Anonymous Referee' and the source.
The images or other third party material in this Peer Review File are included in the article's Creative Commons license, unless indicated otherwise in a credit line to the material. If material is not included in the article's Creative Commons license and your intended use is not permitted by statutory regulation or exceeds the permitted use, you will need to obtain permission directly from the copyright holder.
To view a copy of this license, visit https://creativecommons.org/licenses/by/4.0/

Response to reviewers

Sheokand, Lacabanne, James *et al*, 2026

Reviewer #1 (Remarks to the Author):

In this manuscript, Pradeep K. Sheokand et al. present compelling data identifying CLN8 as a stereospecific acyltransferase that converts S,S-glycerophosphoglycerol (PGP) to S,S-lysophosphatidylglycerol (LPG), a substrate precursor for bis(monoacylglycero)phosphate (BMP) biosynthesis. Using high-resolution cryo-electron microscopy (cryo-EM), the authors reveal a ping-pong acyl transfer mechanism for CLN8. Furthermore, they demonstrate that mutations associated with CLN8-related Batten disease resulted in a graded loss of enzymatic activity, correlating directly with reduced BMP levels and clinical severity. These findings offer robust evidence that disrupted BMP biosynthesis is a primary driver of Batten disease. Importantly, the study shows that exogenous S,S-LPG supplementation can restore BMP level both in vitro and in vivo, leading to modest improvements in neurological phenotypes in a cln8 mutant zebrafish model. Given the underexplored role of BMP in lysosomal regulation, identifying CLN8's specific function is a significant advancement for the field.

While the mass spectrometry (MS)-based lipidomics is generally robust, refinements regarding isomer resolution and quantification transparency are necessary to strengthen the study's rigor. I strongly support the publication of this work pending these revisions.

We thank the reviewer for their thoughtful and constructive comments.

1. The study's key rescue experiments rely on distinguishing S,S-LPG from R,rac-LPG, and resolving BMP positional isomers (2,2'-, 2,3'-, 3,3'- acyl configurations). While LC-MS/MS is used for detection, the manuscript lacks details on whether these stereoisomers/positional isomers achieve baseline separation under the described chromatographic conditions. For example, Supplementary Table 5 lists MRM transitions but does not provide retention time windows or chromatograms demonstrating resolved peaks for S,S-LPG vs. other isomers. Because ion ionization efficiency can vary between stereoisomers, the authors should provide calibration curves using purified synthetic standards for each isomer to correct for potential quantification bias. This is particularly critical for the in vivo rescue experiments (Fig. 4d) and BMP isomer analysis in Cln8R24G mice (Fig. 3e), where conclusions about residual enzymatic activity depend on relative isomer abundance.

We thank the reviewer for raising these important points. We agree that resolving BMP positional *and* stereoisomers remains a major analytical challenge in the field. We have therefore revised the manuscript to state more clearly what our current methods can and cannot resolve. **Specifically, we now distinguish conclusions that are directly supported by our analytical capabilities from questions that remain limited by our current methodology and fall outside the scope of this study.**

a) Distinguishing *S,S*-LPG from *R,rac*-LPG in rescue experiments

As the reviewer notes, one of the key findings of our study is the stereospecific ability of *S,S*-LPG, but not *R,rac*-LPG, to restore BMP synthesis in CLN8-deficient cells and in vivo. <u>Importantly, this conclusion does not depend on chromatographic separation of *S,S*-LPG and *R,rac*-LPG by our LC-MS method.</u> Rather, the two rescue compounds were generated by independent organic synthesis routes designed to yield the appropriate stereochemical products. The synthesis and purification were performed by specialist chemistry providers with extensive expertise in lipid synthesis, and chiral supercritical fluid chromatography was used during compound production to confirm high (99%) enantiomeric purity.

We have now clarified in the Methods that, under our LC-MS conditions, synthetic *S,S*-LPG and *R,rac*-LPG co-elute and produce indistinguishable signal intensities when analysed as purified standards. For further clarity, in the new Extended Data Fig. 9d, we present traces of *S,S*-LPG and *R,rac*-LPG standards analysed at identical amounts using our LC-MS system. <u>Thus, our LC-MS method was not used to assign the stereochemical identity of these compounds</u>. Instead, stereochemistry was established by organic chemical synthesis and chiral analytical validation, while the biological conclusion comes from the markedly different rescue activities of the two independently synthesised compounds, as presented in Fig. 4.

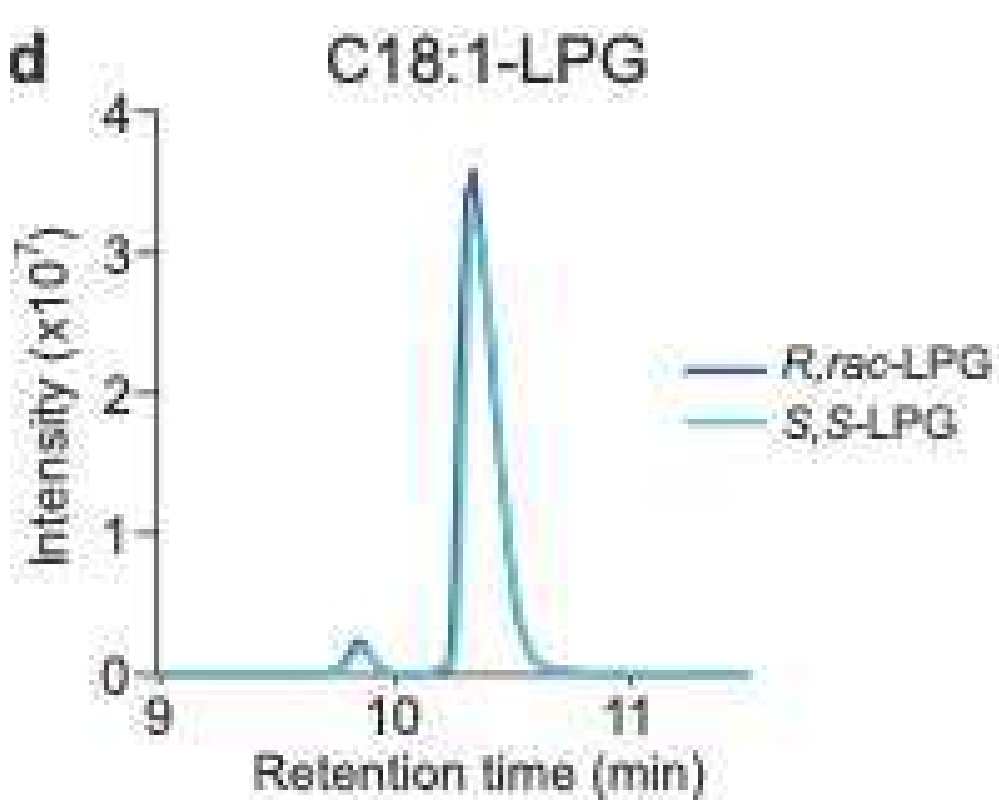

Extended data Fig. 9d. *Synthetic R,rac- and S,S-LPG standards used in rescue experiments, analysed at equal amounts using our LC-MS workflow.*

b) Resolving BMP acyl positional isomers

We also agree that BMP acyl positional isomers are important in BMP biology. However, direct resolution and quantification of the full range of BMP positional isomers (2,2' vs 2,3' vs 3,3') falls outside the scope of the present study. The focus of this manuscript is the biochemical function of CLN8 in the BMP synthesis pathway. Our data identify CLN8 as an *S,S*-GPG acyltransferase and show that its direct product is LPG. Accordingly, we focused our analytical validation on resolving LPG positional isomers. **We achieved baseline chromatographic separation of 2-LPG and 3-LPG, enabling us to demonstrate that CLN8 preferentially produces 2-LPG species.** These data support the conclusion that CLN8 generates the 2-LPG intermediate that is subsequently used by CLN5 for BMP synthesis.

By contrast, the final conversion of LPG into BMP is catalysed by CLN5. Therefore, the precise positional isomer composition of the BMP species produced by CLN5 is a distinct biochemical question that will require dedicated future work focused on CLN5 biochemistry. **To avoid overinterpretation, we have revised the relevant sections of the manuscript to clarify that references to BMP positional isomers are based on prior literature and current understanding of BMP chemistry, rather than direct positional isomer assignment in our LC-MS experiments. We have also updated the Methods to state explicitly that positional BMP isomers were not resolved by our LC-MS workflow.**

In addition, we now include a new observation relevant to the reviewer's point. The synthetic *S,S*-LPG used in our rescue experiments corresponds not to the CLN8 product, *S,S*-2-acyl-LPG, but to the more stable ***sn*-3-acylated isomer, *S,S*-3-acyl-LPG (see new Extended Data Fig. 9d above).** However, when this *S,S*-3-acyl-LPG was added to CLN5/8 double-knockout cells, in which LPG accumulates because the downstream CLN5-dependent conversion is blocked (see Extended Data Fig. 9c), **we detected a substantial *sn*-2 acylated LPG pool (new Extended Data Fig. 9e).** This indicates that, after cellular uptake, LPG can undergo positional acyl chain migration independently of CLN5 and CLN8. Similarly, this suggests that the acyl chain positions in BMP may be dynamically adjusted independently of its biosynthetic enzymes, CLN5 and CLN8. This finding further suggests that the biological activity of an exogenous LPG rescue compound may not require a fixed input acyl configuration, because cellular remodelling can alter the positional distribution after uptake. Identifying the enzymes or chemical mechanisms responsible for this acyl migration will require a dedicated study and is beyond the scope of the present manuscript.

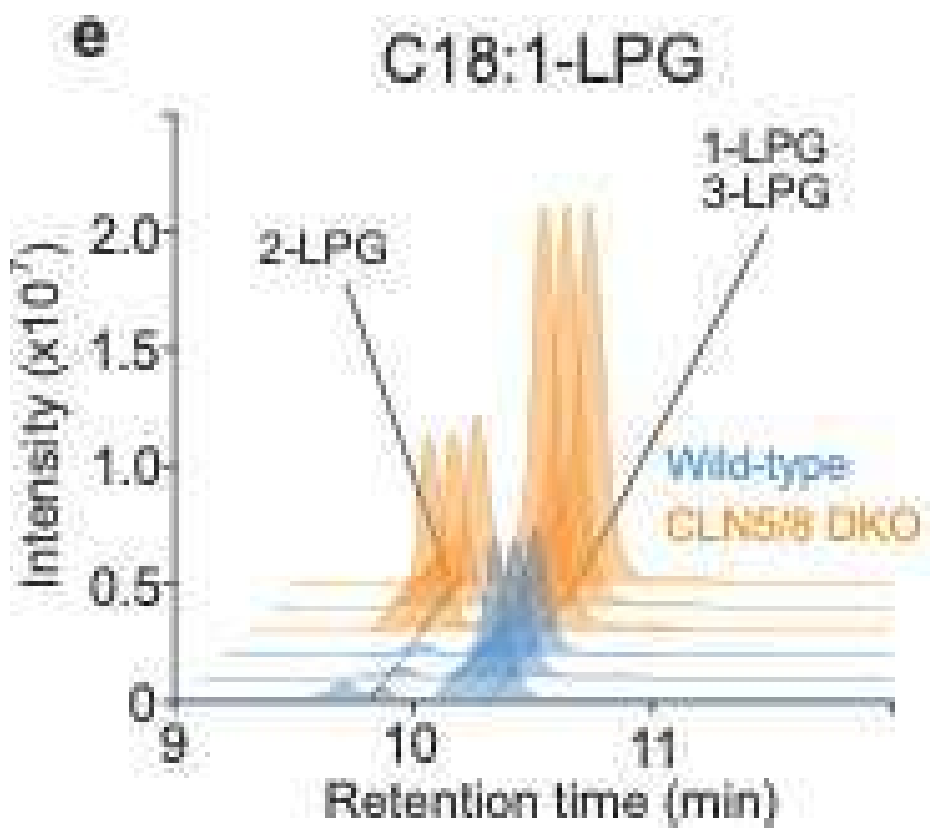

Extended data Fig. 9e. *LC-MS/MS chromatograms of C18:1-LPG in control and CLN5/8 DKO HeLa cells treated with 10 µM S,S-LPG for 6 h, resolved against positional isomer standards.*

New text in the manuscript:

Interestingly, although the chemically synthesised S,S-C18:1-LPG used in our rescue experiments is acylated at the sn-3 position, its accumulation in CLN5/8 DKO HeLa cells was accompanied by the substantial appearance of an sn-2-acylated C18:1-LPG pool (Extended Data Fig. 9d,e). This suggests that cells possess as-yet-unknown mechanisms for LPG acyl chain rearrangement, and potentially BMP acyl chain rearrangement, that operate independently of the CLN8-CLN5 biosynthetic pathway.

c) BMPs in Cln8^{R24G} mice

We understand the reviewer's concern to relate not to BMP isomers, but to potential differences in ionisation efficiency between different BMP species, particularly 18:1-18:1 and 22:6-22:6 BMP, and how this may affect their relative abundance in wild-type and Cln8^{R24G} mice. Because a chemical standard for 22:6-22:6 BMP is not commercially available, **we addressed this point by repeating the analysis on a subset of samples (n=3 per group) using an independent untargeted LC-MS lipidomics workflow on an Orbitrap instrument, measuring intact BMP molecules as $[M-H]^-$ ions in negative mode (Reviewer Fig. 1; see PMID: 39970228 for methodology).** This independent analytical platform produced near-identical relative comparisons between the measured BMP acyl species and those obtained with the LC-MS/MS fragmentation method used in the manuscript. We therefore believe that our conclusions regarding the relative abundance of BMP species in Cln8^{R24G} mice, and across our other in vivo experiments, are robust.

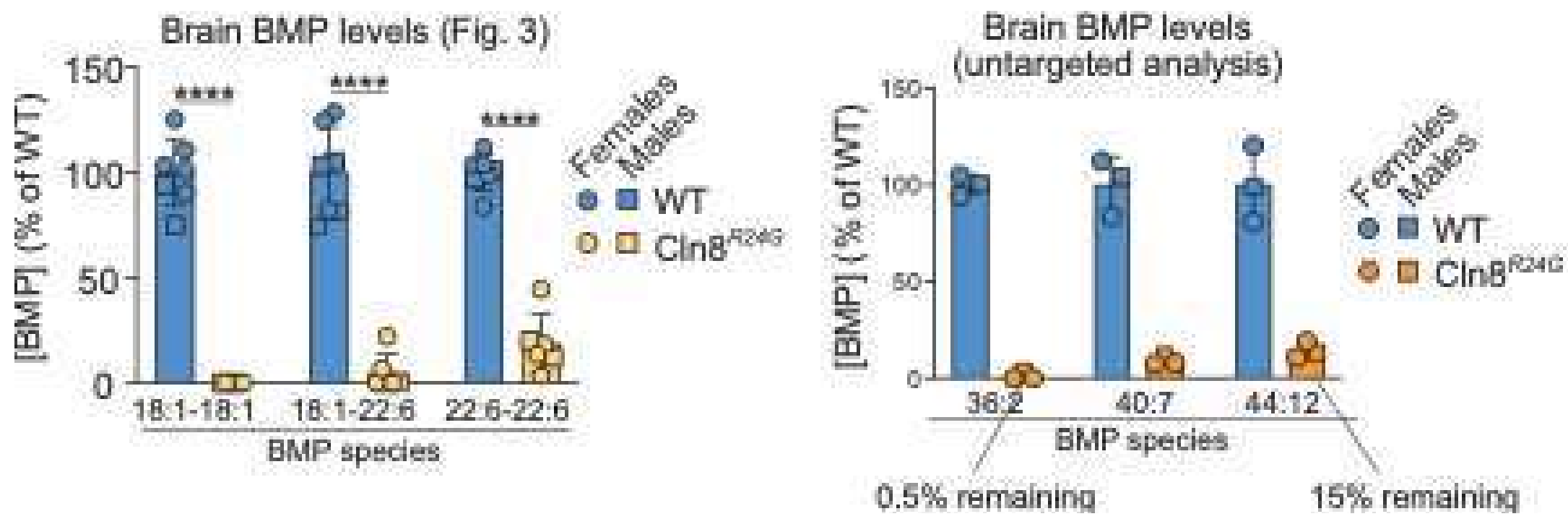

Reviewer Fig. 1. Relative abundance of the indicated BMP species in wild-type and Cln8^{R24G} mouse brains, quantified using our targeted LC-MS/MS method presented in Fig. 3 and independently reanalysed in a subset of samples (n = 3) using an untargeted LC-MS method.

2. The manuscript identifies 2-acyl-LPG as the CLN8 product and 2,2’-BMP as the mature end product. However, the MS/MS-based assignment of BMP positional isomers (e.g., Extended Data Fig. 2h) requires more explicit validation. The assignment of 2,2’ vs. 2,3’ isomers currently relies on retention time comparison. The authors should provide MS/MS spectra of synthetic BMP positional isomers to highlight diagnostic fragment ions that distinguish these configurations. Given that BMP isomers differ in biological functon (such as resistance to lysosomal hydrolysis), unambiguous assignment is essential for the study's mechanistic claims.

We agree that we identify 2-*S,S*-acyl-LPG as the product of CLN8, and that BMP positional isomers represent the biologically important endpoint of the pathway in which CLN8 acts. However, the final step that defines the positional configuration of BMP occurs in the lysosome and is catalysed by CLN5, downstream of the ER-localised reaction catalysed by CLN8. We therefore consider the precise positional configuration of the BMP products generated by CLN5 to be beyond the scope of this CLN8-focused manuscript, and better suited to studies specifically addressing CLN5 enzymology.

We have addressed this issue in our response to point 1b and have revised the manuscript to avoid overstatement. Specifically, we no longer present 2,2′-BMP as a directly assigned end product of the pathway. Instead, we describe this assignment as an inference based on previous literature and current understanding of BMP chemistry. We have also clarified in the Methods that our LC-MS/MS method does not unambiguously distinguish BMP positional isomers such as 2,2′-BMP and 2,3′-BMP. Importantly, this does not affect the mechanistic conclusions of our study, which concern the enzymatic function of CLN8 and its position within the BMP biosynthetic pathway.

3. The rescue experiments with S,S-LPG are a highlight of the paper, yet there is a slight imbalance in the data provided for each model. In mouse models, the authors

provide BMP restoration data but lack accompanying phenotypic/behavioral rescue data. In zebrafish models, the authors show modest neurological improvement but lack biochemical data confirming BMP restoration levels. For both models, the authors should indicate the extent to which BMP and locomotor activity are restored relative to wild type baseline. This is necessary to evaluate the true therapeutic potential of S,S-LPG supplementation.

We thank the reviewer for this important point. We agree that comparison with wild-type baselines is essential for defining the extent of rescue, and that the rationale for the analyses performed in each model was not sufficiently clear in the original manuscript. **We have therefore strengthened the in vivo rescue data in both models and clarified why zebrafish larvae are well suited for an initial assessment of *S,S*-LPG-mediated rescue of neurological phenotypes.**

First, we repeated the mouse rescue experiment using a higher LPG dose and included BSA-injected wild-type mice as an additional baseline control. These data show that *S,S*-LPG supplementation restores the 18:1-18:1 BMP species in CLN8-deficient mice to <u>levels above those detected in wild-type controls</u>.

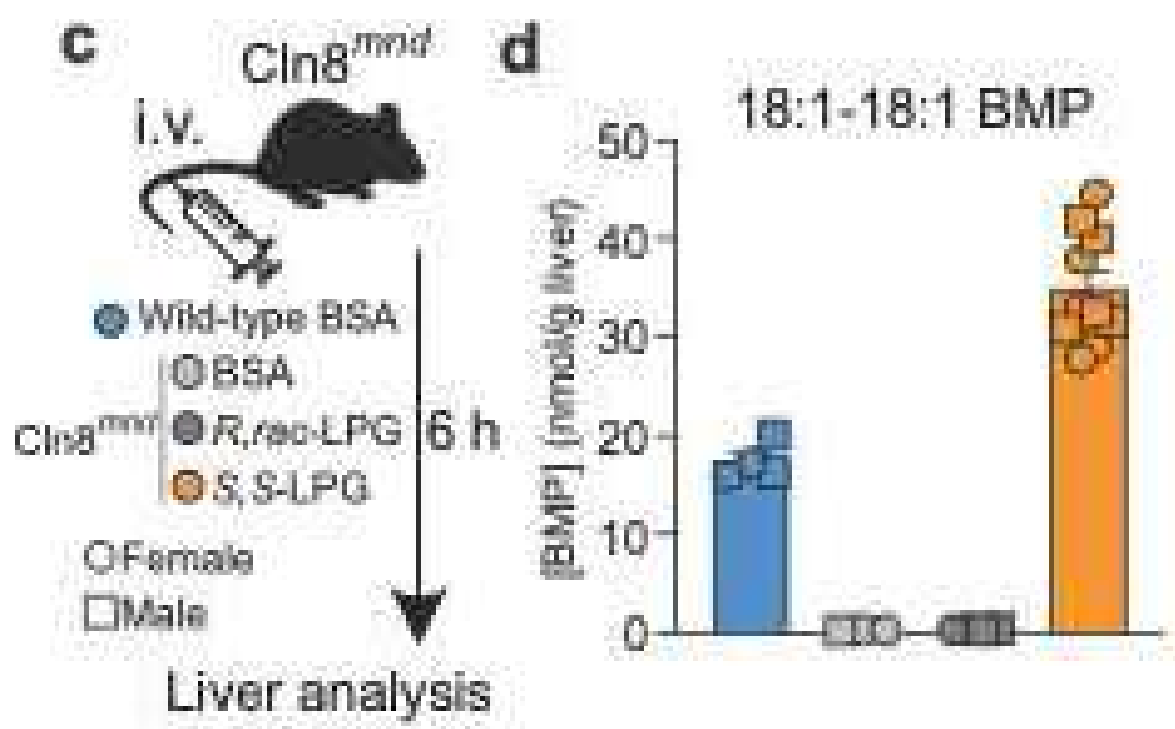

Revised Figure 4c.

Second, following the reviewer's suggestion, we have added a wild-type zebrafish group to the behavioural rescue experiment. This allows the effect of *S,S*-LPG treatment in cln8 mutant zebrafish to be assessed relative to the wild-type locomotor baseline, rather than only relative to untreated mutants.

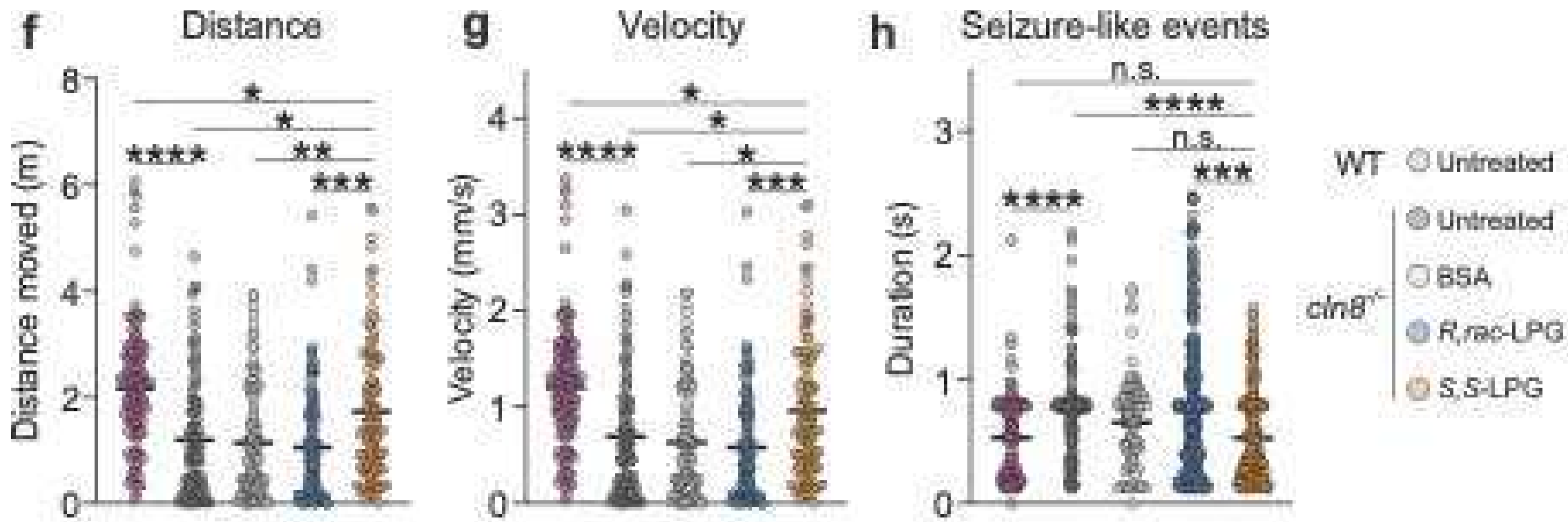

Revised Figures 4f-h.

We have also analysed BMP formation in zebrafish larvae using our targeted LC-MS/MS methodology. At the endpoint analysed, 18:1–18:1 BMP was below the detection limit in wild-type larvae at 120 hpf, preventing robust quantitative comparison with the physiological baseline. However, 18:1-18:1 BMP became detectable in *S,S*-LPG-treated, but not *R,rac*-LPG-treated, *cln8* mutant larvae. Although this signal is close to the detection limit, it is consistent with stereospecific engagement of the BMP biosynthetic pathway in vivo and supports the functional rescue observed in the same model. **We have added this point to the Results and interpret it cautiously:**

Using our targeted LC-MS/MS methodology, 18:1-18:1 BMP was below the detection limit in wild-type larvae at 120 hpf, but was detectable in S,S-LPG-treated, but not R,rac-LPG-treated, cln8-/- larvae (Extended Data Fig. 9h). Although this limits quantitative assessment of BMP restoration in zebrafish, it is consistent with pathway engagement in vivo. The low detectability of 18:1-18:1 BMP at 120 hpf may reflect developmental regulation of BMP synthesis, consistent with higher cln8 mRNA expression at 0 and 72 hpf than at 120 hpf, and the onset of zebrafish blood-brain barrier formation from approximately 72 hpf may allow greater S,S-LPG uptake during the early treatment window that is not captured by our 120 hpf lipidomics snapshot.

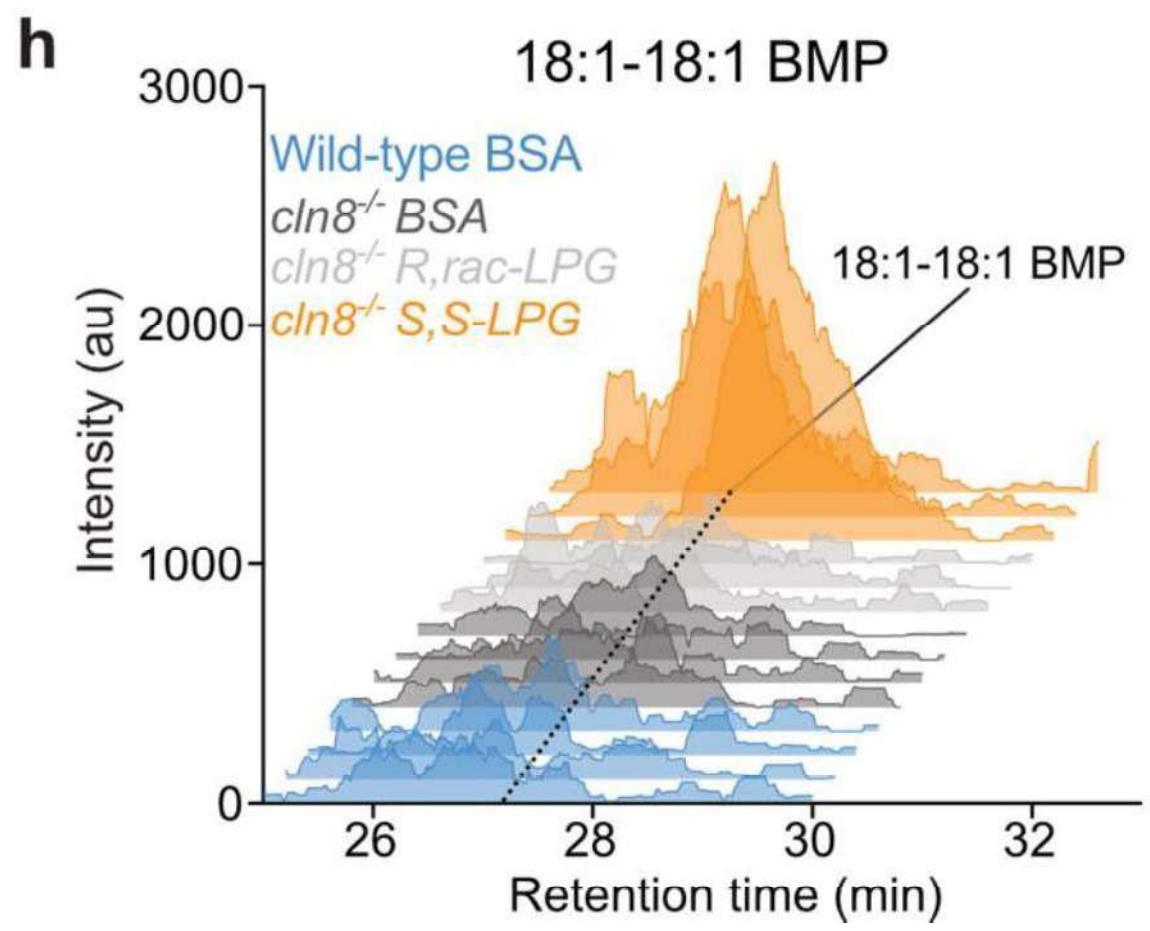

Extended Data Fig. 9h. LC-MS/MS chromatograms of 18:1-18:1 BMP in larvae at 120 hpf: BSA-treated wild-type larvae (n = 4) and *cln8*$^{-/-}$ larvae treated with BSA (n = 4), *R,rac*-LPG (n = 3) or *S,S*-LPG (n = 3).

We would also like to clarify the intended scope of these experiments. The *S,S*-LPG rescue studies were designed as proof-of-concept experiments to test the biochemistry of the pathway in vivo and its relevance to CLN8 disease. They were not intended to represent a fully optimised therapeutic dosing regimen. **This is now emphasised in the Discussion:**

The therapeutic potential of S,S-LPG now justifies further rescue studies in mammalian CLN8-deficient models. Conceptually, S,S-LPG provides a precursor-bypass strategy: it replaces the missing direct product of CLN8 and allows the endogenous CLN5-dependent pathway to generate BMP. However, our pilot mouse studies indicate that intravenous administration of BSA-conjugated S,S-C18:1-LPG results in only minimal 18:1-18:1 BMP formation in the brain over the acute treatment window, suggesting limited blood-brain barrier permeability of C18:1-LPG in adult mice. Optimising the molecular properties of S,S-LPG, including acyl chain length and unsaturation, will therefore be important for improving in vivo delivery, brain penetration and rescue efficacy. Direct S,S-BMP supplementation may also be considered, although it is likely to face additional challenges related to aqueous solubility, membrane insertion, cellular uptake and lysosomal delivery.

The rescue experiments presented here should therefore be viewed as proof-of-concept studies showing that BMP production can be pharmacologically restored with functional benefit, rather than as optimised reconstitution of endogenous BMP diversity and function. Similarly, the exogenous lipids and tracers used here were applied at concentrations designed to establish enzymatic specificity and pathway connectivity, not to define physiological pathway flux. Future studies are therefore needed to define whether BMP precursors can be modified to improve blood-brain barrier penetration; to determine the turnover of endogenous brain BMP pools; and to quantify flux through the BMP biosynthetic pathway under physiologically relevant conditions.

Finally, we now present preliminary observations indicating that the current *S,S*-LPG formulation has limited brain exposure in adult mice, as shown by the minimal recovery of 18:1-18:1 BMP in brain after intravenous administration (**Extended Data Fig. 9f**). This is relevant to the interpretation of the zebrafish rescue data, because the zebrafish experiments were performed at 5 days post-fertilisation, when the blood-brain barrier is still maturing: it begins to form at approximately 3 days post-fertilisation and matures only by around 10 days post-fertilisation (PMID: 30862465). Thus, continuous administration from early development may allow compound access during a permissive window, helping to explain the improvement in neurological phenotypes observed at 5 days post-fertilisation. **We now discuss these points explicitly in the manuscript.** Achieving robust, durable and translationally relevant rescue of Batten disease phenotypes in mice will require further compound optimisation, pharmacokinetic and pharmacodynamic analysis, and dedicated dosing studies. This is an important next step and is the focus of ongoing work in our laboratory.

4. The manuscript proposes stereochemically defined S,*S*-LPG as a therapeutic strategy. Since impaired BMP synthesis is the core defect, the discussion would benefit from a brief comparison of the chemical and cellular advantages of supplementing with the LPG precursor versus the BMP end-product (e.g., bioavailability, stability, or cellular uptake).

We thank the reviewer for this helpful suggestion. **We have expanded the Discussion to explain the rationale for using *S,S*-LPG as a BMP precursor rather than supplementing the BMP end-product directly.**

Specifically, we now explain that *S,S*-LPG provides a precursor-bypass strategy because it replaces the direct product of CLN8 and can be converted into BMP by the endogenous CLN5-dependent pathway. In contrast, direct *S,S*-BMP supplementation may face additional challenges related to aqueous solubility, membrane insertion, cellular uptake and lysosomal delivery. We therefore present *S,S*-LPG as a tractable proof-of-concept precursor, while noting that both LPG analogues and direct BMP delivery strategies will require optimisation.

5. The manuscript would benefit from an expanded discussion section that better contextualizes the CLN8 pathway within the broader framework of cellular physiology. Specifically, I recommend further elaborating on the cellular origins of GPG, the mechanisms underlying LPG membrane transport, and related processes.

We thank the reviewer for this suggestion. **We have expanded the Discussion to place the CLN8 reaction more clearly within the broader BMP biosynthetic pathway, including likely precursor origins and unresolved trafficking steps.**

[Redacted]

6. For the sake of non-specialist readers, please add positional labels to the acyl chains in the chemical structure figures where appropriate.

We thank the reviewer for this suggestion. **We have now included Extended Data Fig. 2e, illustrating the relevant BMP and LPG acyl positional isomers for non-specialist readers.** We chose this approach rather than adding further labels to the existing chemical structures, as the original panels were already information-dense and additional annotations reduced clarity.

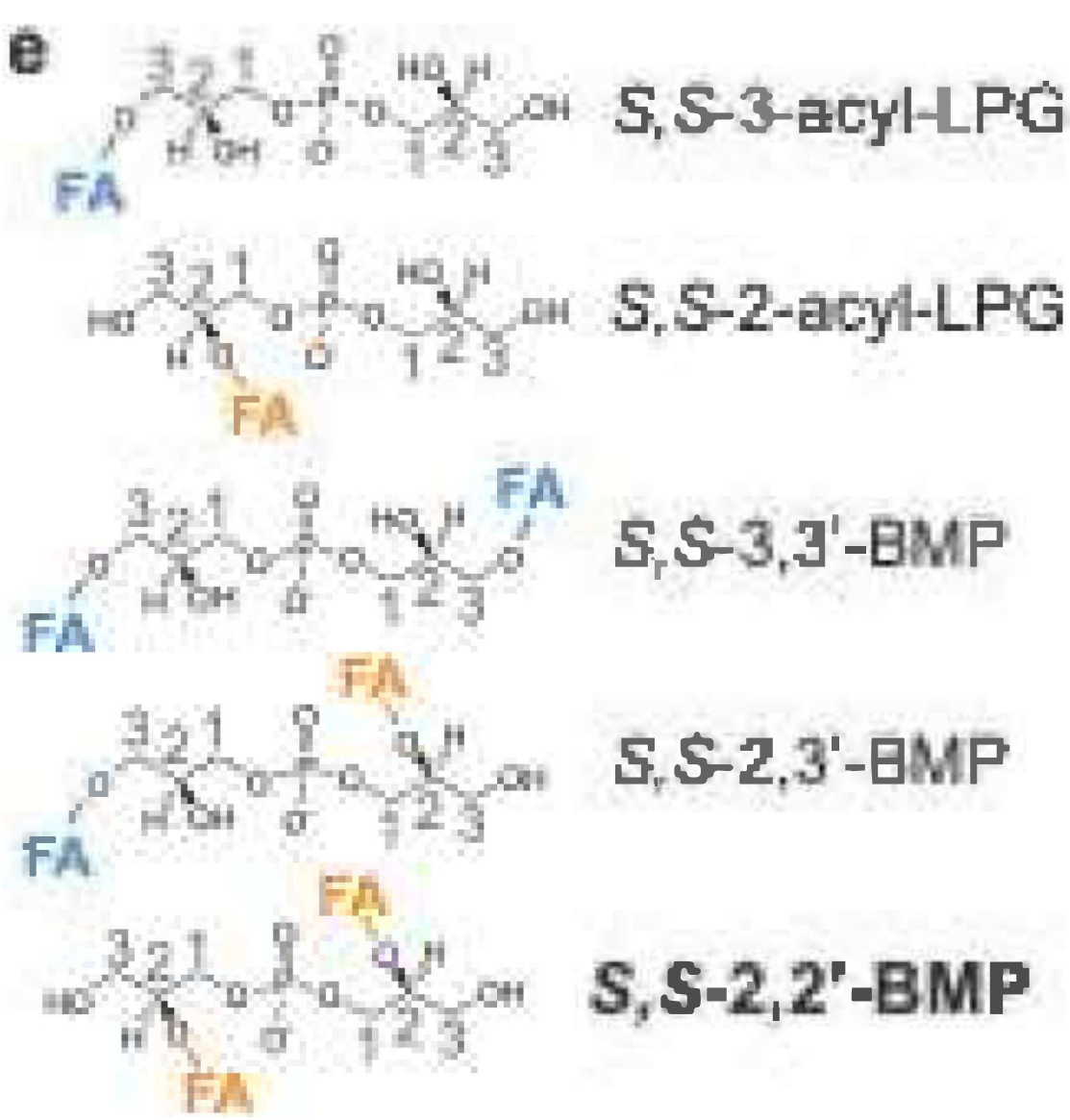

Extended Data Fig. 2e, *Structures of the possible acyl positional isomers of S,S-LPG and S,S-BMP, with acyl positions indicated by numbers adjacent to each carbon. Because of the S stereochemistry of the glycerol backbone, the phosphate-linked glycerol carbon is assigned as sn-1, and the distal glycerol carbon as sn-3. The physiological 2,2'-BMP isoform is highlighted in bold.*

Reviewer #1 (Remarks on Protocol(s)):

Please refer to my related comments in the general comments section.

Reviewer #2 (Remarks to the Author):

Sheokand et al. present a large body of data relating to the enzymatic activity of CLN8 and its involvement in Batten disease. They have identified its substrate, and used cryoelectron microscopy to derive its three-dimensional structure. With this structure in hand, the authors have examined a number of mutations in the CLN8 gene associated with forms of Batten disease differing in severity, and have been able to establish that the different mutations affect the catalytic mechanism of CLN8 in different ways. Further, the authors have examined two Cln8-deficient mouse models to establish the consequences of Cln8 deficiency on the BMP metabolic pathway. In the final section of the manuscript, the authors show that S,S-LPG supplementation restores BMP synthesis in CNL8-deficient HeLa cells and mice, and that this treatment improves the locomotion of cln8-mutant zebrafish. These results warrant further studies to establish whether S,S-LPG supplementation provides clinical benefits to Batten disease patients carrying CLN8 mutations.

Comments

[1] The title of the manuscript contains three abbreviations that are not too common. The more abbreviations in the title of a research paper, the fewer readers it attracts, and the fewer times it gets cited. In particular for a journal with a such wide readership as Nature Cell Biology, the authors should strive to make the title of their research paper intelligible and appealing for the wider research community.

We thank the reviewer for this helpful suggestion. We agree that the original title contained too many specialised abbreviations for the broad readership of *Nature Cell Biology*. **We have therefore revised the title to improve accessibility and reduce technical density, while retaining the central mechanistic finding that CLN8 catalyses a defined acylation step in BMP biosynthesis.**

[2] The writing of the Main section can be improved. Again, the wide readership of Nature Cell Biology includes many who are not specialists in BMP metabolism (second paragraph), and the link with Batten disease (first paragraph) may not be obvious for them.

We thank the reviewer for this helpful suggestion. **We have revised the opening section to introduce the connection between BMP metabolism and Batten disease more clearly for non-specialist readers.**

[3] On page 8, penultimate paragraph, results are presented from administering S,S-LPG or R,rac-LPG to homozygous Cln8mnd mice (Fig. 4 c and d). Exogenous S,S-LPG resulted in the appearance of BMP in the mutant mice. However, for therapeutic

purposes, it is critical to establish to what extent a treatment restores a deficit relative to its physiological value. So, it would be good to extend Fig. 4 d with the BMP level of untreated WT mice of the same strain background as the homozygous Cln8mnd mice.

We thank the reviewer for raising this point. As already addressed in response to **Reviewer 1, point 3, we strengthened the in vivo rescue experiment by adding BSA-injected wild-type littermates as a baseline control group and administering LPG at a higher dose.** The revised data show that *S,S*-LPG treatment restores the 18:1-18:1 BMP species in Cln8^{mnd} mice to levels above those detected in wild-type controls.

[4A] On page 8, last paragraph, results are presented from administering S,S-LPG or R,rac-LPG to cln8 mutant zebrafish larvae. S,S-LPG lessened the pathology of the mutant larvae (Figures 4 f, g, and h). Nevertheless, also for this experiment, the efficacy of the therapeutic intervention can only be properly assessed when the outcomes are compared to physiological standards. Therefore, it would be good to extend Figures 4 f, g, and h with the corresponding data of untreated wild-type larvae of the same strain as the mutants.

We thank the reviewer for this point. **We have added the corresponding wild-type larval groups to the zebrafish rescue experiments, as addressed to Reviewer 1 point 3.**

[4B] Have the authors determined whether S,S-LPG supplementation restores the levels of BMP in cln8 mutant zebrafish larvae?

We thank the reviewer for raising this point. As already addressed in response to **Reviewer 1, point 3,** we have now analysed BMP in zebrafish larvae using our targeted LC-MS/MS methodology. At the endpoint analysed, 18:1-18:1 BMP was below the detection limit in wild-type 120 hpf larvae, preventing robust quantitative comparison with the physiological baseline. However, 18:1-18:1 BMP became just detectable in *S,S*-LPG-treated, but not R,rac-LPG-treated, *cln8* mutant larvae. Although this measurement is close to the detection limit, it is consistent with stereospecific engagement of the BMP biosynthetic pathway in vivo and supports the functional rescue observed in the same model. **We have added this point to the Results and interpret it cautiously.**

[5] The bottom panels of Extended Data Fig. 4 are very small, and their lettering is challenging to read. It would be good if these panels were replaced with larger versions.

We thank the reviewer for pointing this out. **We have enlarged the lower panels of Extended Data Fig. 4 and adjusted the lettering to improve readability.**

[6] Discussion: In a previous study (DOI: 10.1126/sciadv.adr3723), the authors used the AlphaFold2 model of CLN8. In would be good to compare and contrast the AlphaFold2 model with the cryoelectron microscopy-derived structure of CLN8 presented here. Does the empirical model differ much from the in silico model?

We thank the reviewer for this suggestion. We have now included a comparison between the previously reported AlphaFold2 model (DOI: 10.1126/sciadv.adr3723) and our cryo-EM structures.

We added the following in the Methods section, under 'Model building, validation, and structural analysis':

The experimentally determined CLN8 structure closely matches the previously reported AlphaFold2 model, with an overall RMSD of 0.53 Å (all-atom RMSD calculated using PyMOL), indicating that the global fold was accurately predicted.

[7] Recently, Singh et al. (https://doi.org/10.1016/j.cell.2024.09.036) proposed a metabolic pathway for BMP synthesis that involves lysosomal phospholipases D3/D4 (PLD3/4). The authors of the current manuscript are encouraged to expand their Discussion, comparing and contrasting it with their own work. It is important that the authors point out in some detail how the approaches of Singh et al. led to insights that are different from those of the current manuscript. Alternatively, the authors could provide a synthesis of both studies, showing how the roles of PLD3/4 and CLN8 may be complementary, following the wider scope of the Discussion of Bulfon et al. (https://doi.org/10.1038/s41467-024-54213-1). With the current knowledge, would it be is possible to arrive at a unified vision of BMP synthesis, or are there outstanding issues to be resolved experimentally?

We thank the reviewer for raising this important point. The relationship between the CLN5-dependent pathway discussed here and the PLD3/4-dependent model proposed by Singh et al. is an important unresolved issue in the BMP field.

We would like to emphasise that the CLN8 reaction identified in our study lies upstream of the final lysosomal BMP-generating step. In principle, CLN8-dependent production of *S,S*-LPG could be compatible with different proposed downstream routes for BMP formation, including models involving CLN5 or PLD3/4. However, our data, together with findings from other laboratories, support a pathway in which CLN5 uses *S,S*-LPG as the immediate substrate for BMP synthesis. We have therefore kept the Discussion focused on the CLN8-CLN5 pathway, which is the model most directly supported by our experimental data.

To address the reviewer's point, we have nevertheless added a sentence to the Discussion acknowledging that conflicting reports exist regarding the final step of BMP synthesis, particularly with respect to proposed roles for CLN5 and PLD3/4.

We also feel that clarity on this point is particularly important for the Batten disease field. BMP metabolism is emerging as a central aspect of Batten disease biology, but many researchers in this rare disease community are not lipid biochemists. Conflicting models of BMP biosynthesis can therefore create uncertainty about which disease mechanisms and therapeutic routes should be prioritised**. For this reason, we present a clear, data-supported pathway centred on Batten disease proteins, rather than giving equal weight to alternative models that are less directly connected to the genetic and biochemical framework developed in this study.**

[Redacted]

Finally, the accompanying manuscript by Breithofer et al. (CLN8 enables a non-canonical phospholipid synthesis pathway) contains a detailed discussion of the PLD3/4 model.

Reviewer #2 (Remarks on Protocol(s)):

The methods have been adequately described.

Reviewer #3 (Remarks to the Author):

The authors provide a comprehensive biochemical, structural, and in vivo characterization of CLN8, definitively establishing it as the enzyme responsible for stereospecific glycerophosphoglycerol (GPG) acylation during bis(monoacylglycero)phosphate (BMP) biosynthesis. By integrating enzymology, stereochemically defined lipid substrates, high-resolution cryo-EM structures,

disease-associated mutations, and multiple animal models, the study convincingly links impaired BMP synthesis to the pathogenesis of CLN8-associated Batten disease. Overall, this work represents a technical and conceptual advance in the fields of lysosomal lipid metabolism and neurodegenerative disease.

Comments/Suggestions

1. While the enzymatic and tracer data convincingly establish GPG as a bona fide substrate for CLN8, additional discussion of the physiological sources, abundance, and turnover of S,S-GPG would help contextualize how this pathway operates under basal versus stress or disease conditions.

We thank the reviewer for this helpful suggestion. **We have expanded the Discussion to include possible physiological sources, abundance and turnover of *S,S*-GPG, and how these may influence pathway activity under basal and disease conditions**.

2. The proposed model requires trafficking of CLN8-generated S,S-LPG from the ER to the lysosome for CLN5-mediated condensation. This step remains mechanistically unresolved. A brief discussion of potential trafficking routes would strengthen the conceptual model.

We thank the reviewer for raising this point. A similar point was raised by Reviewer 1, and **we have added a brief discussion of possible routes by which CLN8-generated *S,S*-LPG may reach the lysosome.**

[Redacted]

3. The S,S-LPG rescue experiments are well executed. It may be helpful to more explicitly distinguish functional rescue from complete restoration of endogenous lipid diversity and remodeling dynamics.

We thank the reviewer for this helpful point. **We have clarified in the Discussion that the *S,S*-LPG experiments demonstrate proof-of-concept functional rescue and biochemical tractability of the pathway, rather than complete restoration of**

endogenous BMP diversity. Achieving this will require further compound optimisation and dedicated translational studies, which are ongoing in our laboratory.

4. Given the neurological focus of Batten disease, a short discussion acknowledging the limitations of tracer delivery across the blood–brain barrier, and how brain BMP pools are maintained, would further align the metabolic data with disease relevance.

We thank the reviewer for this helpful point. We agree that, given the neurological focus of Batten disease, it is important to address the limitations of precursor delivery to the brain and to explain how this influenced our choice of in vivo rescue model.

We have therefore added new mouse data directly assessing BMP formation in the brain after intravenous *S,S*-LPG administration (Extended Data Fig. 9f). These analyses showed that, although *S,S*-LPG robustly restored 18:1-18:1 BMP in the liver of CLN8-deficient mice, recovery in the brain was minimal over the acute treatment window. This suggests limited blood-brain barrier permeability of C18:1-LPG in adult mice under the conditions tested.

We have also expanded the rationale for using zebrafish larvae as an initial neurological rescue model. Zebrafish blood-brain barrier formation begins at approximately 72 hpf but does not fully mature until around 10 days post-fertilisation. Therefore, continuous *S,S*-LPG administration from early development provides a window in which the compound may access the nervous system more readily than in adult mice. **We now discuss these points in the Results and Discussion to align the metabolic rescue data more clearly with disease relevance and with the limitations of brain delivery.**

5. In several experiments, exogenous lipid substrates or tracers are applied at concentrations and for durations that may exceed endogenous levels or turnover rates. While appropriate for establishing enzymatic specificity and pathway connectivity, a brief discussion distinguishing demonstration of pathway capability from physiological contribution would help prevent overinterpretation.

We thank the reviewer for this helpful suggestion. **We have clarified that these experiments are designed to establish the pathway, enzymatic specificity and connectivity, rather than to define endogenous flux under physiological conditions.** The mechanisms regulating physiological pathway flux and intermediate trafficking between organelles remain important questions for future work.

6. Most mechanistic experiments are performed in HeLa and HEK293-derived systems, which may not fully capture the lipid metabolic context relevant to neurodegeneration. Given the otherwise high level of rigor and depth in the study,

the limited use of more disease-relevant cellular models stands out and warrants discussion.

We agree with the reviewer that HeLa and HEK293 systems have limitations. These cell models were chosen for their genetic tractability and suitability for mechanistic dissection of the CLN8-dependent enzymatic step, which we expect to be conserved across cell types. Nevertheless, **we have added a brief discussion noting that future studies in CLN8-deficient neuronal models and organoids will be important for assessing disease-relevant lipid metabolism and prioritising *S,S*-LPG-based rescue compounds before in vivo testing.**

Reviewer #4 (Remarks to the Author):

Summary

In this manuscript, Sheokand, Lacabanne, and James et al., sought to demonstrate that CLN8 is a GPG acyltransferase that catalyses stereospecific acylation of S,S-GPG to S,S-LPG through a synergistic approach encompassing Biochemical analysis, cryo-EM, and in vivo model systems. Whilst overall the biochemistry appears sound, and the story is interesting, this reviewer has several comments/queries as to how the cryo-EM structures presented (despite decent quality maps) support the ping-pong acyl transfer mechanism being proposed, and substrate preferences.

1. The authors explain that “a covalent H138-acyl linkage is thermodynamically required for catalysis after CoA-SH dissociation from the active site”. However, no such linkage is observed despite the absence of CoA-SH in the active site in either of the two structures solved in the presence of C18:1/C22:6. While this reviewer is aware that these lipids are highly flexible which can interfere with cryo-EM map quality, the covalent linkage should not be flexible and as such should be clearly visible in these maps. Since this is not observed, this reviewer fails to see how these cryo-EM structures “support a ping-pong acyl-transfer mechanism in which CLN8 catalysis proceeds via a covalent H138–acyl intermediate”, if anything, they call this proposition into question.

Fig. 1 C18:1 and C22:6 densities.

We thank the reviewer for this important point. We agree that the absence of continuous density between H138 and the acyl chain means that the cryo-EM maps alone do not directly visualise the covalent H138-acyl linkage. We have therefore revised the text to make clear that the structures alone do not establish this linkage. Instead, we now present the structural data alongside evidence from functionally homologous proteins and our biochemical experiments as support for a ping-pong acyl-transfer mechanism.

To investigate why the H138-acyl covalent intermediate is not resolved by cryo-EM, **we performed a cavity analysis measuring the solvent-accessible volume within an 8 Å radius centred on the Cα of the catalytic histidine, comparing CLN8 with other structurally characterised homologues and paralogues.** Water molecules were placed into the cryo-EM maps of both the C18:1-CoA and C22:6-CoA states using phenix.douse and manually pruned by inspection against the map in Coot, directly confirming the presence of ordered water molecules within the active site cavity. This analysis showed that, in CLN8, the volume around H138 is 27.88 $Å^3$ in the apo state and increases to 55.62 $Å^3$ and 43.75 $Å^3$ upon C18:1-CoA and C22:6-CoA binding, respectively. By contrast, the equivalent cavity in ceramide synthase CERS6 ranges from 0.38 to 8.63 $Å^3$ across the available structures (PDB: 8QZ6, 8QZ7, 9EOT), and is similarly near-zero in Lac1–Lip1 (1.88 $Å^3$, PDB: 8IZD) and ELOVL7 (2.13 $Å^3$, PDB: 6Y7F). **Thus, the CLN8 active site is one to two orders of magnitude more solvent-exposed than in the homologues examined.**

This has a plausible chemical consequence. The N-acyl histidine intermediate formed after CoA-SH departure is an acyl-imidazole species and is therefore inherently susceptible to hydrolysis. In ceramide synthases and ELOVL7, burial within a hydrophobic, largely anhydrous cavity is expected to protect this intermediate. In CLN8, by contrast, the open, water-accessible active site would make the intermediate kinetically labile under the aqueous conditions and timescales of cryo-EM sample preparation, explaining why continuous density for the H138-acyl linkage is not observed in the C18:1- or C22:6-bound maps.

Importantly, the proposed covalent intermediate is supported independently by our fluorescence trapping assay (Fig. 2d,e), in which an NBD-C18:1-CLN8 adduct is detectable under controlled short-incubation conditions and is lost upon addition of *S,S*-GPG. **We have updated the manuscript accordingly to state more precisely that the cryo-EM structures are consistent with an acyl-bound state, whereas the covalent nature of the intermediate is supported by the biochemical trapping assay and by homology to related acyltransferases.**

<u>We also note that the structural data still support a ping-pong acyl transfer mechanism</u>. In this mechanism, the two substrates bind sequentially rather than simultaneously. In our substrate-bound structure, the acyl chain is present within the catalytic cavity after CoA has been cleaved, leaving space for *S,S*-GPG to access the active site. This corresponds to the expected post-acyl-donor, pre-acceptor state of a ping-pong reaction. By contrast, if the acyl donor remained bound as intact acyl-CoA, the CoA moiety would occupy the cytoplasm-facing cavity and sterically occlude binding of *S,S*-GPG. Thus, although we have revised the text to avoid overstating the evidence for a covalent His-acyl intermediate, **the observed substrate-bound state remains consistent with sequential substrate binding and supports a ping-pong mechanism independently of whether a covalent acyl-enzyme intermediate is directly detected.**

We have added the following explanation to the manuscript text, together with a new Extended Data Fig. 6 and Supplementary Table 2:

Although the structures capture an acyl chain positioned next to H138, continuous covalent density between H138 and the acyl chain was not resolved (Extended Data Fig. 5b,c; Extended Data Fig. 6d,e). This may reflect the hydrolytic lability of an N-acyl-histidine intermediate under aqueous cryo-EM sample preparation conditions. Consistent with this possibility, cavity analysis revealed that the solvent-accessible volume surrounding H138 in CLN8 is one to two orders of magnitude larger than the equivalent cavities in other acyltransferase enzymes with closely related structures, including CERS6 (Supplementary Table 2). This solvent-exposed architecture of CLN8 active site is consistent with the highly hydrophilic nature of the acyl acceptor S,S-GPG, and contrasts with related acyltransferases in which the acyl intermediate is stabilised within a more protected hydrophobic environment (Supplementary Table 2). In agreement with this analysis, ordered water molecules could be successfully modelled within the active site cavity in both the C18:1-CoA and C22:6-CoA cryo-EM maps (Extended Data Fig. 6f-k). This solvent exposure could render an acyl-imidazole intermediate kinetically labile in solution, accounting for the absence of continuous covalent density in the cryo-EM maps while remaining consistent with the H138-dependent NBD-C18:1-CLN8 covalent adduct observed by in-gel fluorescence (Fig. 2d,e).

Further supporting this catalytic model, addition of S,S-GPG to CLN8 preincubated with C22:6-CoA yielded only the apo state in cryo-EM, consistent with acyl transfer and S,S-LPG product release. Together, these observations indicate that CLN8 uses sequential substrate binding and supports a ping-pong acyl transfer mechanism, with H138 playing a key catalytic role analogous to the active-site histidines of related TLCD acyltransferases (Fig. 2h)3,21.

Structure	Catalytic His	Volume excl. ligands & water (Å³)	Volume excl. water only (Å³)
CLN8 + C18:1-CoA	H138	55.62	38.12
CLN8 + C22:6-CoA	H138	43.75	25.62
CLN8 apo	H138	27.88	27.88
ELOVL7 (6Y7F)	H150	12.88	2.13
CERS6 (8QZ6)	H211	6.00	4.88

CERS6 + fumonisin B1 (8QZ7)	H211	34.63	0.38
CERS6 (9EOT)	H211	8.63	4.00
Lac1-Lip1 (8IZD)	H255	32.88	1.88

Supplementary Table 2. *Solvent-accessible cavity volumes around the catalytic histidine residues of CLN8 and related structural homologues.*

Materials and Methods:

Water molecules were added to cryo-EM maps of the C18:1-CoA and C22:6-CoA states using phenix.douse (1) and manually pruned by inspection against the map in Coot (2). Cavity volumes were determined using MDAnalysis (3), by measuring the solvent-accessible volume within an 8 Angstrom radius sphere centered on the Cα atom of the catalytic His residue.

1. Liebschner D, Afonine PV, Baker ML, Bunkoczi G, Chen VB, Croll TI, et al. Macromolecular structure determination using X-rays, neutrons and electrons: recent developments in Phenix. Acta Crystallogr D Struct Biol. 2019;75(Pt 10):861-77.

2. Emsley P, Lohkamp B, Scott WG, Cowtan K. Features and development of Coot. Acta Cryst D. 2010;66(Pt 4):486-501.

3. N. Michaud-Agrawal, E. J. Denning, T. B. Woolf, and O. Beckstein. MDAnalysis: A Toolkit for the Analysis of Molecular Dynamics Simulations. J. Comput. Chem. 32 (2011), 2319-2327, doi:10.1002/jcc.21787. PMCID:PMC3144279

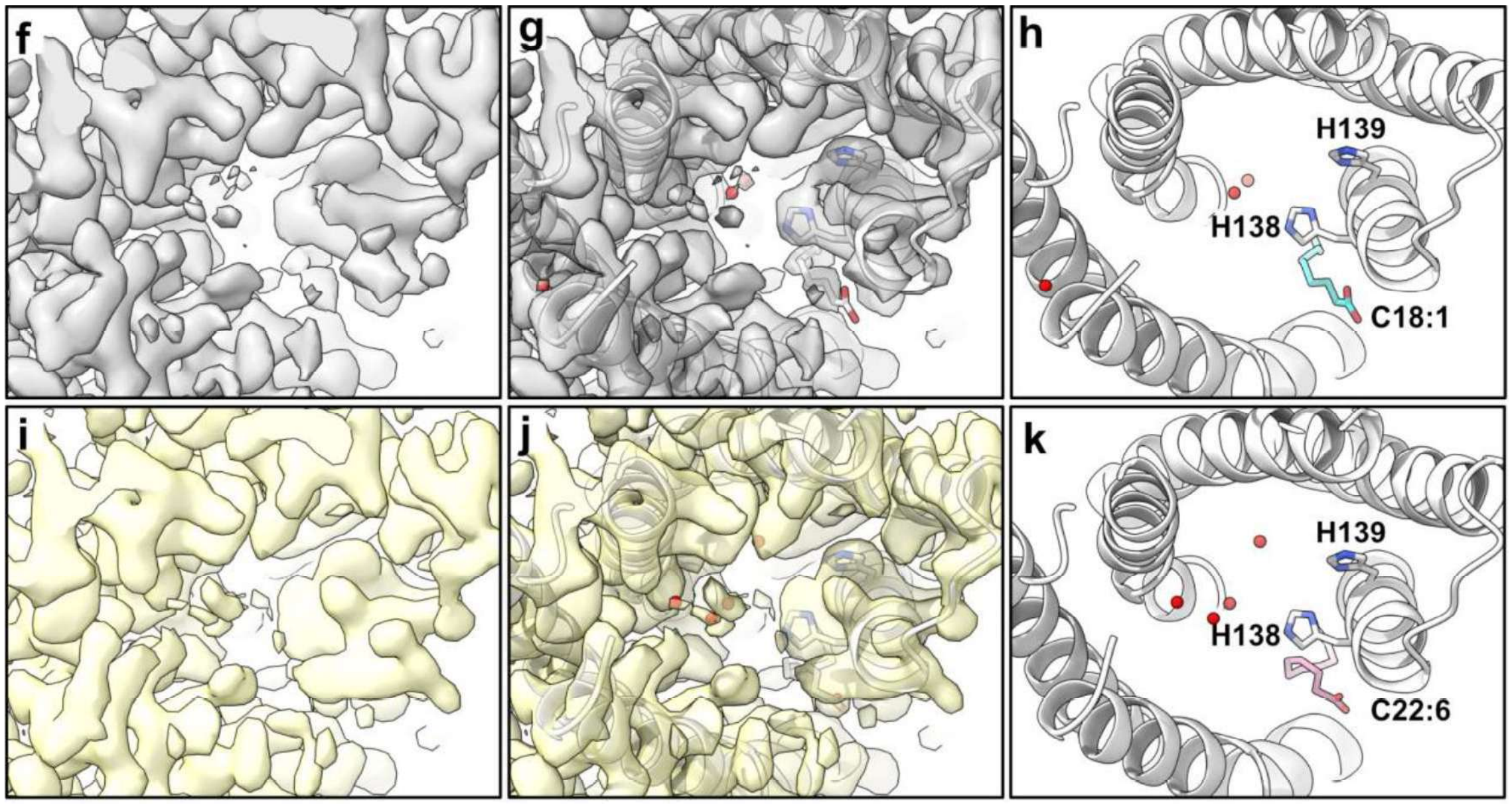

Extended Data Fig. 6. f-k, Close-up views of the catalytic site centred on H138 and H139, showing ordered water molecules modelled within the active-site cavity. Cryo-EM density alone is shown for the C18:1-bound (**f**; contoured at 10.7 σ) and C22:6-bound (**i**; contoured at 10.9 σ) structures. Overlay of density and model is shown for the C18:1-bound (**g**) and C22:6-bound (**j**) structures. The model alone, with labelled active-site residues and bound acyl chain, is shown for the C18:1-bound (**h**) and C22:6-bound (**k**) structures. Water molecules are shown as red spheres.

2. Could the authors please explain why the density fit for C22:6 is worse than that for 18:1 when it is the preferred substrate? This seems counter-intuitive.

We thank the reviewer for this question. Although CLN8 shows a catalytic preference for C22:6-CoA, substrate preference does not necessarily imply a more ordered acyl chain density in the cryo-EM map. The weaker C22:6 density likely reflects the greater conformational flexibility of the polyunsaturated acyl chain. Its six cis double bonds introduce multiple kinks, allowing the chain to sample several binding poses within the cavity. This conformational heterogeneity would be averaged during reconstruction, producing weaker and less continuous density. By contrast, the monounsaturated C18:1 chain is more conformationally restricted and therefore adopts a more ordered binding mode, giving better-resolved density. **We have clarified this point in the revised manuscript.**

3. The authors elected to position the acyl chain was positioned with a carboxy group adjacent to H138 within the central cavity and the tail oriented toward the ER lumen. The reasoning for this is not provided, and it is not unambiguous based on the stereochemistry of the binding site. I anticipate that this orientation was selected

based on the functional results presented in Fig. 2c and the assumption of a ping-pong mechanism. However, this reviewer questions this choice on two grounds: (a) the H138A mutation could disrupt substrate binding via the acyl tail without being a catalytic residue that forms a covalent adduct with the substrate (especially given the varying preference for substrates with different tail lengths presented in Fig 1k.); and (b) the cryo-EM maps provided overlap do not support a ping-pong mechanism. More evidence is required to support the orientation of the fit.

We thank the reviewer for this comment. We agree that the basis for orienting the acyl chain should be stated more explicitly.

The acyl chain orientation was not assigned solely from the H138A functional data or from assuming a ping-pong mechanism. Rather, it is constrained by the architecture and chemical organisation of the catalytic cavity. The density within the cavity forms a narrow, elongated feature consistent with an acyl chain occupying a hydrophobic channel. The conserved H138-H139 motif lies at the junction between this narrow hydrophobic channel and a larger cavity on the cytoplasmic side of the protein. Placing the acyl carboxyl group adjacent to H138-H139 therefore positions the polar/reactive end at the catalytic interface, while the hydrocarbon tail extends into the hydrophobic channel. This orientation is consistent with the shape of the density, the physicochemical properties of the pocket, and the acyl chain orientation observed in the CLN8 paralogue CERS6 (8QZ6).

This assignment is also the only polarity compatible with binding of the acyl-CoA donor before cleavage. In the modelled orientation, the bulky CoA moiety would occupy the adjacent cytoplasm-facing cavity, which is sufficiently spacious to accommodate it. In the opposite orientation, the CoA group would have to occupy the ER-luminal side of the cavity. This region is structurally restricted by the TM2–TM3, TM4–TM5 and TM6–TM7 loops, and cannot plausibly accommodate the CoA moiety. Moreover, reversing the polarity would place the acyl carboxyl group deep within a predominantly aliphatic environment, away from residues capable of supporting catalysis.

We therefore agree that the cryo-EM density alone does not directly visualise the full acyl-CoA substrate or a covalent acyl-enzyme intermediate. However, the observed ligand density, the topology of the cavity, the position of the conserved H138-H139 motif, and the requirement to accommodate the CoA leaving group together strongly support the orientation presented in the model.

We have added the following sentence to the Materials and Methods section, “Model building, validation, and structural analysis”:

The acyl chain was oriented with its carboxyl group adjacent to H138-H139 because this places the hydrocarbon tail within the narrow hydrophobic channel and, importantly, is the only polarity compatible with accommodation of the bulky CoA moiety in the adjacent cytoplasm-facing cavity prior to cleavage.

4. The model fit for the apo structure is poor and appears to be quite off, this is most notable in TM4 which contains several residues important for substrate binding and should be revisited with scrutiny and care – it may well be that this helix is rotated in the apo state compared to the substrate boundstates, but this has not been elucidated due to poor model fitting.

We thank the reviewer for this careful inspection of the apo structure. We agree that the local density around TM4 in the apo map is less well resolved than in the substrate-bound structures, consistent with the lower overall resolution of the apo reconstruction (~3.7 Å) and increased local flexibility in this region.

We therefore re-examined this part of the model in detail. At low contour levels, weak density features are visible near TM4 that could, in isolation, be interpreted as side-chain density, including near A142 and G145 (**Reviewer Fig. 2a,b**). However, these features disappear more rapidly than the surrounding protein density as the threshold is increased, indicating that they are not well-defined side-chain densities. Moreover, comparison with the higher-resolution C18:1- and C22:6-bound maps shows that these residual apo-map features overlap spatially with the position of the bound acyl chains in the substrate-bound structures (**Reviewer Fig. 2c,d**). We therefore consider it more likely that they represent low-occupancy, heterogeneous lipid or detergent density retained during purification, rather than ordered protein atoms. At this resolution, we do not think such weak features can be assigned confidently to ordered solvent molecules.

We also tested alternative local fits of TM4, including changes in helix orientation. These alternatives did not improve agreement with the density and led to poorer model geometry. Importantly, the register and orientation of TM4 are constrained by clear density for key residues, including H138 and H139, and the apo TM4 density overlays well with the corresponding region in the higher-resolution substrate-bound structures (**Reviewer Fig. 2e**). Thus, although local density in this part of the apo map is weaker, the available evidence does not support a substantial rotation or mismodelling of TM4 in the apo state.

Based on this re-evaluation, we have retained the original model. We consider the apo TM4 model to be conservatively fitted within the limits of the local map quality, and the mechanistic conclusions of the manuscript are primarily supported by the higher-resolution substrate-bound structures, in which this region is better resolved.

Reviewer Fig. 2. Evaluation of TM4 density in the apo CLN8 structure (next page). a,b, TM4 region of the apo structure contoured at low (a, 0.105) and higher (b, 0.175) thresholds, highlighting weak residual density features near TM4. **c,d,** Overlay of apo density, shown as a surface, with the C18:1- and C22:6-bound maps, shown as black mesh, indicating that residual features in the apo map coincide with the position of bound acyl chains in the substrate-bound structures. **e,** Overlay of

TM4 density from the apo structure, shown as a surface, with higher-resolution substrate-bound maps, shown as mesh, demonstrating consistent helix positioning. Key residues are indicated.

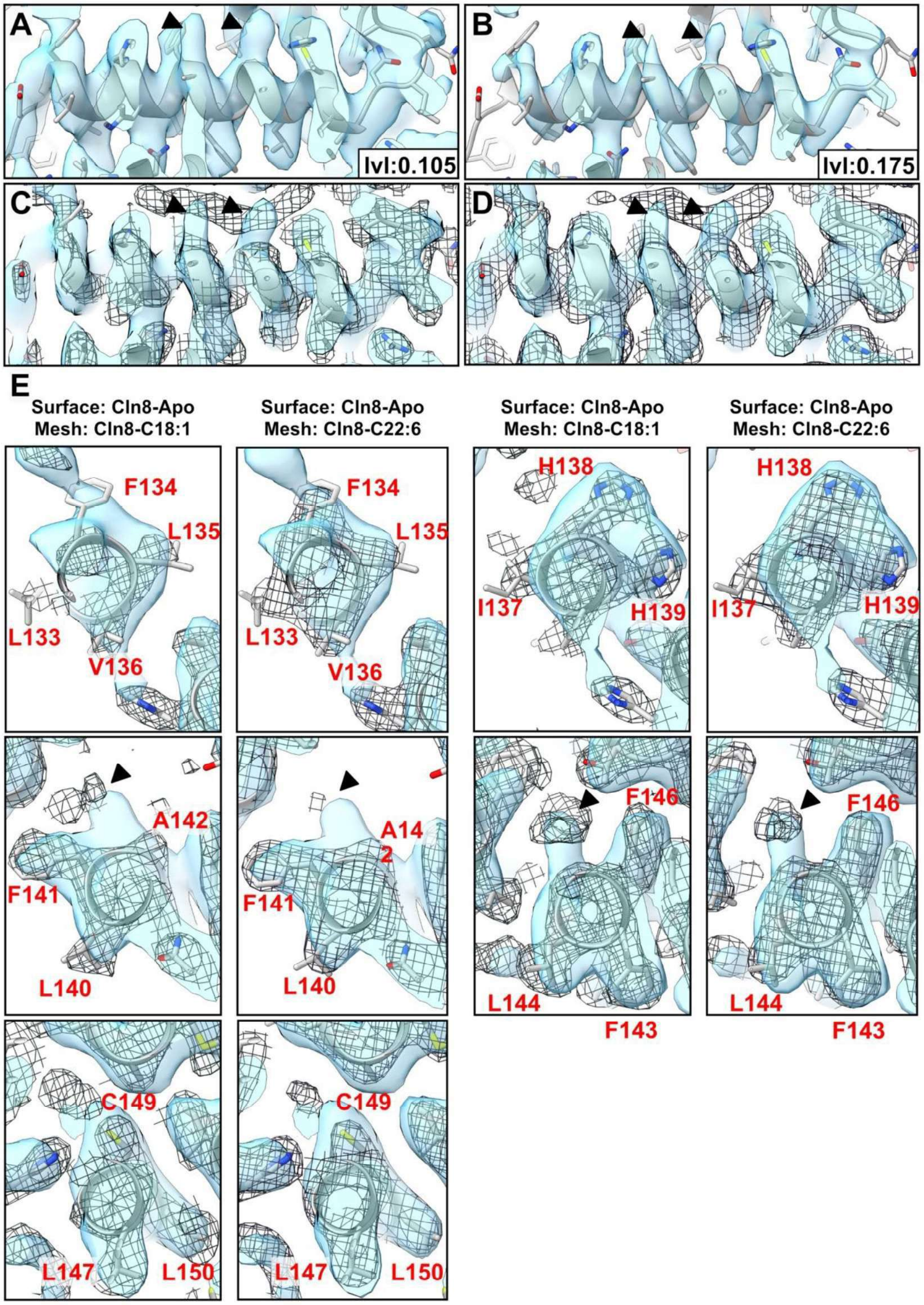

5. While the substrate donor contains a CoA (C18:1-CoA and C22:6-CoA), the authors do not carefully assess the CoA binding sites or explain how the position of the CoA binding site aligned with the proposed mechanism. When assessing the map quality and model fits, I\this reviewer made three noteworthy observations that require further assessment:

a. There is an additional unfit density extending from the phosphate groups of the CoA in both the C18:1 and C22:6 bound structures, it is very consistent across the 4 CoA binding sites in the C18:1/C22:6 bound structures and thus can't be noise… what is it?

We thank the reviewer for highlighting this feature. We agree that the additional density extending from the phosphate groups appears reproducible when applying C2 symmetry and therefore initially does not resemble random noise. However, we note that the reconstruction was performed with imposed C2 symmetry, which enforces identical features across the two protomers and can artificially enhance weak or non-conserved signal, particularly in regions close to the symmetry axis where this feature is located.

To address this point, we re-examined the region using a reconstruction without imposed symmetry (C1). In these maps, the additional density is no longer consistently observed across equivalent sites and appears fragmented and variable (**Reviewer Fig. 3**), indicating that it does not correspond to a well-defined, conserved structural feature. This suggests that the apparent density in the symmetrised maps likely arises from averaging of weak or heterogeneous signal in a locally noisy region. While the overall density is consistent with the presence of CoA (in agreement with our mass spectrometry data presented below) the additional feature branching from the phosphate group cannot be reliably assigned at the current resolution. We therefore consider it to represent unmodelled, low-occupancy or heterogeneous density and have chosen not to model it to avoid overinterpretation.

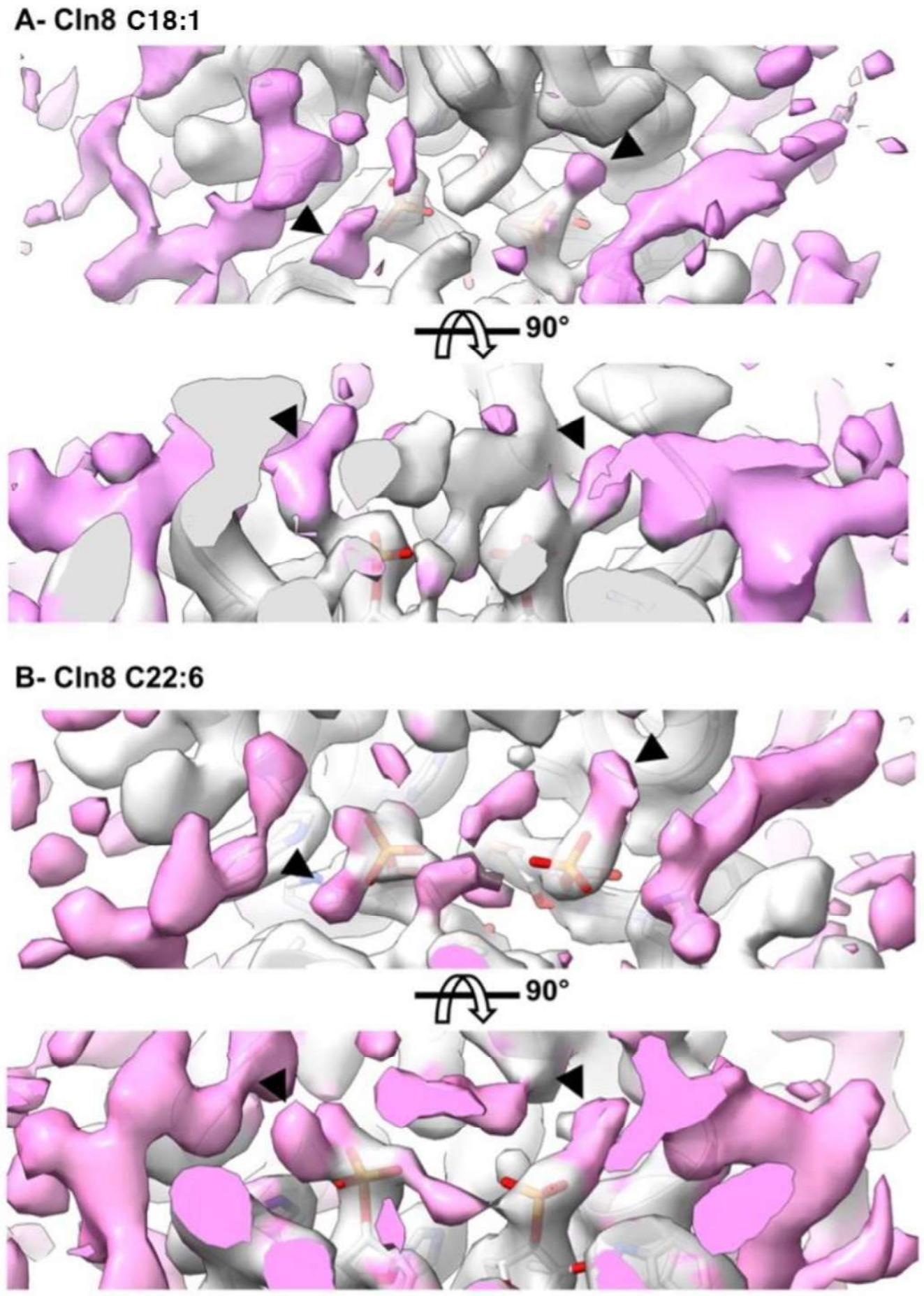

Reviewer Fig. 3. Assessment of additional density near the CoA phosphate group using C1 reconstruction. Close-up views of the CoA-binding site in the C18:1 (**a**) and C22:6 (**b**) bound structure reconstructed without imposed symmetry (C1), shown in two orientations related by a 90° rotation (top view – top-panel and side view – bottom panel) . The additional density adjacent to the CoA phosphate group, previously observed in symmetrised (C2) maps, is indicated by black arrowheads. Cryo-EM maps are displayed as semi-transparent surfaces with their respective models fitted. The map region within 2.0 Å of any fitted model atom is shown in grey (coloured using the Color Zone function in ChimeraX with a radius of 2.0 Å), density beyond this radius is shown in pink, highlighting regions of the map not accounted for by the current model.

b. There is a large additional density adjacent to all 4 CoA binding sites in the C18:1/C22:6 bound structures. The shape of this density is consistent across the 4 sites and so again, it can't be noise, what is it?

We thank the reviewer for highlighting this feature. In our cryo-EM maps, additional peripheral densities are observed at several positions on the protein surface, including adjacent to the CoA binding sites. Importantly, these densities are spatially

separate and not contiguous with the CoA density when maps are viewed at appropriate contour levels. Similar densities are present elsewhere around the transmembrane region and are not connected to the polypeptide backbone (**Reviewer Fig. 4**). Based on their shape, size and location, we interpret these features as detergent or residual lipid molecules from the micellar environment used for protein purification and grid preparation. In particular, the squarish density noted by the reviewer is consistent with the maltose headgroup of LMNG, as described in other cryo-EM structures of membrane proteins solved in LMNG micelles (e.g. the mycolic-acid transporter MmpL3; PDB 7NVH, EMDB EMD-12604). Because these densities are heterogeneous and not directly related to the functional sites under investigation, we chose not to model them.

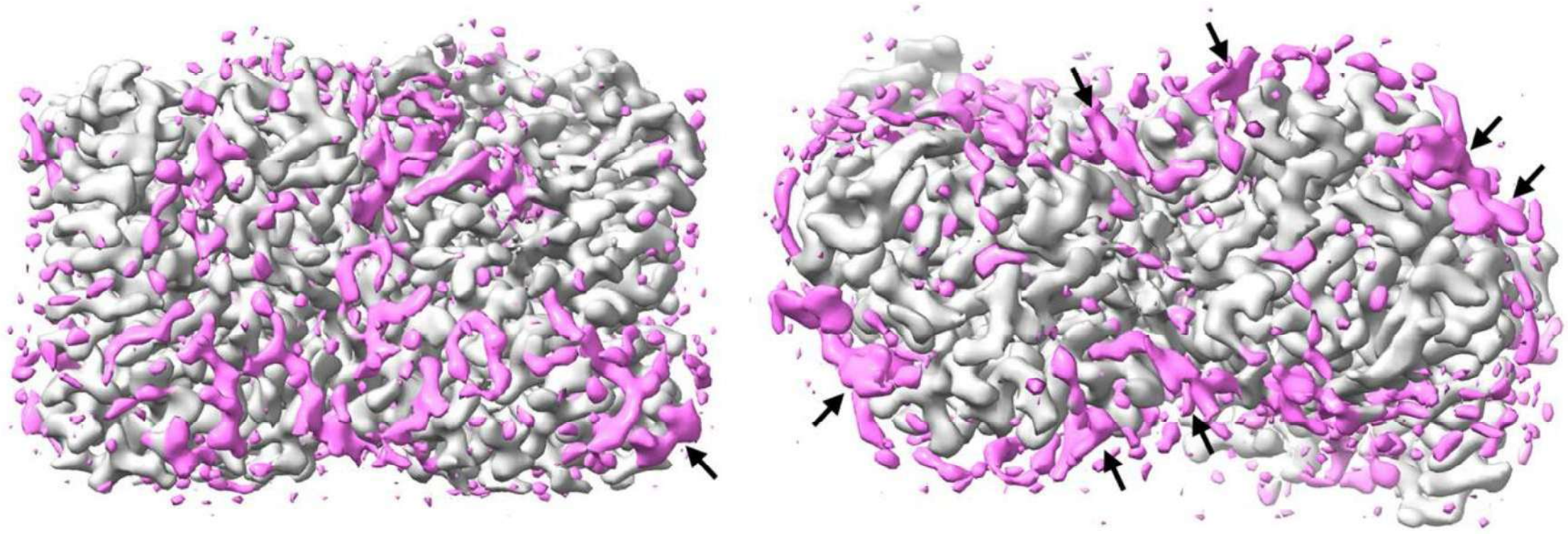

Reviewer Fig. 4. Additional peripheral densities in CLN8 cryo-EM maps are consistent with detergent molecules. Cryo-EM maps of the CLN8 C18:1-CoA-bound side view (left) and top view (right) densities. Map regions within 2.0 Å of any fitted model atom are shown in grey ; density beyond this radius is shown in pink, highlighting features not accounted for by the current model (Color Zone, ChimeraX, radius 2.0 Å). Black arrows indicate additional peripheral densities interpreted as detergent or lipid molecules from the LMNG micellar environment used during protein purification and grid preparation.

c. The density CoA binding site in the apo structure is very poor compared to that in the C18:1/C22:6 bound structures – I know this structure is lower in resolution, but this density could be a different lipid/misfit loop… how are the authors certain that this is CoA given this poor fit?

Performing additional experiments (e.g., native mass spec) to confirm presence/absence of CoA in the apo state, and to confirm if CoA (without chemical modification of the phosphate group) is/isn't bound in the C18:1/C22:6 bound structures. Mass spec may also provide insights into whether a covalent his138 conjugate is being formed or not which could help address issues in Major Point 1.

We thank the reviewer for this important point. We agree that the density at the CoA binding site in the apo structure is of lower quality compared to the higher-resolution C18:1- and C22:6-bound structures (~2.7-3.2 Å), reflecting the overall lower resolution of the apo map (~3.7 Å). However, despite this, the density retains characteristic features consistent with CoA, including density corresponding to the adenosine moiety, the 3′-phosphate/pyrophosphate region, and the pantothenate core. These features are positioned identically to those observed in the higher-resolution structures, where CoA can be assigned with high confidence. Importantly, the density is not compatible with a misfitted loop, as the surrounding protein backbone is well resolved and independently constrained, and no alternative polypeptide trace can account for this feature. Furthermore, the overall shape and connectivity of the density are not consistent with typical membrane lipids.

[Redacted]

Minor Points

• These densities for 18:1, 22:6, and CoA presented in the supplementary data are somewhat deceptive compared to what is observed in the .mrc files. These densities should be presented in a manner which truly depicts their quality and alignment with the model (as is shown here in Fig. 1 and 3).

We have revised the figures to display the density for CoA and the acyl chains (C18:1 and C22:6) using contour levels and representations that more accurately reflect the underlying map quality and its agreement with the model. These updated figures now present the apo, C18:1- and C22:6-bound states in a consistent manner and allow a direct assessment of density quality and model fit **(new Extended Data Fig. 6a-e).**

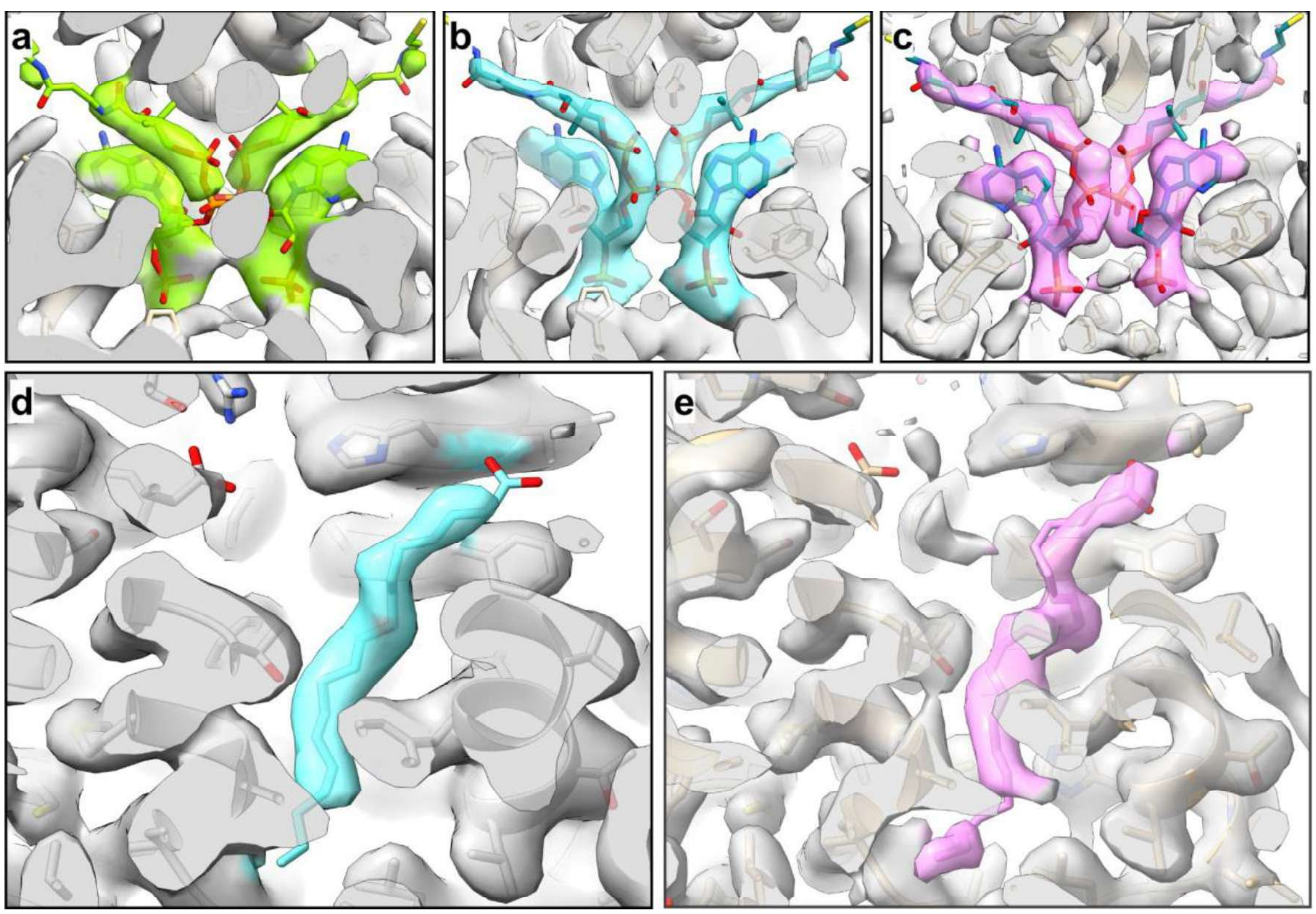

Extended Data Fig. 6: Cryo-EM density of CoA, bound acyl chains and ordered water molecules in the CLN8 active site. a-c, Cryo-EM density corresponding to CoA in the apo (**a**), C18:1-CoA-bound (**b**) and C22:6-CoA-bound (**c**) structures, shown at comparable contour levels of 8.0 σ, 11.4 σ and 9.0 σ, respectively,

illustrating map quality and agreement with the fitted model. **d,e,** Cryo-EM density of the bound acyl chains in the C18:1-bound (**d**) and C22:6-bound (**e**) structures. In **a-e**, protein density is shown in grey, ligands are coloured cyan for the C18:1-bound structure and light pink for the C22:6-bound structure, and cryo-EM maps are displayed as semi-transparent surfaces.

• This statement in the abstract is misleading: "Using cryo-electron microscopy, we resolve structures of the CLN8 homodimer in apo and substrate-bound states at 2.7 Å resolution, revealing the active site architecture and a ping-pong acyl transfer mechanism." The apo structure is 3.6A in resolution, and the cryo-EM structures do not support a ping-pong acyl transfer mechanism.

We thank the reviewer for this appropriate criticism. We agree that the original wording was imprecise because the apo structure was resolved at 3.6 Å, whereas the substrate-bound structure reached 2.7 Å. We have therefore revised the abstract to remove the resolution values from this sentence.

We also agree that the cryo-EM maps do not directly visualise a covalent His-acyl intermediate. However, we respectfully note that the structural data still support a ping-pong acyl-transfer mechanism. In this mechanism, the two substrates bind sequentially rather than simultaneously. In our substrate-bound structure, the acyl chain is present within the catalytic cavity after CoA has been cleaved, leaving space for *S,S*-GPG to access the active site. This corresponds to the expected post-acyl-donor, pre-acceptor state of a ping-pong reaction. By contrast, if the acyl donor remained bound as intact acyl-CoA, the CoA moiety would occupy the cytoplasm-facing cavity and sterically occlude binding of *S,S*-GPG. Thus, although we have revised the text to avoid overstating the evidence for a covalent His-acyl intermediate, the observed substrate-bound state remains consistent with sequential substrate binding and supports a ping-pong mechanism.

• At present, the only residue that is in a different conformation in the binding sites between the three structures presented is F134 in the C18:1 bound state. However, this fit is not supported by the cryo-EM map and should be revisited.

We thank the reviewer for spotting this inconsistency. We have revisited the fit of F134 in the C18:1-bound structure and agree that the previous rotamer was not optimally supported by the cryo-EM density. The residue has now been remodelled to better match the map, and the updated coordinates and maps have been uploaded to the PDB.

The atomic models for the C18:1-CoA-bound and C22:6-CoA-bound CLN8 structures have been updated to incorporate the revisions described above, including the addition of ordered water molecules. The revised coordinates have

been deposited to the Protein Data Bank and are currently awaiting final validation and release. The updated model files (.pdb) and corresponding cryo-EM density maps are also provided as part of the submitted .zip archive for the reviewers' inspection.

Reviewer #4 (Remarks on Protocol(s)):

Protein purification and cryo-EM collection/analysis are sound.

Response to reviewers – Second revision

Sheokand, Lacabanne, James *et al*, 2026

Reviewer #1 (Remarks to the Author):

The authors have done an excellent job of addressing my technical concerns, particularly those regarding the mass spectrometry measurements of BMP lipids. Their revisions and additional clarifications have significantly strengthened the analytical rigor of the manuscript. I congratulate the authors on their compelling findings.

Reviewer #2 (Remarks to the Author):

The authors have adequately addressed my concerns regarding the previous version of their manuscript, except my question [7]. I asked the authors to compare and contrast, in the Discussion, their findings with those of Singh et al. (https://doi.org/10.1016/j.cell.2024.09.036), to point out in some detail how the approaches of Singh et al. led to insights that are different from those of their manuscript. In reply, the authors have added this phrase "Although PLD3/4 has also been proposed as an alternative to CLN5 for catalysing this final lysosomal step [43], ..." This sentence underplays the differences between the authors' study and that of Singh et al. I would like the authors to expand on these differences in their Discussion.

We thank the reviewer for raising this point again. We agree that the relationship between the CLN5-dependent pathway model used in our manuscript and the PLD3/4-dependent model proposed by Singh et al. should be stated more clearly. We have therefore expanded the Discussion to distinguish the unresolved lysosomal BMP-forming reaction from the upstream CLN8 reaction defined in our study.

'Our pathway model builds on the previously described CLN5-dependent lysosomal transacylation reaction, in which two LPG molecules are converted to BMP in the lysosomal lumen (Fig. 5)[4]. An alternative model proposes that PLD3 and PLD4 enzymes generate BMP through a distinct lysosomal transphosphatidylation reaction between LPG and monoacylglycerol[44]. Both models therefore involve LPG as a substrate but differ in how mature BMP is formed. Our study addresses the preceding step: the acyl-CoA-dependent generation of LPG by CLN8 outside the lysosome. Thus, our identification of CLN8 defines how the LPG intermediate is produced, while the precise lysosomal reaction by which LPG is subsequently converted to BMP remains to be resolved in future work.'

This addition explicitly contrasts the CLN5-dependent transacylation model with the PLD3/4-dependent transphosphatidylation model, while clarifying that our study defines the upstream acyl-CoA-dependent generation of LPG by CLN8. Given space constraints, the focus of our manuscript on the CLN8 reaction, and the fact that our Discussion already covers several other important aspects of the study, we have kept this comparison concise. A full comparison of the CLN5 and PLD3/4 models would be lengthy and would not fit within the scope of the present Discussion. Nevertheless, we agree with the reviewer that this is an important unresolved question, and we now highlight it clearly in the Discussion.

Reviewer #3 (Remarks to the Author):

The authors have sufficiently addressed my comments and concerns. thank you.

Reviewer #4 (Remarks to the Author):

I thank the authors for their thorough and careful responses to my previous comments. I am satisfied with how the majority of points have been addressed. However, I remain concerned regarding the quality of the model built into TM4, and do not feel this issue has been adequately resolved.

The additional densities observed in this region appear intimately associated with the helix backbone, making an assignment to a lipid or detergent molecule unlikely on geometric grounds alone. Furthermore, the morphology of these densities is not consistent with what one would typically expect for either lipid or detergent, and I therefore do not find this interpretation convincing.

I strongly recommend that the authors undertake additional cryo-EM data processing efforts specifically targeting this region before this manuscript can be considered further. This should include focused classification without alignment, local refinement centred on TM4, multibody refinement, density subtraction-based focused refinement, and careful optimisation of per-particle defocus refinement and Bayesian polishing for this region. The authors should additionally consider the ab initio reconstruction workflow of Kim, Li & Clarke (bioRxiv, 2025.09.08.674935) as it is particularly good for small proteins.

I consider this analysis particularly important because the poorly resolved density in this region may be masking a genuine conformational change that is not currently being captured; given that this is the apo state, the possibility that TM4 samples an alternative conformation in the absence of substrate would be an interesting finding

in its own right and is worth investigating. If these approaches collectively fail to yield a meaningful improvement in local resolution, the authors should explicitly acknowledge in the manuscript that the registry of TM4 could not be assigned with confidence. Given that this is the apo state – and that the stabilising interactions provided by the substrate (C18/22) are absent – it is reasonable to attribute the lower local resolution in this region to inherent conformational flexibility.

We thank Reviewer 4 for their continued engagement with this issue and for the specific methodological suggestions provided. We appreciate the reviewer's hypothesis that the ambiguous TM4 density may reflect a genuine conformational change in the apo state, and we have taken this seriously in our additional analyses, as we did in the previous ones.

We note that the majority of the approaches recommended by the reviewer had in fact already been performed prior to the first round of review as part of our standard processing pipeline, although not explicitly described in the methods section in that version of the manuscript. The one approach we had not previously considered was the HR-HAIR ab initio reconstruction workflow of Kim, Li & Clarke (bioRxiv, 2025.09.08.674935) and we thank the reviewer for bringing this to our attention. This proved to be the most informative analysis we have performed on the apo dataset, and we describe it in detail below.

Unlike conventional ab initio reconstruction, which operates at low resolution and cannot distinguish subtle structural differences between particles, HR-HAIR uses only high spatial frequency data with a very fine Fourier radius step, allowing accurate particle orientation assignment based on fine structural details while simultaneously separating conformationally distinct particle populations that would otherwise be averaged together. Specifically, we used the following non-default parameters in CryoSPARC: Fourier radius step 0.005, initial resolution 6 Å, maximum resolution 4 Å, 2 classes and 3 classes.

Applying this workflow to our full 260k particle stack revealed a striking and previously undetected heterogeneity in our dataset. The particles separated into two discrete classes:

- **Class 1 (~143k particles):** produces a clean, interpretable map.

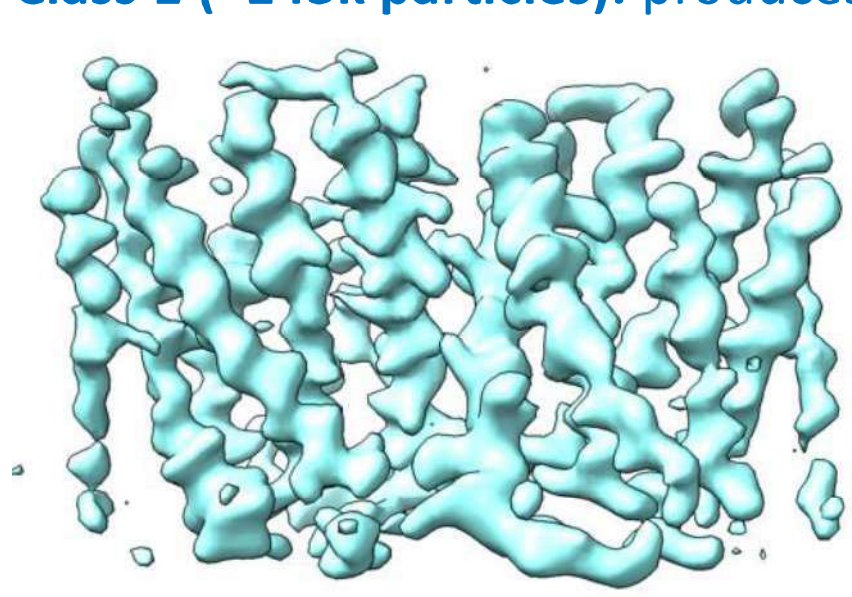

- **Class 2 (~110k particles):** shows partial loss of density at the dimer interface, consistent with conformational heterogeneity in the apo state, possibly reflecting flexibility at the dimer interface in the absence of substrate in line with the reviewer's hypothesis.

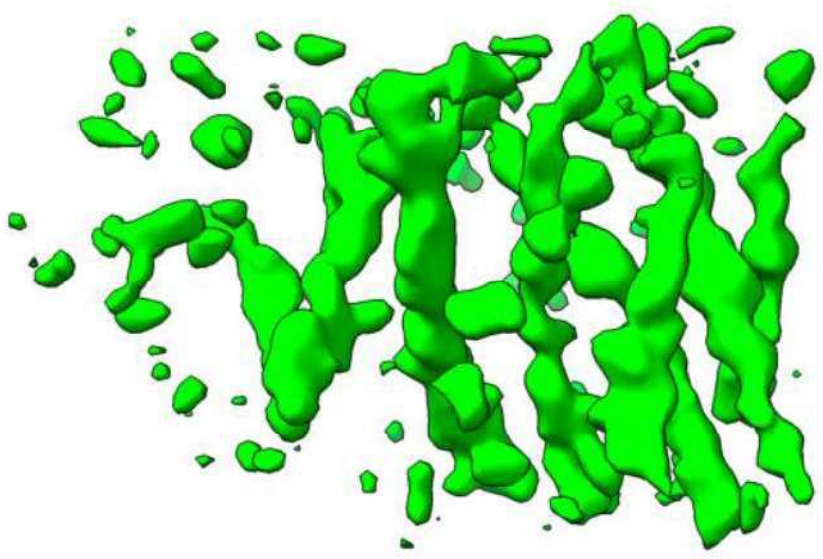

The Class 1 particles have been processed through a local refinement job. The additional densities in the TM4 region that concerned the reviewer are absent in this map, and the model fits well throughout, including TM4. The register and orientation of TM4 are well-defined, consistent with our previously reported model.

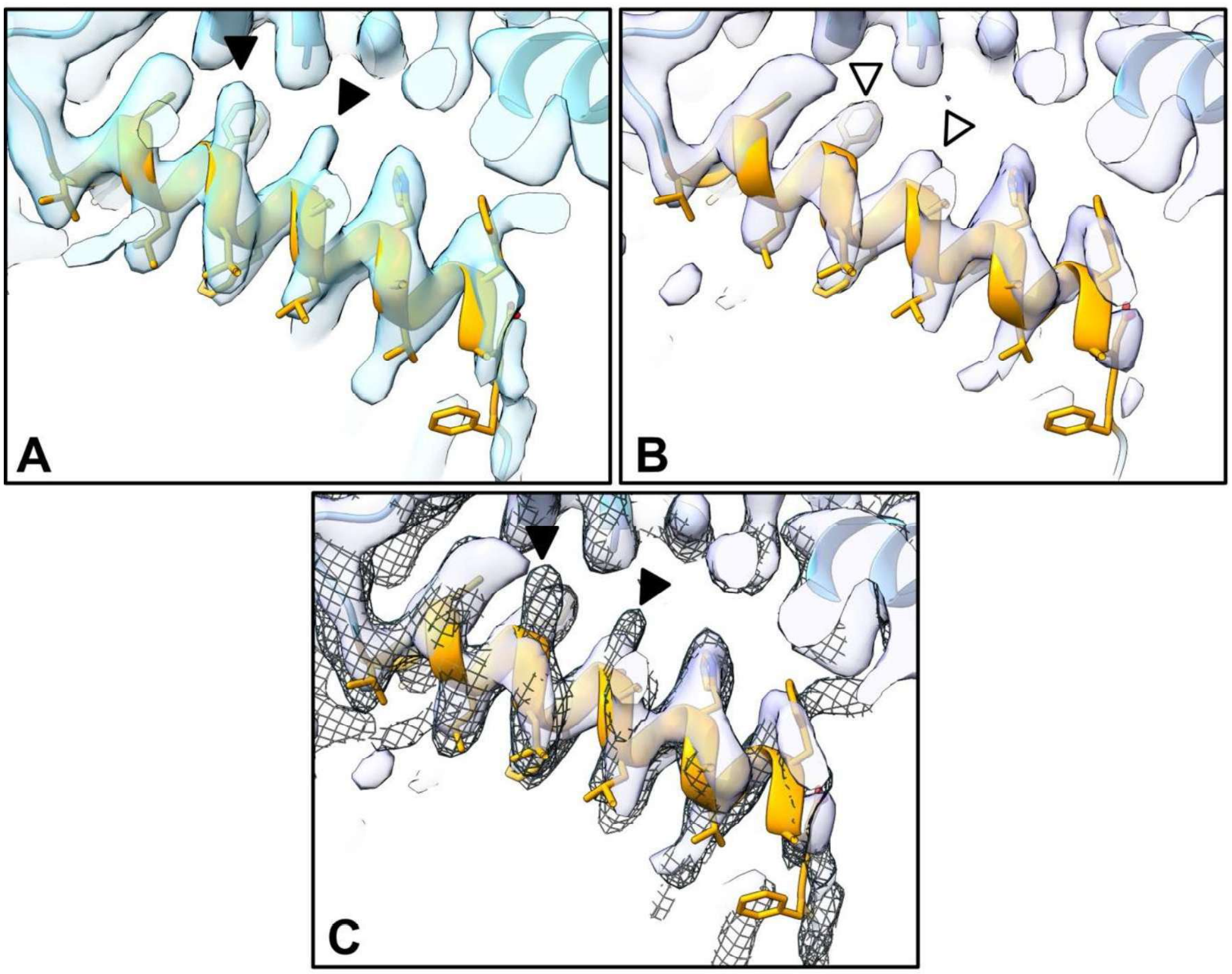

Reviewer Fig. 1. HR-HAIR ab initio reconstruction resolves the ambiguous TM4 density in the CLN8 apo structure. a, TM4 region of the original apo map (260k particles, 3.65 Å) contoured as a transparent surface, with the refined model shown as sticks (orange). Black arrows indicate additional densities intimately associated with the TM4 helix backbone that could not be confidently assigned to protein, lipid, or detergent. **b,** TM4 region of the HR-HAIR-refined map (143k particles, 3.68 Å) at the same contour level. Open arrows indicate the positions where the additional densities were observed in (**a**), demonstrating their

absence in the cleaned map. The model fits well throughout the TM4 region without ambiguity. **c,** Overlay of the original apo map (grey mesh, 260k particles) and the HR-HAIR-refined map (transparent surface, 143k particles) at the same contour level. Black arrows highlight the additional densities present exclusively in the original map, confirming that they arise from the conformationally heterogeneous particle population identified and separated by HR-HAIR rather than from ordered protein or ligand atoms.

The marginal resolution difference between the 143k map (3.68 Å) and the original 260k map (3.65 Å) despite removing nearly half the data demonstrates that the particles in Class 2 were contributing heterogeneous rather than coherent structural signal to the original reconstruction. Comparison of maps shows that their inclusion was responsible for the ambiguous TM4 density that concerned the reviewer. Rather than representing a discrete conformational state, the extra TM4 densities vanish in the 143k map, confirming they were artifacts of particle heterogeneity

Importantly, no discrete alternative conformation of TM4 was identified in either class, supporting the conclusion that the ambiguous density reflects inherent conformational flexibility in the apo state rather than a specific structural state that has been missed. To address whether the separation of particles into two classes reflects genuine conformational heterogeneity rather than differences in imaging quality, we compared key metrics between the two classes. Both classes showed identical defocus distributions (Class 1: mean 1.34 μm ± 0.38 μm; Class 2: mean 1.34 μm ± 0.38 μm), confirming that the particle separation is not attributable to differences in CTF parameters or imaging conditions. Furthermore, alignment quality metrics were virtually identical between the two classes (alignment error Class 1: 6628 ± 224; Class 2: 6639 ± 234), with marginally higher cross-correlation scores in Class 1 (101.7 ± 28.2 vs 97.3 ± 27.3), consistent with greater structural coherence in this class. Subsequent local refinement of Class 1 yielded a well-resolved map at 3.85 Å, whereas local refinement of Class 2 (whether treating the particles as a dimer or focusing on a single monomer) failed to produce a coherent reconstruction, confirming that Class 2 does not represent an alternative discrete conformation of CLN8 but instead comprises genuinely heterogeneous particles that cannot be refined into a meaningful structural model. Together, these analyses confirm that the separation of particles into two classes by HR-HAIR reflects genuine structural heterogeneity within the apo dataset rather than differences in data quality or imaging conditions.

We have revised the manuscript accordingly to explicitly acknowledge the conformational flexibility of TM4 in the apo state, to note that the register of TM4 in the apo structure cannot be assigned with the same confidence as in the substrate-bound structures, and to describe the HR-HAIR analysis and its implications for the interpretation of the apo map.

We updated the Extended Data Fig. 4 to indicate the HR-HAIR ab initio reconstruction workflow for the apo structure.

The apo structure was re-refined against the HR-HAIR-derived map using Servalcat and the updated coordinates and map have been deposited in the PDB and EMDB.

Main text:

'High-resolution heterogeneous ab initio reconstruction (HR-HAIR)[23] of the apo dataset revealed conformational heterogeneity, and the final apo structure was determined from the dominant particle class, in which TM4 is well-defined; the remaining particles could not be refined into a coherent map, consistent with conformational flexibility in this region in the absence of substrate (Extended Data Fig. 4a).'

Materials and Methods:

'For the CLN8 apo dataset, additional processing was performed to investigate conformational heterogeneity in the TM4 region. High-resolution heterogeneous ab initio reconstruction (HR-HAIR)[23] was performed on the full 260k particle stack using CryoSPARC with the following non-default parameters: number of classes, 2; initial resolution, 6 Å; maximum resolution, 4 Å; Fourier radius step, 0.005; initial minibatch size, 300; final minibatch size, 1000; centre structures in real space, OFF. This approach, which uses only high spatial frequency data to assign particle orientations, revealed two discrete particle populations: a dominant class of ~143k particles yielding a 3.68 Å map, and a secondary class of ~110k particles showing partial loss of density at the dimer interface. The dominant class was used for all subsequent processing and model building of the apo structure. Focused local refinement centred on TM4 was additionally performed using a soft mask encompassing the TM4 helix, with rotation search extent 2°, shift search extent 1 Å, and initial lowpass resolution 6 Å.'

Reviewer #4 (Remarks on Protocol(s)):

Yes for structural and cryo-EM analysis
